# What items should be included in an early warning score for remote assessment of suspected COVID-19? qualitative and Delphi study

**DOI:** 10.1136/bmjopen-2020-042626

**Published:** 2020-11-12

**Authors:** Trisha Greenhalgh, Paul Thompson, Sietse Weiringa, Ana Luisa Neves, Laiba Husain, Merlin Dunlop, Alexander Rushforth, David Nunan, Simon de Lusignan, Brendan Delaney

**Affiliations:** 1 Nuffield Department of Primary Care Health Sciences, University of Oxford, Oxford, UK; 2 Department of Experimental Psychology, University of Oxford, Oxford, UK; 3 Institute of Global Health Innovation, Imperial College London, London, UK; 4 Ardens Ltd, Oxford, UK

**Keywords:** infectious diseases, health informatics, virology, covid-19

## Abstract

**Background:**

To develop items for an early warning score (RECAP: REmote COVID-19 Assessment in Primary Care) for patients with suspected COVID-19 who need escalation to next level of care.

**Methods:**

The study was based in UK primary healthcare. The mixed-methods design included rapid review, Delphi panel, interviews, focus groups and software development. Participants were 112 primary care clinicians and 50 patients recovered from COVID-19, recruited through social media, patient groups and snowballing. Using rapid literature review, we identified signs and symptoms which are commoner in severe COVID-19. Building a preliminary set of items from these, we ran four rounds of an online Delphi panel with 72 clinicians, the last incorporating fictional vignettes, collating data on R software. We refined the items iteratively in response to quantitative and qualitative feedback. Items in the penultimate round were checked against narrative interviews with 50 COVID-19 patients. We required, for each item, at least 80% clinician agreement on relevance, wording and cut-off values, and that the item addressed issues and concerns raised by patients. In focus groups, 40 clinicians suggested further refinements and discussed workability of the instrument in relation to local resources and care pathways. This informed design of an electronic template for primary care systems.

**Results:**

The prevalidation RECAP-V0 comprises a red flag alert box and 10 assessment items: pulse, shortness of breath or respiratory rate, trajectory of breathlessness, pulse oximeter reading (with brief exercise test if appropriate) or symptoms suggestive of hypoxia, temperature or fever symptoms, duration of symptoms, muscle aches, new confusion, shielded list and known risk factors for poor outcome. It is not yet known how sensitive or specific it is.

**Conclusions:**

Items on RECAP-V0 align strongly with published evidence, clinical judgement and patient experience. The validation phase of this study is ongoing.

**Trial registration number:**

NCT04435041.

**Strengths and limitations of the study**

- First systematic study to develop items for a COVID-19 early warning score for primary care.
- Captures clinician and patient experience of the deteriorating patient with COVID-19.
- Combines extensive qualitative research and quantitative consensus methodology.
- Items have strong face validity, but formal validation of the score is still ongoing.
- REmote COVID-19 Assessment in Primary Care is a severity prediction score, not a diagnostic score.

## Introduction

Some patients with COVID-19 experience deterioration (usually at around day 8).^1 2^ There is therefore a need for research to develop accurate early warning scores—clinical prediction models designed to identify patients who need escalation to next level of care.^3^ Such scores need to be both highly specific (detecting all patients who need onward referral) and fairly sensitive (excluding all or most patients who do not). A recent systematic review of prediction models for COVID-19 concluded that ‘proposed models are poorly reported, at high risk of bias, and their reported performance is probably optimistic’ (p2).^4^ That review identified no evidence-based prediction models for primary care settings, nor did a citation-track of the article (which identified over 300 subsequent papers).

Assessment of a patient with suspected acute COVID-19 in primary care is fraught with uncertainty, since its clinical course differs from other pneumonias^5^ and because most patients will be assessed remotely (ie, by phone or video).^6^ Initially, the UK Royal College of General Practitioners cautiously endorsed the use of NEWS2 (National Early Warning Score 2) alongside clinical judgement for the assessment of patients with suspected acute COVID-19,^7^ but subsequently withdrew this recommendation. NEWS2 is calculated from the patient’s temperature, pulse rate, respiratory rate, systolic blood pressure, pulse oximetry reading and new confusion.^8^ The National Institute for Health and Care Excellence (NICE) rapid guideline on management of COVID-19 pneumonia in the community makes the guarded statement that the NEWS2 score ‘may be useful’ in assessing deterioration but that the patient should not be brought in for a face-to-face assessment solely to calculate a NEWS2 score (paragraph 3.7).^9^ But NEWS2 was developed for a different purpose (see Discussion section), and requires data that may be difficult to obtain. A recent preprint suggests that it correlates poorly with severity of COVID-19.^10^

We sought to develop and validate a primary care early warning score for COVID-19 based on data that can be reliably collected during a remote consultation. This paper describes the development of items for a version 0 of RECAP which can be formally validated. It does not cover the actual validation of the instrument.

## Method

### Study design and governance

**Figure 1:**
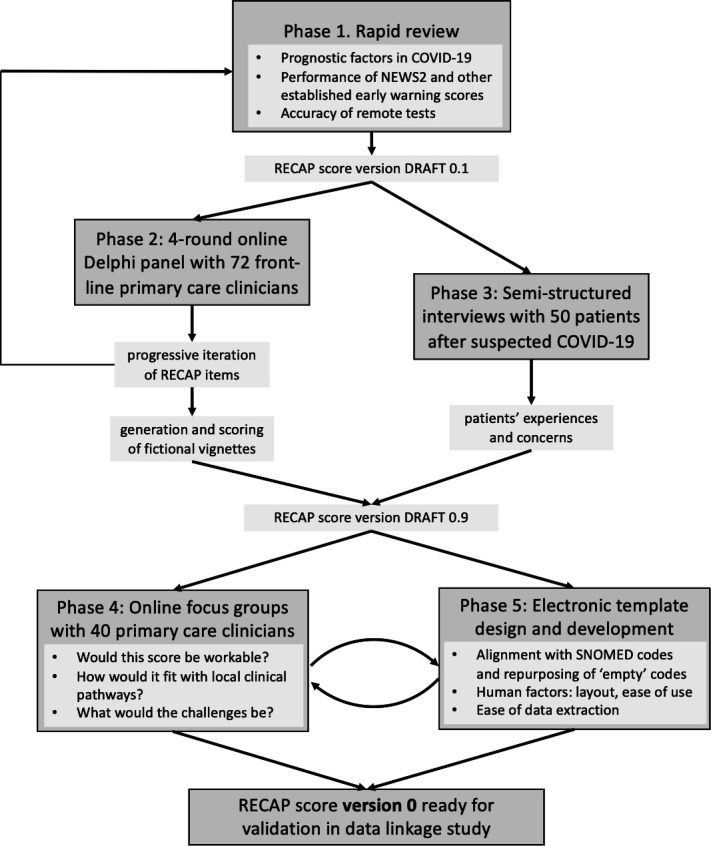
Study flowchart. NEWS2, national early warning score 2; RECAP, remote COVID-19 assessment in primary care.

The study was part of the Remote By Default research programme, funded by UK Research and Innovation COVID-19 Emergency Research Fund. It consisted of five phases (figure 1): rapid review, a four-round Delphi panel of primary care clinicians, semistructured interviews and focus groups with patients, focus groups with primary care clinicians and electronic template development. This in-depth mixed-method design was chosen because of the novelty of the condition, the high degree of clinical uncertainty surrounding its acute management and the added complexity of the need for remote assessment (which required judgements to be made without having fully examined the patient). For all these reasons, a detailed qualitative phase was considered essential before developing the score using actual outcome data and then undertaking a validation exercise. The study was overseen by an independent advisory group with a lay chair and a separate patient advisory group. Ethics approvals are included at the end of the paper.

### Rapid review

Detailed methods have been published elsewhere.^5^ Briefly, fortnightly keyword searches were conducted from March to June 2020 of PubMed for English-language systematic reviews and LitCovid and MedRxiv for reprints. Methodological quality of reviews was assessed using A MeaSurement Tool to Assess Systematic Reviews-2 (AMSTAR-2).^11^ Primary studies within included reviews were cross-checked for duplication before extracting data on each symptom or sign in both mild and severe COVID-19. The synthesis was continually updated over the course of the study period from the most up-to-date, highest-quality systematic reviews. To construct the items for the draft RECAP score, we selected symptoms and signs that were significantly commoner in severe than mild disease. The search was repeated in October 2020.

### Delphi panel

Delphi is a structured approach to working towards consensus.^12^ Steps include defining a problem, selecting a panel of experts (both academics and clinicians), supplying evidence along with key uncertainties, collecting quantitative (numerical scores) and qualitative (free text) data on a set of statements, feeding scores and comments back to panel members and repeating until residual disagreement cannot be resolved. Advantages of this method include practicality (it can be done online, asynchronously, without specialist tools), anonymity (participants know the average group score but not individuals’ scores) and iteration (feedback prompts outliers to either defend their response to the group or change it).^13 14^

Following established methodology,^12^ we used a social media announcement (TG’s Twitter account) and snowballing from people thus recruited to obtain 72 volunteers (68 general practitioners, three nurse practitioners and one paramedic), all of whom were actively involved in assessing acutely ill patients with suspected COVID-19. Ten of these were clinical academics. The ‘draft V.0.1’ RECAP items developed from rapid review was entered onto a questionnaire using Survey Monkey (homeworking during lockdown prevented us from accessing our usual professional survey software).

We sent brief instructions to participants, including a summary of what was known about prognostic factors in COVID-19. We gave them 5 days to complete the survey, scoring each item for relevance (should it be included?) and wording (how would they improve it?) and add free text comments. We sent two prompts to non-responders. A researcher (PT) imported the raw data into R and collated quantitative responses using simple descriptive statistics (percentage selecting, eg, yes as currently worded, yes but change wording, not sure, probably not and definitely not), and tabulated qualitative responses, which TG subsequently arranged under broad clinical categories and themes. We amended the draft RECAP items before circulating a summary of data, notes on changes and reasons for them and the next iteration of the items. This was repeated for second and third rounds.

The fourth round included only three residual items on which we had not yet achieved our goal of 80% consensus, plus a set of five fictional vignettes which incorporated various scenarios (such as missing data, untrustworthy technology, complex social circumstances and comorbidities) that participants had proposed in their free-text responses. They were asked to assign points to individual items and then calculate a simulated RECAP score for each of these five vignettes, and also indicate their level of clinical concern. This fourth round was repeated following a report from several participants that they had been unsure how to calculate the total (so had guessed); in the repeat round, we adjusted the software to add up the items automatically.

### Patient interviews

UK-based survivors of COVID-19 were recruited using three methods: a social media call (again, TG’s Twitter account), snowballing from those who responded (to access those not on social media) and an email sent from a support group for people with prolonged COVID-19 symptoms (LongCovidSOS).

AR conducted an initial round of 20 audio recorded interviews by telephone or MS Teams, taking verbal consent. Patients were asked to narrate their various contacts with healthcare services, including how symptoms were assessed by call handlers or clinicians. Relevant sections of interviews were transcribed and read independently by AR and TG. Early in the study, data were extracted into a word document and arranged into categories to correspond to the items in RECAP-V0, and any additional categories not included in those items. Later in the study, these data were combined with comments from 30 additional survivors of acute COVID-19 extracted from a larger dataset of interviews and focus groups. The data were formally coded in NVIVO software and checked against the final RECAP-V0 items to confirm that all relevant issues raised by patients had been captured.

### Clinician focus groups

Focus groups are a qualitative method designed to capture both individual perspectives and group dynamics (such as empathy, humour and conflict).^15 16^ There is an established methodology for conducting them by video-conference.^17^ A new sample of 40 primary care clinicians (28 general practitioners, 11 nurse practitioners, 1 paramedic) who regularly assess suspected COVID-19 patients was recruited by a social media invitation enhanced by snowballing. Focus groups were held by Zoom and lasted 90 min. Prior to the group, we circulated ground rules (eg, about confidentiality) and invited participants to read and score the five vignettes to familiarise themselves with RECAP-V0. Each focus group was facilitated by two academic general practitioners (TG, ALN or SW) guided by a list of prompt questions (reproduced in online supplemental file 1 on bmj.com). Briefly, we sought their views on RECAP-V0 (including a mock-up of the electronic template), and how they felt it would align with local clinical presentations, care pathways, electronic templates, resources and workflows. We asked what challenges they anticipated with its implementation.

10.1136/bmjopen-2020-042626.supp1 Supplementary data

Focus groups were video-recorded with consent. They were not fully transcribed but the clinical researchers all listened independently and transcribed selected sections. This free text was organised thematically on an Excel spreadsheet using the Framework method.^18^ Insights from focus groups were fed iteratively into the software design phase described below.

### Electronic template design and development

MD and BD matched individual RECAP-V0 items with existing Systematized Nomenclature of Medicine Clinical Terms (SNOMED-CT) codes where possible. A template was constructed using drop-down menus, and inserted as a supplement to the Ardens COVID-19 assessment template built for EMIS Web (Egton Medical Information Systems), and in the NW London Integrated Care COVID-19 templates. A further round of review of these codes was conducted with input from NW London CCGs (S-J Knight) and Dr Simon Gordon.

## Results

### Rapid review

Five factors appeared to predict poor outcome in COVID-19: persistent fever, shortness of breath, low oxygen saturation, muscle aches and certain comorbidities.^5^ Patients with severe COVID-19 may develop shock (manifest, eg, as deteriorating conscious level, hypotension and reduced urine output). Based on these findings, we constructed a preliminary list of 13 items. Later in the study, additional questions emerged which prompted new rapid reviews: assessment of breathlessness,^19 20^ how to measure exertional desaturation accurately and safely in patients with suspected COVID-19,^21^ and the reliability of smartphone oximeters^22^ and smartphone blood pressure measuring apps.^23^

### Delphi panel

The Delphi panel was conducted in April and May 2020. Round 1 had a sample of 69 clinicians; three more joined for rounds 2–4. Of these, 62, 53, 56 and 51 completed the surveys, respectively—response rates of 90%, 74%, 78% and 71%. Progression towards quantitative consensus across the four rounds is shown in table 1.

**Table 1:** Results of Delphi panel

Focus of item	Percentage agreeing with item as worded			
	Round 1	Round 2	Round 3	Round 4
	(n=62)	(n=53)	(n=56)	(n=51)
Pulse rate (wording of item)	73	70	88	
Cut-off values for each category	71	77	84	
Temperature (wording)	58	75	89	
Cut-off values	55	81	89	
Duration of temperature* (wording)	58			63
Cut-off values				76
Symptoms of fever (eg, chills, shivers)	29	75	91	
Cut-off values	63	87	95	
Respiratory rate (wording)	61	85	84	
Cut-off values	76	85	91	
Shortness of breath (wording)	39	75	79	
Cut-off values	44	79	93	
Trajectory of breathlessness				73
Cut-off values				67
Oxygen saturation level	65	79	80	
Cut-off values	65	85	82	
Oxygen saturation level after exercise	–	68	75	
Cut-off values		85	91	
Clinical suspicion of hypoxia	47			
Cut-off values	55			
Tiredness (wording)	42	83	88	
Cut-off values	73	89	84	
Muscle pains or aches	66	–	64	82
Cut-off values	47	–	79	
Risk factors (comorbidities)	53	57	86	
Cut-off values			82	
Risk factors (demographic, eg, age, ethnicity)			77	
			86	
Indicators of shock (including conscious level, new confusion†, low or no urine output, cold and clammy, mottled skin)	61	64	Moved to ‘red flag’ box to align with national guidance	–
Other red flag symptoms, for example, central chest pain	73	34		
Clinical concern	74	77	86	

*‘Duration of temperature’ was changed to ‘duration of symptoms’ after focus group discussion, with 8 days seen as the cut-off for clinical concern.

†New confusion was subsequently proposed as a separate RECAP item by focus group participants, who did not agree that it should be an automatic ‘red flag’ sign.

RECAP, remote COVID-19 assessment in primary care.

In summary, at least 80% agreement on relevance, wording and value sets for severity was eventually achieved for eight items: pulse, temperature, symptoms of fever (for use particularly if the patient does not have a reliable thermometer), respiratory rate, oxygen saturation level, tiredness (which, if severe, may indicate hypoxia and hence potentially substitute for an oximeter reading), muscle aches and known risk factors (comorbidities). Additional items with moderate agreement included demographic risk factors (77% agreement), oxygen saturation level after a 40-step exercise test (75%), trajectory of breathlessness (73%) and duration of temperature (63%).

Our qualitative data set from the Delphi panel included over 200 pages of comments. Key themes are summarised and illustrated in online supplemental file 2 on bmj.com. These, along with rapid review, allowed us to characterise the clinical features of the deteriorating COVID-19 patient in primary care (box 1).

10.1136/bmjopen-2020-042626.supp2 Supplementary data

Box 1 **The clinical course of the deteriorating COVID-19 patient in primary care**

**Synthesised from our qualitative data, supplemented from published sources**

COVID-19 may present in primary care as a viral upper respiratory tract infection (eg, sore throat), lower respiratory tract infection (eg, cough, fever and mild dyspnoea), influenza-like illness (with fever, chills, headache and myalgia) or gastrointestinal illness (with abdominal pains, nausea and diarrhoea).^2 6^ Most patients have a relatively mild, self-limiting illness, but an unknown proportion (perhaps 10%) deteriorate, usually in week 2. Certain symptoms common in week 1, such as cough, mild fatigue and anosmia, do not appear to have prognostic significance.^5 44^

It is important to date-stamp the onset of first symptoms.^6^ Severe dyspnoea, especially at rest, may indicate progression of lung involvement. The trajectory of dyspnoea is important, as acute respiratory distress syndrome occasionally follows quickly from the onset of breathlessness.^45^ Formal scores for assessing dyspnoea severity appear to have a significant false negative rate and should not be used.^20^ A careful history, noting what the patient is able to do and what they cannot do today that they could do yesterday, is likely to be more important.^20^ A patient’s or carer’s concern about the severity of breathlessness may be significant and should not be dismissed as ‘anxiety’.

Pulse oximeter readings are extremely useful in assessing unwell patients with COVID-19, so long as the device is reliable (smartphone apps are inherently inaccurate and should not be used)^22^ and the patient or a relative is capable and confident to use it. The finger must be warm. While a low oximeter reading is concerning, a normal one should not necessarily reassure, as young fit patients in particular can compensate well in the early stages of deterioration.

So-called silent hypoxia, defined as the development of respiratory failure without the subjective perception of dyspnoea, is a recently described feature of severe COVID-19 and appears to have a poor prognosis.^44 46–49^ Anecdotal accounts suggest that in some patients, silent hypoxia may manifest as profound tiredness, but we could not find published research on this association. New confusion (especially in older patients with comorbidity) was considered by clinicians in our sample to be a poor prognostic sign in COVID-19, but at the time of writing, evidence for this is limited.^50^

COVID-19 lung damage tends to be manifest as a perfusion defect (ie, difficulty transferring oxygen across the alveolar membrane) rather than a ventilatory defect (difficulty getting air to the alveoli, as in asthma).^45^ This may explain why COVID-19 can behave similarly to pneumocystis pneumonia in producing a fall in pulse oximetry reading on exertion (or in the minutes following exertion).^21 51^ Because of this, patients with suspected COVID-19 should not be subject to exercise testing unless there is a clinician present if their resting pulse oximetry reading is abnormal (below 96%).

An unwell patient may or may not have COVID-19. An overall assessment is needed using questions relating to (eg) hydration status, dizziness, falls, central chest pain, fall in blood pressure (if the patient has equipment at home), change in mental status (including lethargy, new confusion, difficulty in rousing), central cyanosis (eg, blue lips) and severe reduction in urine output. For this reason, a standard ‘red flag’ checklist should be quickly reviewed in all unwell patients.

There are some well-established risk factors for developing COVID-19 and worse outcome (eg, age, non-white ethnicity, high body mass index and comorbidities including cardiovascular disease, hypertension and active cancer).^52–54^ The extent to which these risk factors should be applied to ‘load the score’ of a patient who appears to have a mild form of the disease is not yet known, especially since shielded patients are the ones for whom a hospital or clinic visit carries most risk.

In addition to prompting new rapid reviews, the Delphi qualitative data shaped the development of the items in several ways. Particular forms of words (eg, to question patients about the severity and rate of deterioration of breathlessness) enabled us to refine our items and value sets. Participants alerted us to existing guidance and protocols used either nationally or locally (there was strong consensus that any new instrument should complement rather than replace these). Comments about missing or untrustworthy data when a patient was being assessed at home via telephone or video link prompted us to develop default value sets or alternative questions to compensate for such deficiencies. The free-text comments included rich data, based on real clinical experiences, from which we were able to construct the vignettes used in discussions. Numerous comments on the practicalities of applying the potential instrument prompted us to set up focus groups to explore these operational challenges further.

The five fictional vignettes and the results of the simulated scoring exercise on these are reproduced in online supplemental file 3 on bmj.com. In summary, while there was considerable variation in the number of points given (eg, in whether clinicians judged an ill-defined set of symptoms as ‘moderately’ or ‘severely’ tired), in all five vignettes, the simulated RECAP-V0 score as calculated appeared to prompt an appropriate and cautious response. For example, in a case of an elderly South Asian patient who spoke no English and with no equipment at home for the family to take measurements, all but one participant were prompted by the RECAP-V0 simulated result to assess the patient in a face-to-face encounter. In a case of an African Caribbean man with profound tiredness and rapidly worsening breathlessness in the second week of his illness, all participants were prompted to arrange urgent transfer to hospital. Free-text comments in several vignettes indicated that some respondents’ level of clinical concern had not been especially high and that they were surprised that the RECAP-V0 simulated score was so high.

10.1136/bmjopen-2020-042626.supp3 Supplementary data

### Patient interviews

Of the 20 patient interviews in our original sample, 15 included detailed descriptions of deteriorating symptoms in the acute phase of COVID-19. These included worsening breathlessness, inability to speak in sentences, profound fatigue without feeling short of breath, high temperature and symptoms interpreted as anxiety but which may have been acute tachycardia. Patients also described being reassured by remote assessments using video examination and home monitoring equipment.

… when I was speaking to them they were listening to me breathing and watching my chest and things. And they were seeing my breathing through the video link, wanting to see a clear vision of my chest area and counting my respiratory rate. And looking back when I spoke to them I was breathless. […] I’ve already got a pulse oximeter and a blood pressure machine. […] The pulse ox(imeter) was really good. What I had to do is when they video called I had to put it on my finger and wave it at the screen and they were able to monitor it. (patient NM1)

Some patients described long waits to get through to NHS 111 (the English telephone triage service), being asked ‘tick box’ questions by call handlers or clinicians, and feeling dismissed on the basis of such questions even when they were concerned about the extent and pace of their own (or a relative’s) deterioration. For example:

You can’t make a diagnosis on the phone because you don’t get to see the physical symptoms. They (clinicians) never saw the (patient’s) rash, I described it but they didn’t actually get to see it. They couldn’t see she was sweating all around her hairline and her face was super pale. The overall picture they couldn’t see—they just had written down some numbers. (relative of patient, HG1)

The ‘exercise‘ being referred to in the quote below is probably the Roth score,^24^ which is likely neither sensitive nor specific in the assessment of COVID-19 patients.^20^

There was an exercise where I need to count to something a certain number of times. They did a small exercise. But now they know you can have severe hypoxia without clear shortness of breath when talking. That was the assessment. They said ‘I know it’s rough, soldier through. You’re 34 years old. There’s no point in escalating this.’ (patient SN1)

These patient data helped us refine the wording of the questions on the RECAP-V0 items and also the clinical description in box 1. In particular, the patient data emphasised the importance of developing the prediction instrument as an adjunct to expert clinical judgement and not as a substitute for it. Additional qualitative data from 30 further COVID-19 survivors confirmed these themes and identified one additional relevant finding: that mismatches between the clinician’s and patient’s assessment of severity occurred, and were sometimes attributed by the clinician to anxiety:

He (call handler who was being advised by nurse) said you seem to be able to talk in complete sentences. I was concerned because I thought I couldn’t. I said I’m trying my best but I am struggling to talk, you might not hear it but it’s definitely been happening this evening. (…). And the nurse asked the call handler to ask me if I suffer from anxiety.

### Focus groups

In focus groups, clinicians described COVID-19 as a disease with ‘surprises’. They were especially concerned with patients whose initial course was unremarkable but who subsequently deteriorated rapidly. They wanted an early warning score not primarily to identify ‘red flag’ patients (whose need for urgent hospital referral they felt was usually obvious), but for assessing moderately sick patients and tracking their progress over time. They considered the RECAP items to have high face validity and to reflect their level of clinical concern on the vignettes. Some felt that RECAP would be less useful for assessing an unwell patient who did not have COVID-19 (though this may be unfounded - see Discussion). All three groups proposed the same additional item (patient less alert than usual or new confusion), and there was also agreement on a minor change to one item (changing ‘duration of temperature’ to ‘duration of symptoms’). Participants made suggestions for layout and ordering of items on the electronic template, emphasising simplicity, ease of use and ‘human factors’.

### Developing the RECAP items

**Figure 2:**
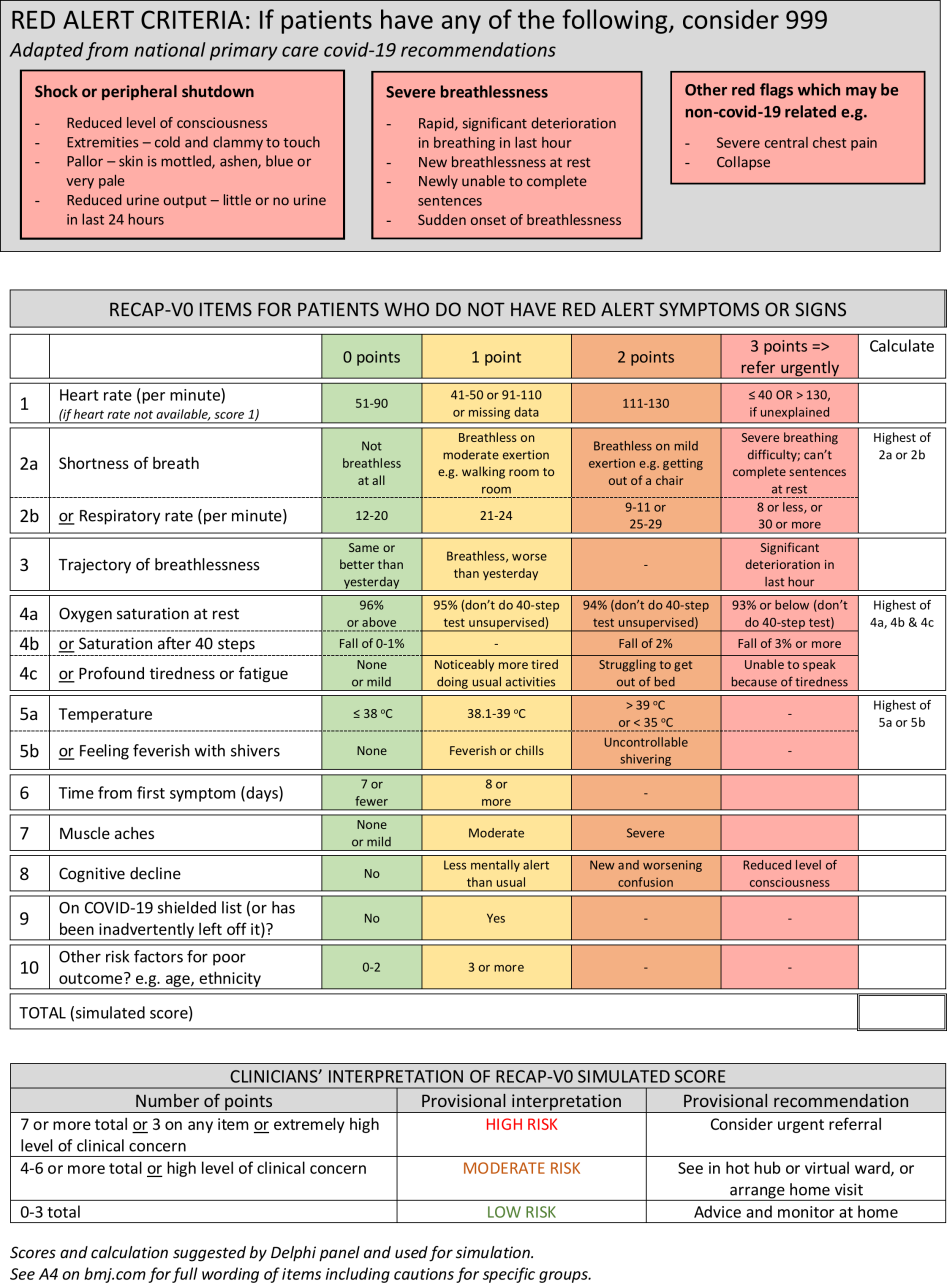
Summary of RECAP-V0 items. RECAP, remote COVID-19 assessment in primary care.

Data from the rapid reviews, Delphi panel, patient interviews and clinician focus groups contributed to development and refinement of the items for RECAP-V0 (figure 2) which will go forward for further development (to create a final set of items and scoring weights) and validation. RECAP-V0, which cannot yet be called a ‘score’, consists of 10 items, three of which include alternative options designed to enable assessment even in the absence of technologies (video camera, thermometer and oximeter). Further explanatory text is given in online supplemental file 4 on bmj.com.

10.1136/bmjopen-2020-042626.supp4 Supplementary data

### Electronic template design and development

To make an electronic version of RECAP, we selected precoordinated terms that were uniformly present in EMIS and SystmOne EHR systems, so that the same terms could be used and exported for linkage nationwide. The template included a prompt to capture the patient’s verbal consent (supported by a URL for the information sheet) for data linkage (as the relevant SNOMED code bound to our unique NHS Clinical Research Network Portfolio Number), as well as data previously entered and saved as part of the standard COVID-19 assessment. Layout and ordering of items was adapted in response to focus group comments and insights. It proved impossible to identify suitable codes for a value set on severity of myalgia, so the final template supports only presence/absence of myalgia.

## Discussion

### Statement of principal findings

This mixed-method study has produced five key findings. First, we have developed, through consensus, a rich description of clinical deterioration in the primary care patient with COVID-19 (box 1). Second, we have achieved very high agreement among 72 front-line primary care clinicians and academics for the inclusion of a particular wording on the following variables for inclusion in an early warning score: pulse rate (88% agreement), temperature (89%), fever symptoms (91%), respiratory rate (84%), shortness of breath (79%), pulse oximeter reading (80%), postexercise fall in pulse oximeter reading (75%), tiredness (88%), muscle aches (82%), shielded list (86%) and other risk factors for poor outcome (77%), along with affirmation of these by a second sample of 40 clinicians, who added duration of symptoms and new confusion. Third, we have obtained a high level of agreement on the numerical or descriptive value sets for different signs and symptoms in each item (details in table 1). Fourth, we have confirmed that the items reflect the concerns of patients. Finally, we have surfaced, and begun to address, some of the human factors and operational challenges of implementing RECAP-V0 in different primary care services and settings across the UK.

### The study in context

This is the first study systematically to capture the clinical experience and wisdom of front-line primary care practitioners, as well as the experience of patients, in relation to the assessment of the deteriorating COVID-19 patient who has not yet been referred to hospital. Early Chinese studies on COVID-19 included only hospital patients.^1 25^ Other community-based studies identified symptoms (notably loss of smell) associated with COVID-19 but which lack prognostic significance.^26^

Our study was undertaken at speed, in the midst of the first wave of the pandemic, and was influenced by the practicalities of the UK National Health Service under unprecedented stress. Despite these pressures, we believe we have achieved a sufficiently large and diverse sample of front-line practitioners and patients to be confident that the findings reflect current best practice and patient priorities.

The main limitation is that we have not yet validated the instrument (see ‘Next steps’ below). Another limitation is that one item relies partly on UK-specific data (specifically the ‘shielded’ category). This item is, however, readily adaptable to reflect categories of vulnerability or risk used in other countries. Because the validation phase is not yet complete, we do not yet have weightings assigned to different items and selection of cut-off levels.

### Comparison with previous literature

To date, most early warning scores have been developed for use in hospital inpatients using routinely collected vital sign data.^27^ In UK hospitals, the NEWS2 score has become a common language of sickness with positive implications for patient safety (especially in relation to sepsis).^28^ NEWS2 is recommended by NICE guidelines both in general^29^ and as a component of the critical care of COVID-19 patients,^30^ though it has been heavily criticised even in hospital settings.^27 31–34^ NEWS2 has been used in prehospital settings by ambulance crews^31^ and in early detection of suspected sepsis in primary care.^35^ However, it has not been formally validated in general practice,^36^ so its sensitivity and specificity in that context are unknown. Its positive predictive value is low even in hospital and ambulance settings, and is likely to be even worse in primary care because serious illness is rare.^37^ A rise in NEWS2 appears to be a relatively late indicator of deterioration, typically triggering only in the last 12 hours before transfer to critical care.^27^ For all these reasons, NEWS2 might cause harm from both under-referral and over-referral in a primary care setting, though there is preliminary evidence that it may have some value for COVID-19 in care home residents, who tend to be sicker.^38^

The key differences between NEWS2 and RECAP-V0 are as follows. While NEWS2 was designed to be calculated from observations taken in hospital by trained staff and is based solely on signs, RECAP is designed to be completed in primary care as part of a clinician-patient conversation along with a (limited) remote physical examination, and is based on both symptoms and signs. With the exception of pulse rate (which defaults to a value of 1 if no reading is possible), all items requiring a physical examination include an alternative item based solely on symptoms. This will allow the clinician to populate the score even when the assessment is being done remotely and the patient is unable or unwilling to use equipment, thereby reducing the danger of missing data.^27^

While we set out to develop the RECAP score as a disease-specific instrument, a reviewer of a previous draft of this paper suggested that it may prove useful in both COVID-19 and non-COVID-19 acute deterioration since (he hypothesised) most symptoms of acute deterioration are not disease-specific. He drew our attention to a new study from Uganda which identified 12 ‘high-risk chief complaints’ in a prehospital population which were associated with increased acute mortality in the subsequent days.^39^ Interestingly, one of these was ‘difficulty speaking’ which had not previously been included as a red flag symptom or prognostic marker but which was prominent in our qualitative data.

Two recent publications describe the development and validation of an in-hospital severity score for suspected COVID-19.^40 41^ The International Severe Acute Respiratory and emerging Infections Consortium Coronavirus Clinical Characterisation Consortium study included over 35 000 patients in the derivation data set and over 22 000 in the validation dataset. The final 4C Mortality Score included eight variables, six of which (respiratory rate, conscious level, peripheral oxygen saturation, comorbidities, gender and age) overlap with items in RECAP-V0. The other two (urea and C-reactive protein) require a blood test. Unlike the 4C Mortality Score, RECAP-V0 includes several items based on (or substitutable with) the patient’s subjective symptoms including shortness of breath and muscle aches, both of which have been shown to correlate strongly with disease severity.^5^ RECAP-V0 also includes heart rate, as well as items based on time course (persisting fever on day 8 and trajectory of breathlessness), which reflect the clinical judgement of our Delphi panel of clinicians. We do not yet know whether any or all of these additional items improves the predictive value of the RECAP score.

### Next steps

The transparent reporting of a multivariable prediction model for individual prognosis or diagnosis (TRIPOD) statement^42^ states that development of a prognostic model (of which an early warning score is one example) requires two phases: instrument development and instrument validation. The study reported here has ensured that the first component of instrument development has captured three important dimensions: the existing evidence base from the research literature; the experience and intuition of front-line primary care clinicians and the experience of patients. The second component of instrument development is to collect and analyse data on these important dimensions of clinical observation.

In the next phase of this study (ongoing), we are completing development and validating the RECAP score using data linkage between general practice electronic records and national data sets (local data from North West London’s iCare and nationally via the RCGP Research and Surveillance Centre) using the primary outcome of hospital admission and secondary outcomes of intensive care unit admission and death. Recruitment into that phase of the study has begun. Further details are obtainable from the RECAP study website.^43^

Because of the novelty of the disease and the urgency of the research question, we decided to place our interim findings in the public domain so as to allow other teams to test and improve the RECAP items in parallel with our own continuing research (rather than, as is more commonly the case, seeking to ensure that our own validation study is published first). We welcome offers of collaboration from established research teams.

Until the findings of the next phase of the study are published, the validity of this instrument is unknown. Even if RECAP proves sensitive and specific for identifying the need for urgent escalation of care, it should be noted that this instrument is a severity prediction tool, not a diagnostic tool. It does not include items which are highly specific for diagnosing COVID-19 but are not predictive of its severity (eg, loss of smell), and it includes many items (such as standard red flag indicators) which are not specific for COVID-19.

## Supplementary Material

Reviewer comments

Author's manuscript
