# Supplementary material for: What items should be included in an early warning score for remote assessment of suspected COVID-19? qualitative and Delphi study
Source: BMJ Open. 2020 Nov 12;10(11):e042626. doi: 10.1136/bmjopen-2020-042626 (PMC7662139; doi:10.1136/bmjopen-2020-042626)
Supplement: Supplementary data [file bmjopen-2020-042626supp001.pdf]

Additional File 1: Focus group prompt questions

1. How has your **experience managing COVID-19** been? **How do you make decisions about who to refer on**?
2. Do you **use any clinical decision score**? If so, which one?
3. What would a warning score **need to do for you**?
4. **Which patients does it need to select** for you?
5. How was your overall **experience using RECAP**?
6. Which were the main **positive aspects of RECAP**?
7. Did you have **any difficulties using RECAP**? Which?
    a. In particular, how does clinical uncertainty (i.e. difficulties assessing objective measures, such as HR) impact the application of RECAP?
    b. How do remote communication barriers (i.e. language, no video, poor phone line) impact the application of RECAP?
    c. Was time a problem? (i.e. Is it short enough to be useful in clinical practice?)
8. **How do you think these problems/difficulties could be eliminated or overcome**? Can you anticipate any possible solutions?
9. How do your **local and structural arrangements** influence your ability to apply RECAP?
10. Do you feel you need any additional information or training on how to use RECAP?
11. Is there anything else you want to add or feel we should have spoken about?
12. Demonstration of RECAP template using screen share.

***

Other possible questions to be discussed:

Are there any other ways to achieve #3?

Does RECAP seem capable to select those who would, or not benefit from oxygen therapy or heparin?
