# Supplementary material for: What items should be included in an early warning score for remote assessment of suspected COVID-19? qualitative and Delphi study
Source: BMJ Open. 2020 Nov 12;10(11):e042626. doi: 10.1136/bmjopen-2020-042626 (PMC7662139; doi:10.1136/bmjopen-2020-042626)
Supplement: Supplementary data [file bmjopen-2020-042626supp002.pdf]

# Additional File 2: Qualitative comments during Delphi process

## Pulse rate

*"We have noted that about 50% of our patients have BP monitors at home, which has been very helpful in our video consultations. In addition, we send out sats bus out to those patients we are concerned about, so have been able to include the PR [pulse rate] even in patients without." [round 1]*

*"I think it [pulse rate] is really helpful in identifying sick young fit people who are otherwise compensating quite well and may have normal resting sats and a slightly high RR [respiratory rate] and significant fatigue and look ok. Less helpful in older adults unless very high. I have no idea of its predictive value for outcomes though." [round 1]*

*"We have seen a lot of tachycardia in patients with milder symptoms so I think 2 rather than 3 is an appropriate score" [round 2]*

*"A bradycardia below 40 is an emergency whether or not a patient is on a B-blocker so the advice to adjust by 10 should not apply. Just because a patient uses a B blocker doesn't mean they can tolerate a low cardiac output when septic/unwell." [round 3]*

*"They [pulse rate cut-offs] don't align with London guidance"*

## Temperature and symptoms of fever (chills, shivers)

*"In my (pre-Covid) experience, patients are excellent at recognising rigors as being different from a normal high temperature." [round 1]*

*"Temp 37.8 by NHS 111 is I think more about meeting clinical criteria for isolation, rather than because of any clinical utility. I think your cut offs are sensible." [round 2]*

*"'use peak temperature before paracetamol' implies a level of control that the clinician won't have. Why not 'highest recorded temperature in the last 24 hours' or similar?" [round 3]*

*"I would just score 1 for a temperature, rather than the height of the temperature" [round 3]*

*"If you mean 'rigors' then the orange box should have the word 'rigors' in it. Shivers and chills aren't rigors (I've had both). 'Feverish or chills with uncontrollable shivering' perhaps is closer to the implied meaning." [round 3]*

*"I suspect that chills is a greater reflection of malaise and temperature than the height of pyrexia and therefore reflects greater illness (but I'm guessing!)." [round 3]*

## Respiratory rate

*"We have found RR [respiratory rate] as a very helpful tool with our video consultations and are using it to determine which patients require home visit/sats bus/hub attendance, with the cut off of more than 24 and less than 9. We are requiring RR for all referrals to our hot hub." [round 1]*

*"Below 9 to score two- no way! That's a 999 emergency. I have MRCEM and was an intensivist for several years. A low resp rate scares me a lot more than a high one. A low RR tells you the patient is peri-arrest. A high RR is worrying but tells you that the patient is - for the moment - responding appropriately to their respiratory or metabolic problem. A low RR tells you that they are no longer able to do so and are about to die without*

*intervention (some exceptions, of course, e.g. BZD plus alcohol OD when people often maintain a scarily low RR without apparent significant compromise - but not relevant here)." [round 2]*

*"I would amend to 2 = 25-29 and score 3 if greater than or equal to 30, to align with CURB and other scores. A RR of 30 is pretty rare and severe." [round 2]*

*"Almost certainly a good sign but worried about its feasibility" [round 3]*

## Breathlessness and silent hypoxia

*"We have seen many patients with silent hypoxia. These patients have tended to be extremely fatigued. Would you consider including a normal RR but with extreme fatigue as a score 3?" [round 1]*

*"In practice we have noticed that patients are not articulating their breathlessness well - asking them about how their symptoms (both breathlessness and fatigue) are affecting their usual activities has been key - struggling to get out of bed, for example, is often a very significant change, for others it's not being able to manage the hoovering, for all it's changes to their usual activities and routine" [round 1]*

*"Would be good to have something to try and capture the silent hypoxics - such as feeling more fatigued and exhausted on exertion? Which I have had patients describe? Or a feeling that breathing is laboured but not breathless. Like at altitude." [round 1]*

*"Persistent, progressive SOB is a red flag" [round 1]*

*"Listening to friends (sadly several) who have suffered with COVID and even been to ICU, lot of them were that tired that they could not even speak." [round 2]*

*"Maybe 2 and 3 are too close together? How about 0: as it is 1: new breathless on moderate exertion eg up stairs 2: Breathless on mild exertion eg walking across room. 3: unable to complete sentences or severe difficultly breathing" [round 2]*

*"Struggling to get out of bed might not be that unusual for some people!" [round 2]*

## Illness trajectory

*"The patients I've been worried about tend to be pyrexial >38 in week 2" [round 1]*

*"From the various narratives on the illness I wonder about days since symptoms began - it seems that patients in the first 7 days of symptoms may be less likely to deteriorate, whilst those whose symptoms go on after this probably need assessment. Careful assessment of confusion feels important - for my phone calls I am spending time talking to patients to assess for any level of confusion. This for me is a 'sign of hypoxia' - but that is not flagged clearly enough in the above for my liking." [round 1]*

*"[Tiredness] varies so much on stage of illness. Those with mild symptoms all c/o feeling very tired in the first 5 days, often not able to get out of bed for a day or two. But can manage their ADL [activities of daily living]. Worsening fatigue around day 7-10, having initially been feeling better, is a much worse prognostic feature in my limited experience." [round 1]*

## Pulse oximetry and exertional desaturation

*"I think the walk test is really important." [round 1]*

*"Validated scoring system already in place I ask patients to walk 40 steps and then check by video with hand in their chest easier to see and count and sees how they are on exertion in comparison to rest ." [round 1]*

*"Until we know more I'm not sure that we can leave patients with pre-existing normal lungs at home with a score of 93%- this may be the best early warning that we can find." [round 2]*

*"Always a problem what to do with chronic lung disease. 6% [correction for chronic lung disease] is quite steep (since obviously depends on degree of chronic hypoxia) but I don't have any more rational suggestion." [round 2]*

*"NB: pan-London respiratory guidance decided not to specify a number for desaturations in latest draft. I had to miss that meeting so not sure about the reasoning, but I think it was something along the lines of needing to use clinical judgement. Personally, I think 'clinical judgement' needs some guidance as this is a new disease entity, so strongly support having specific numbers on this to guide clinicians." [round 2]*

## Muscle aches

*"This is interesting. I just have not spoken to or seen many people who complain of severe muscle pains at all. This might be because I am seeing people (in the community as a GP) who have mild to moderate disease, and those with really severe disease with severe myalgia bypass their GPs and go straight to hospital. If hospital colleagues are saying it does seem to predict severity then leave it in." [round 4]*

"*muscle pain has been very common in our unwell patients" [round 4]*

## Red flag signs

*"Central cyanosis needs 999." [round 1; 30 similar comments]*

## Comorbidities and demographic risk factors

*"3 for 61yo male with BMI of 32 is not equal to active cancer, immunosuppressed and high fever in week 2?" [round 2]*

*"If the other questions don't raise concerns then i don't want to send thousands of obese diabetic hypertensives with mild disease for assessment - I think they need to be given specific advice that they are at risk of becoming more unwell quickly - so please call back if any concerns" [round 2]*

*"I think the cut offs are perfectly rational, but the comorbidities are complicated and hypertension in particular." [round 2]*

*"All patients on shielded list and having 3 risk factors will score 4 meeting the criteria for F2F assessment even if they have no concerning clinical features. This means that any non-white male patient over 65 and on the shielding list needs a F2F assessment before we even start with the clinical assessment, which is not appropriate. So I think the scoring levels need to be reconsidered and reduced for this or 6b. My inclination would be to reduce this to a 1 if on list and leave 6b as it is." [round 3]*

*"I would have the top score as '4 or more'. A fit 68yr old Asian male with no other illnesses, or a white male with either well controlled hypertension or diabetes scoring 2 feels a little high." [round 3]*

## Other comments

*"I don't trust China data" [round 1]*

*"I think you have covered the important things. The key here is to assess physiology. I think history is less important. The patients who become very unwell have a large systemic inflammatory response or severe pneumonia/pneumonitis so we need to focus on identify when people have SIRS or significant respiratory compromise." [round 1]*

*"I am very concerned about the application of clinical scores to patients that don't capture the individuality of patient circumstances - particularly if they are to be used to inform important decisions such as admission to hospital or deciding that a patient is in need of end of life care. There is always a danger that scores will be used to reduce and resolve these hugely complex issues - and are very clinician dependent." [round 1]*

*"I am not clear on the aim of the scoring system, and whether it acts as 'protocol' rather than a decision augmenting tool.. I hope you don't mind my honesty as I would like to help produce a simple, user-friendly diagnostic and prognostic tool.. but I think it needs to augment existing high level clinical skills rather than be another protocol to fill, creating over-reliance on this tool and cognitive distraction in the field. " [round 2]*

*"a really important part of our assessment in primary care is social circumstance and / or ability to mobilise community services. Lack of either of these can lead to admission too - while they may not be clinical "red flags" they may be worth including somehow as they contribute to the rationale for the decision to admit to hospital (and capture a little about the person being admitted)" [round 2]*

*"An observation. I realise that it is an unavoidable consequence of remote assessment but if you have a device to measure sats or pulse rate, you have 'more chance' of having a higher overall score." [round 3]*

*"There are too many questions if all included, so it is too easy to get a high score - need to reduce the number - could aim for 8 questions as in NEWS2? Would it be worth considering a table top exercise before sending out for initial testing? (eg give some scenarios & decide if admit or not & then do score to see if confirms decision or not?)." [round 3]*
