# Supplementary material for: What items should be included in an early warning score for remote assessment of suspected COVID-19? qualitative and Delphi study
Source: BMJ Open. 2020 Nov 12;10(11):e042626. doi: 10.1136/bmjopen-2020-042626 (PMC7662139; doi:10.1136/bmjopen-2020-042626)
Supplement: Supplementary data [file bmjopen-2020-042626supp003.pdf]

Additional File 3: Summary of vignette study

We used five vignettes, deliberately presented as 'grey cases' with considerable clinical, technical and social uncertainty (missing data, vague symptoms, potentially irrelevant material). In the first round of vignettes, we asked participants to score each item on the RECAP instrument, calculate the RECAP score and then say what they would recommend for the patient and whether the score reflected their clinical concern.

The 51 responses to this round showed surprising variation (e.g. the RECAP score varied from 2 to 16 in one vignette). However, free text comments suggested that the respondents were unsure how to calculate the score from the individual items (uncertainty which to add with 'and' and which to use as alternates with 'or', and whether to count the clinical concern within the RECAP score or separately from it).

We therefore ran a second round of vignettes (using three of the five), and using an automated scoring system. This produced 37 responses and a more consistent response, though still a fairly wide spread. Free text responses suggested that respondents found the vignettes time-consuming and somewhat confusing.

Details analysis is below.

VIGNETTE 1: Mrs Rahman

> *Mrs Rahman (South Asian ethnicity) is 78; she lives with her extended family who are concerned about her. She has had a dry cough and has felt hot for 6 days. Her appetite is poor (she has apparently lost her sense of taste). She spends most of her time in bed and shows no signs of getting better. When you try speak to her, you have no common language but her daughter-in-law says she is not short of breath, just very tired. She says her mother-in-law gets up to go to the toilet, and comes downstairs once a day to watch a TV programme. She says she doesn't look blue or pale. She's been hot and sweaty but has not had uncontrollable shivering, but she is aching quite badly in her back and down her legs, and is very worried. Mrs Rahman has well-controlled type 2 diabetes (on metformin). Her BMI is 27 and a recent BP was 130/85. She had stage 2 breast cancer 20 years ago but no recurrence. The family do not have a thermometer, blood pressure machine, oximeter or other equipment, and nobody has access to a video application.*

Scoring in round 1 (51 responses):

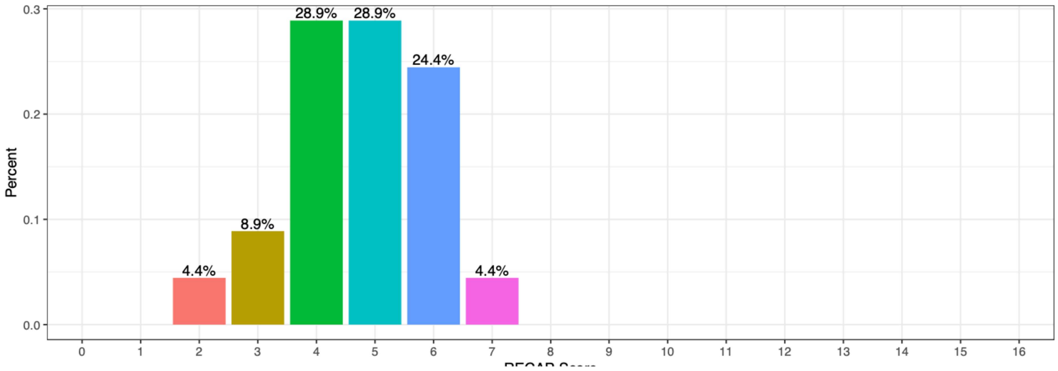

Scoring in round 2 (37 responses)

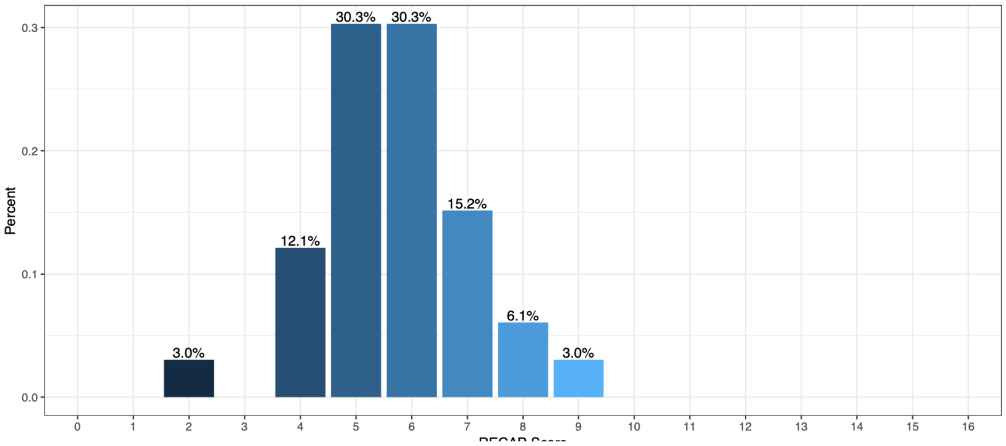

In sum, 82% of the first sample and 72% of the second sample would see in hot hub. 13% of first sample and 3% of second sample would reassure. Interestingly, 4% of the first sample and 24% of second sample would refer to hospital.

Responses to individual items showed that the main ones accounting for variation were

- Tiredness: Noticeably more tired doing usual activities (70%) versus struggling to get out of bed (30%)
- Fever: None (10%), feverish or chills (87%), feverish or chills with uncontrolled shivering (3%)
- Duration of temperature: not applicable (9%), fewer than 7 days (91%)
- Muscle aches: none (3%), moderate (61%), severe (36%)
- Is patient on shielded list? Yes 6%, no 94%.
- Risk factors for poor outcome? No 9%, yes 91%.
- Clinical concern: low 3%, moderate 70%, high 27%.
- Did RECAP reflect clinical concern? Yes 90% No 10%
- What would you do? Reassure 3%, see in hot hub 91%, refer urgently 6%

Qualitative comments:

Many said can't remember what the score is.

"It effectively has a bunch of 'not assessed' elements though, so the risk is that they cannot contribute to the score because missing, but missing does not equal 'not present' if there is a communication barrier (language but also no video or a poor phone line, or an inarticulate person who struggles to explain what they feel etc). I think it delivers the 'right' outcome though in the sense of face to face community review."

"Not clear how to score if no HR available Q4b) states feverish with shivers but then the score says "feverish or chills" scores 1 point... Q6b) not clear if I would have counted controlled hypertension as one factor, and also if I would use the BP cut off for diabetes in giving a point for diastolic >80... Scored 6 and then gave additional points for moderate worry = 8, which would result in urgent referral but I think I would have seen this patient in a hot site rather than admitting."

"Seeing her in the Red COVID Assessment Unit would feel right. Then we could assess parameters and see what her obs are."

Conclusion: The early warning score would prompt almost all GPs to assess this patient further. This seems appropriate given the multiple risk factors, missing data and language barrier.

Greenhalgh T, *et al. BMJ Open* 2020; 10:e042626. doi: 10.1136/bmjopen-2020-042626

VIGNETTE 2: Mr Jamieson

Mr Jamieson (African-Caribbean ethnicity) calls you urgently; he is 57 and lives with his wife. He has been unwell for 9 days, but only started feeling really bad yesterday. He has absolutely no energy and finds it hard to breathe, but is wondering if that's due to his asthma (for which he normally takes a Ventolin inhaler). He isn't out of breath at rest but was very short of breath brushing his teeth this evening, whereas this morning he could do that without a problem. He doesn't sound out of breath on the phone to you, and is able to complete sentences; an attempt at making a video connection fails. He says his peak flow rate is 425 (predicted for age 510; last asthma check 450). He forced himself to get up this morning, and has spent all day in a chair, finding it a struggle even to get to the toilet. He has a BP machine because he's on medication (amlodipine) for high blood pressure. Using his machine, his blood pressure today is 120/70 and pulse 105 (recent clinic values were 148/90 and 78). He's taken his temperature with a mouth thermometer he found in the cupboard but it only reads 35 even though he's sweated so much his wife had to change the sheets. When asked about feeling hot with shivering and chills, he says yes he's had all those, quite badly on one occasion. His muscles don't ache. He has sickle cell trait, but was told this would never cause him any problems. His BMI is 33, and a recent check-up showed an eGFR of 80.

Scoring in round 1 (51 responses):

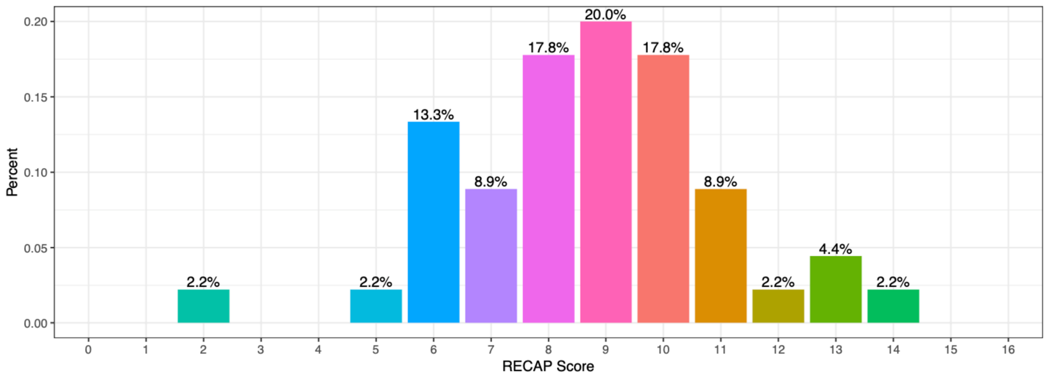

Scoring in round 2 (37 responses):

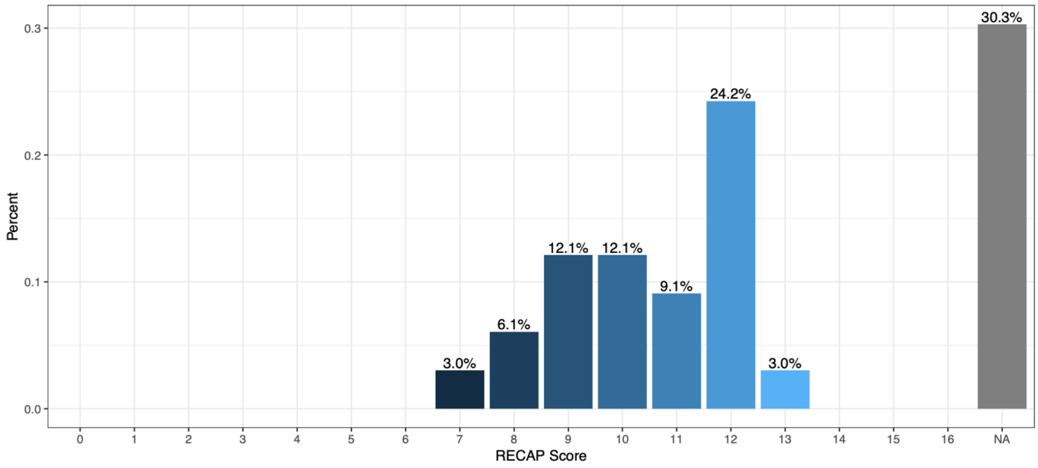

(the N/A column is because the patient has been referred urgently without completing the RECAP score)

In sum, 82% of first sample and 100% of second sample would refer this patient urgently.

Responses to individual items showed that the main discrepancies were

- Tiredness: Noticeably more tired doing usual activities (10%) versus struggling to get out of bed (90%)
- Shielded list yes 77% no 23%
- Duration of temp not applicable 42%, 8 or more days 45%
- Other risk factors for poor outcome 0-2 37%, 3 or more 63%
- Whether to refer urgently without completing RECAP or continue with RECAP
- Level of clinical concern: high 66% extremely high 31%
- Did RECAP capture your level of concern? Yes 77%, no 23% r1 and 90%, 10% r2.

Qualitative comments

Some wanted to ask him more questions about his asthma.

"Rapid deterioration in symptoms over 12 hours - high number of risks. Is at that 9-10 day tipping point for COVID - I would be admitting urgently."

"composite assessment - day 9, acute deterioration SOB, underlying asthma, risk factors. degree of SOB and deterioration is overriding concern"

"I think the profound fatigue is very concerning - I don't know that we are certain whether this is always a sign of silent hypoxia or whether it may just be due to immune response to SARS COV-2. I think I would want to check oxygen saturations, and most likely admit."

"Day 9, SOB brushing his teeth! Likely heading towards ARDS. Wouldn't want to waste time with Hub clinic with this one but would call him an ambulance."

"Although there is an argument (maybe) for see urgently in a hub - if his O2 sats and work of breathing are normal, what is hospital going to do? Xray? Rate of deterioration and duration of illness I think are the critical factors here that tip towards hospital assessment."

[The last comment above illustrates, I think, a GP who is less aware of the possibility of ARDS than the penultimate commenter above – ie RECAP score is budging this GP towards taking more aggressive action than they would otherwise have done – this is good!]

Conclusion: In every case in round 2 (where we calculated the score automatically), this high-risk patient would be referred urgently. The lower scores on round 1 may have been arithmetical or interpretation errors. Even in round 2, 31% of responders only had "moderate" or "high" clinical concern but would have been prompted to refer urgently.

VIGENTTE 3: Mrs Finlay

> *Mrs Finlay (White Irish ethnicity) is 88 and in a care home. She has multimorbidity (ischaemic heart disease, osteoarthritis, chronic pancreatitis and hypothyroidism) and on multiple medications, but her quality of life is good and she is normally mentally sharp. A recent conversation about 'ceiling of care' is documented in her medical record: she would like to be referred to hospital if appropriate. Her carers are concerned because after three days of a low-grade fever (37.5 on tympanic thermometer) and worsening breathlessness (which, since*

this morning, is present even when lying in bed), she has become delirious. Her blood pressure is 110/65 and pulse 120.

Scoring in round 1 (51 responses)

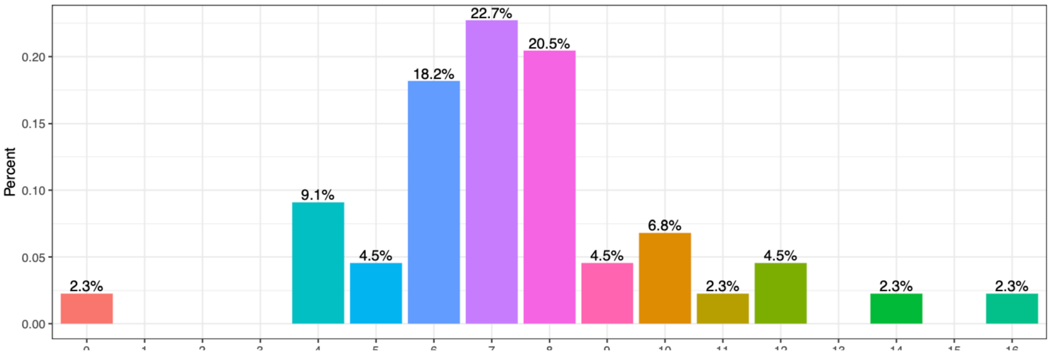

Responses to individual items showed that the main discrepancies were

- Clinical concern: moderate 7%, high 27%, extremely high 66%.
- Did RECAP reflect clinical concern? Yes 64% No 36%
- What would you do? See in hot hub 16%, refer urgently 82%

(Not included in round 2).
Qualitative comments:

"In practice, I would want a more detailed discussion with the carers about whether referral to hospital was "appropriate". Sounds like it should be, but hard to capture the nuance in a vignette. Unlike the one before or the one after, she is a hospital referral/999 as no point going to a community service if she's suitable for admission."

"Actually what I would do if at all possible is eyeball her (by video or in person) to establish if she is really delirious and very SOB. However, if I have to choose from only the 3 options above, I would have to admit. (Note: I am not sure whether you are using 'virtual ward' to include video consultation - this is not how we use it locally)."

"Given the delirium, and ceiling of care wishes, she needs an urgent work up."

"Delirium very difficult to score Also lots of causes of this in elderly eg UTI"

"Trajectory is important here, as is the new delirium and breathlessness at rest. Admit."

"RECAP itself didn't [capture my concern] (only 6, however does have 3 in one area)- however she has a red flag symptom - interestingly because of temperature/ no description of duration-/muscle aches etc, (really a lack of information as May happen in care home residents), the score is lower, but the presence of the red flag (confusion and new sobar) means that the score is less important - so overall the tool Accurately captured my concern."
[this DID capture the concern because RECAP says score 3 in one item → refer urgently]

Conclusion: This was the vignette with the most variation, due to two things: a) some GPs didn't see delirium as a red flag on the front page; b) some felt the delirium wasn't an indication to refer urgently (eg may have been a UTI). But more than 80% of GPs would refer given this story and score.

VIGNETTE 4: Mr Hughes

Mr Hughes is 72 (white British ethnicity) and lives alone. He has been unwell with Covid symptoms for 11 days. He was seen last week in the hot hub clinic because of breathlessness, and supplied with a home oximeter. Today, he feels more breathless than yesterday but says he can still walk around the living room. He's been measuring his saturations daily and the last three days have been 97%, 96%, 96%, but he feels much more tired today than he did yesterday. The oximeter measures his pulse at 98. Using a video app, you measure his respiratory rate at 24. You ask him to take 40 steps around his flat and re-measure his saturation levels. He does this, and says the level is still 96%, but a minute later it has fallen to 94%. His temperature with a mouth thermometer is 37.6 and he hasn't had shivers or chills. He had prostate cancer 10 years ago and was treated for depression last year after his wife died. His BMI is 28.

Scoring in round 1 (51 responses)

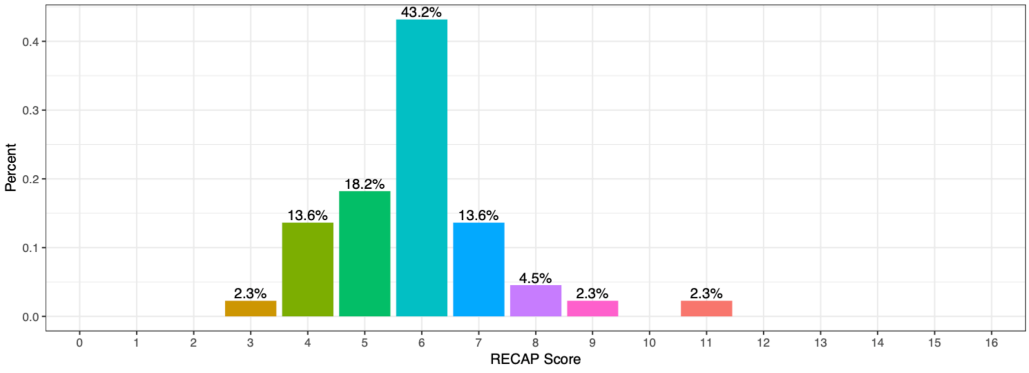

(no round 2 with this case)

In sum, 22% of this sample would refer this patient urgently; all but one responder would review in hot hub or at home.

Responses to individual items showed that the main discrepancies were

- Duration of temp: not applicable 42%, 8 or more days 45%
- Risk factors for poor outcome: 0-2 36%, 3 or more 64%
- Clinical concern: low 7% moderate 52% high 36% extremely high 5
- Did RECAP reflect clinical concern? Yes 80% No 20%
- What would you do? Reassure and advise 16%, See in hot hub 50%, refer urgently 32%

In sum, there was quite a bit of variation in what GPs subjectively felt was needed, but in all but one case, the patient would have had a hot hub review or hospital referral if score was followed.

Qualitative comments:

"Moderate risk - think the desaturations on walking are main red flag here"

"The post exertional hypoxia is a concern, and the isolation but there are psychosocial factors that might contribute to his malaise. He is not so ill that he needs hospital and a prompt Hub appointment could suffice."

"Deteriorating despite home management, desaturating, and vulnerable living alone. I'm not sure here what the hot hub would add to his assessment. I think he needs ED review."

"He already has a pulse oximeter. He will need daily calls but is probably safe to be managed remotely today."

Conclusion: The spread here was because some GPs did not interpret exertional desaturation as a serious 'red flag' sign. The RECAP score pushed those GPs to reviewing the patient urgently or sending him to hospital. This is what the score is designed to do.

VIGNETTE 5: Mr Liu

> *Mr Liu is a 37-year old postgraduate student from Taiwan who lives in a shared house of multiple occupation. He's worried because he has been unwell for 10 days and has a cough and a high fever (measured at 39 with a mouth thermometer). He's also finding it hard to catch his breath – something that's been going on for three or four days. He doesn't think it's worse today than yesterday. He doesn't know his housemates well, but he knows two of them have been unwell. He is not registered with your practice so you have no previous records on him, but he tells you he might have been diagnosed with diabetes back in Taiwan, he's not sure. His father had it and he is a bit overweight, though he doesn't know his actual height and weight. He's been lent an oximeter by one of his housemates and taken a reading – his pulse is 115 and saturation 95%. On a video call, you think he looks pale and anxious, and his respiratory rate is 22.*

Scoring in round 1 (51 responses)

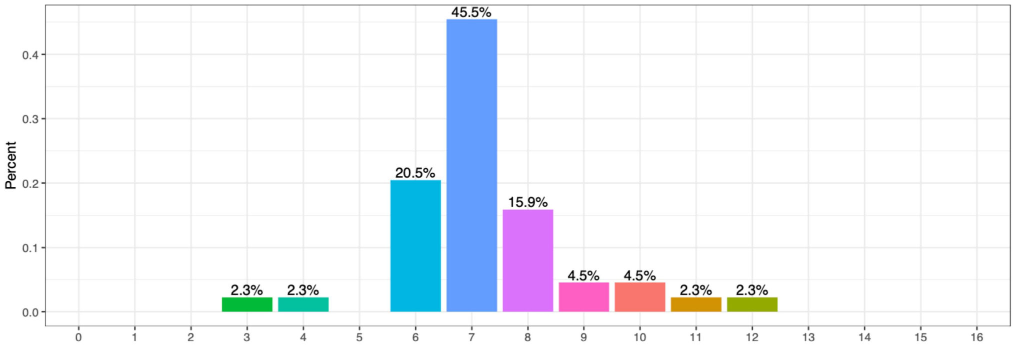

Scoring in round 2 (37 responses)

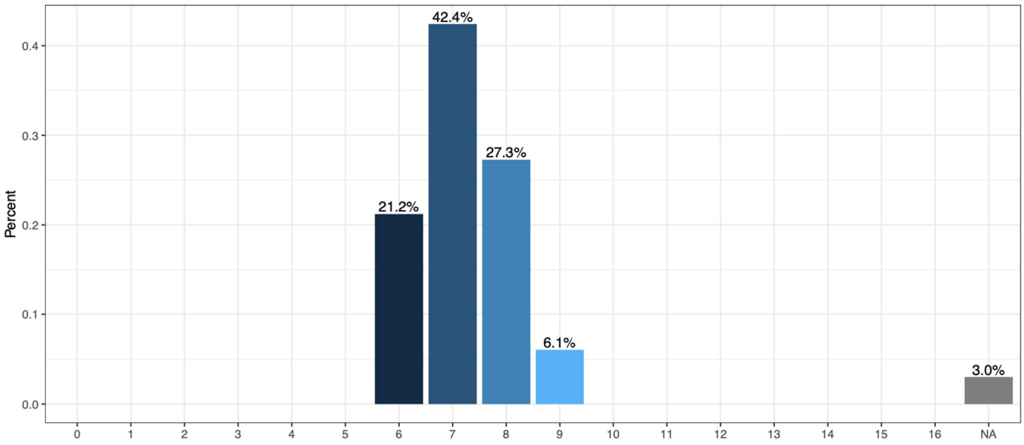

Greenhalgh T, et al. BMJ Open 2020; 10:e042626. doi: 10.1136/bmjopen-2020-042626

- Level of clinical concern r1 low 2%, moderate 52%, high 39%, extremely high 7%; r2 moderate 3%, high 66%, extremely high 31%.
- What would you do? R1 reassure and advise 5%, see in hot hub 65%, refer urgently 27%; R2 Reassure and advise 18%, See in hot hub 48%, refer urgently 33%
- Did score capture level of clinical concern r1 yes 80% no 20%; r2 90%, 10%.

In sum, 75% of r1 and 80% of r2 would have referred this man urgently.

Qualitative comments

“I think it came up higher than I would have expected.”

“score suggests consider urgent referral - well I’ve considered and am happy for him to be seen in hub. score would be higher still if he has DM and BMI >35 - which Ive not scored - but why he needs to be seen rather than have phone advice.”

“I think there’s a degree of uncertainty around this case. We don’t know the patient and there is some uncertainty regarding his PMH, but the safest thing is to assume he does have diabetes. It can be quite hard to differentiate between anxiety and an unwell patient over video especially, and paleness and tachycardia makes me concerned regarding shock. I would be admitting him.”

“During vignette, I was wondering would I really want to see this patient in a hot hub, to be knower, with Sats of 95 in a fit and well young man; that is worrying enough to me So yes I think overall though not so barn door, I feel direct referral to hospital is appropriate for him mainly given his sats/ duration etc”

Conclusion: This vague case had a number of potentially worrying features and inevitably they were interpreted slightly differently by the responders, but in every case the patient would have either been referred straight to hospital or seen in a hot hub. Once again, the score seems to be doing its job of prompting the GP to act on a sign or symptom they may not be aware is associated with poor prognosis.

ADDITIONAL COMMENTS

Complaints about layout and not seeing score in front of them.

Complaints about time it took.

--

TG, PT
1.6.20

Greenhalgh T, *et al. BMJ Open* 2020; 10:e042626. doi: 10.1136/bmjopen-2020-042626
