# Supplementary material for: What items should be included in an early warning score for remote assessment of suspected COVID-19? qualitative and Delphi study
Source: BMJ Open. 2020 Nov 12;10(11):e042626. doi: 10.1136/bmjopen-2020-042626 (PMC7662139; doi:10.1136/bmjopen-2020-042626)
Supplement: Supplementary data [file bmjopen-2020-042626supp004.pdf]

Appendix A4: Full version of the RECAP-V0 score

REMOTE COVID-19 ASSESSMENT FOR PRIMARY CARE

This is the simulated early warning score used in the Delphi exercise in the qualitative (item development) phase of the RECAP study. The score is designed to support assessment of, and communication about, COVID-19 patients at the primary-secondary care interface. It is not intended to replace clinical judgement. It has been produced by professional consensus but has not yet been tested against clinical outcomes or formally validated.

If the RECAP score is high, it will not tell you whether to refer for active management (e.g. hospital or hot hub), or arrange palliative care (other factors will of course influence this decision).

RED ALERT CRITERIA: If patients have any of the following, consider 999

Adapted from national primary care covid-19 recommendations

Shock or peripheral shutdown

- New confusion (including delirium)
- Reduced level of consciousness
- Extremities – cold and clammy to touch
- Pallor – skin is mottled, ashen, blue or very pale
- Reduced urine output – little or no urine in last 24 hours

Severe breathlessness

- Rapid, significant deterioration in breathing in last hour
- New breathlessness at rest
- Newly unable to complete sentences
- Sudden onset of breathlessness

Other red flags which may be non-covid-19 related e.g.

- Severe central chest pain
- Collapse

RECAP-V0 SCORE FOR PATIENTS WHO DO NOT HAVE RED ALERT SYMPTOMS OR SIGNS

		Score 0	Score 1	Score 2	Score 3 => refer urgently	Score
1	Heart rate (per minute)<br>*(if heart rate not available, score 1)*	51-90	41-50 or 91-110<br>or missing data	111-130	≤ 40 OR > 130,<br>if unexplained	

Use medically approved device if available, or patient's own. Lower threshold for tachycardia by 10 bpm if beta-blocker or other heart-slowing drug taken in past 24h, but use standard thresholds for bradycardia. Adjust score if known to have physiological bradycardia (e.g. athlete).

		Score 0	Score 1	Score 2	Score 3 => refer urgently	Score
2a	Shortness of breath	Not breathless at all	Breathless on moderate exertion e.g. walking room to room	Breathless on mild exertion e.g. getting out of a chair	Severe breathing difficulty; can't complete sentences at rest	Highest of 2a or 2b
2b	or Respiratory rate (per minute)	12-20	21-24	9-11 or<br>25-29	8 or less, or<br>30 or more	

Score any breathlessness that patient or carer is concerned about. Take account of pre-existing conditions such as COPD. Assess respiratory rate by video, ask patient to place hand on chest. An anxious patient may be hyperventilating.

		Score 0	Score 1	Score 2	Score 3 => refer urgently	Score
3	Trajectory of breathlessness	Same or better than yesterday	Breathless, worse than yesterday	-	Significant deterioration in last hour	

Pay careful attention to a history of recent worsening of breathlessness, especially if this is what concerns patient or carer.

		Score 0	Score 1	Score 2	Score 3 => refer urgently	Score
4a	Oxygen saturation at rest	96%<br>or above	95% (don't do 40-step test unsupervised)	94% (don't do 40-step test unsupervised)	93% or below (don't do 40-step test)	Highest of 4a, 4b & 4c
4b	or Saturation after 40 steps	Fall of 0-1%	-	Fall of 2%	Fall of 3% or more	
4c	or Profound tiredness or fatigue	None<br>or mild	Noticeably more tired doing usual activities	Struggling to get out of bed	Unable to speak because of tiredness	

Patient should have warm hands and place oximeter device correctly. Lower thresholds if patient has chronic lung disease with known hypoxia (typically by 6%, but will vary – use usual baseline readings to adjust if known). Do exertion test only if clinician in attendance or if saturation is 96% or higher at rest (lower threshold for chronic lung disease). Saturation levels may fall for 1 min after stopping exercise. Most patients

with covid-19 feel some fatigue, but profound fatigue may be a feature of 'silent hypoxia'. Take account of patient's baseline level of fatigue.

5a	Temperature	≤ 38 °C	38.1-39 °C	> 39 °C or < 35 °C	-	Highest of 5a or 5b
5b	or Feeling feverish with shivers	None	Feverish or chills	Uncontrollable shivering	-	

A tympanic thermometer is preferred. Use highest recorded level in last 24 hours. A low reading may reflect user error.
If the patient has no reliable thermometer (or in addition to the temperature reading), explore for a description consistent with rigors.

6	Time from first symptom (days)	7 or fewer	8 or more	-		

Number of days since onset of first symptom

7	Muscle aches	None or mild	Moderate	Severe		

8	Cognitive decline	No	Less mentally alert than usual	New and worsening confusion	Reduced level of consciousness	

9	On COVID-19 shielded list (or has been inadvertently left off it)?	No	Yes	-	-	

Includes: • organ transplant • current chemotherapy or immunotherapy • severe lung condition such as cystic fibrosis • sickle cell anaemia • high dose steroids or other immunosuppressants • blood or bone marrow cancer • lung cancer on radiotherapy • splenectomy

10	Other risk factors for poor outcome in COVID-19?	0-2	3 or more	-	-	

For example: *• Age > 65 • BMI > 35 • male • non-White ethnicity • diabetes • hypertension • coronary heart disease • chronic kidney disease • vulnerable and isolated*

TOTAL	

Clinical concern component (be guided by clinical concern whatever the RECAP score; do not add this into the score but use it to over-ride the score if appropriate):

After assessing the patient, what is your level of clinical concern (regardless of RECAP score)?	Low	Moderate	High	Extremely high

RECAP score	Meaning	Recommended action
7 or more total or 3 on any item or extremely high level of clinical concern	HIGH RISK	Consider urgent referral
4-6 or more total or high level of clinical concern	MODERATE RISK	See in hot hub or virtual ward, or arrange home visit
0-3 total	LOW RISK	Advice and monitor at home
