# Supplementary material for: What items should be included in an early warning score for remote assessment of suspected COVID-19? qualitative and Delphi study
Source: BMJ Open. 2020 Nov 12;10(11):e042626. doi: 10.1136/bmjopen-2020-042626 (PMC7662139; doi:10.1136/bmjopen-2020-042626)
Supplement: Author's manuscript [file bmjopen-2020-042626.draft_revisions.pdf]

BMJ Open is committed to open peer review. As part of this commitment we make the peer review history of every article we publish publicly available.

When an article is published we post the peer reviewers' comments and the authors' responses online. We also post the versions of the paper that were used during peer review. These are the versions that the peer review comments apply to.

The versions of the paper that follow are the versions that were submitted during the peer review process. They are not the versions of record or the final published versions. They should not be cited or distributed as the published version of this manuscript.

BMJ Open is an open access journal and the full, final, typeset and author-corrected version of record of the manuscript is available on our site with no access controls, subscription charges or pay-per-view fees (http://bmjopen.bmj.com).

If you have any questions on BMJ Open's open peer review process please email info.bmjopen@bmj.com

Remote COVID-19 Assessment in Primary Care (RECAP) early warning score: item development

Journal:	*BMJ Open*
Manuscript ID	bmjopen-2020-042626
Article Type:	Original research
Date Submitted by the Author:	11-Jul-2020
Complete List of Authors:	Greenhalgh, Trisha; University of Oxford, Nuffield Department of Primary Care Health Sciences<br>Thompson, Paul; University of Oxford, Department of Experimental Psychology<br>Weiringa, Sietse; University of Oxford, Nuffield Department of Primary Care Health Sciences<br>Neves, Ana Luisa; Imperial College London,<br>Husain, Laiba; University of Oxford, Nuffield Department of Primary Care Health Sciences<br>Dunlop, Merlin; Ardens Ltd<br>Rushforth, Alexander; University of Oxford, Nuffield Department of Primary Care Health Sciences<br>Nunan, David; University of Oxford, Nuffield Department of Primary Care Health Sciences<br>de Lusignan, Simon; Oxford University, Nuffield Department of Primary Care Health Sciences<br>Delaney, Brendan; Imperial College London, Surgery and Cancer
Keywords:	INFECTIOUS DISEASES, Health informatics < BIOTECHNOLOGY & BIOINFORMATICS, VIROLOGY

SCHOLARONE™
Manuscripts

I, the Submitting Author has the right to grant and does grant on behalf of all authors of the Work (as defined in the below author licence), an exclusive licence and/or a non-exclusive licence for contributions from authors who are: i) UK Crown employees; ii) where BMJ has agreed a CC-BY licence shall apply, and/or iii) in accordance with the terms applicable for US Federal Government officers or employees acting as part of their official duties; on a worldwide, perpetual, irrevocable, royalty-free basis to BMJ Publishing Group Ltd ("BMJ") its licensees and where the relevant Journal is co-owned by BMJ to the co-owners of the Journal, to publish the Work in this journal and any other BMJ products and to exploit all rights, as set out in our licence.

The Submitting Author accepts and understands that any supply made under these terms is made by BMJ to the Submitting Author unless you are acting as an employee on behalf of your employer or a postgraduate student of an affiliated institution which is paying any applicable article publishing charge ("APC") for Open Access articles. Where the Submitting Author wishes to make the Work available on an Open Access basis (and intends to pay the relevant APC), the terms of reuse of such Open Access shall be governed by a Creative Commons licence – details of these licences and which Creative Commons licence will apply to this Work are set out in our licence referred to above.

Other than as permitted in any relevant BMJ Author's Self Archiving Policies, I confirm this Work has not been accepted for publication elsewhere, is not being considered for publication elsewhere and does not duplicate material already published. I confirm all authors consent to publication of this Work and authorise the granting of this licence.

For peer review only

Original research paper submitted to BMJ Open June 2020

Remote COVID-19 Assessment in Primary Care (RECAP) early warning score: item development

For peer review only

Trisha Greenhalgh (Professor)[1]

Paul Thompson (Researcher) [2]

Sietse Weiringa (Researcher)[1]

Ana Luisa Neves (Researcher)[3]

Laiba Husain (Researcher)[1]

Merlin Dunlop (GP)[4]

Alex Rushforth (Researcher)[1]

David Nunan (Researcher)[1]

Simon de Lusignan (Professor)[1]

Brendan Delaney (Professor)[3]

For peer review only - http://bmjopen.bmj.com/site/about/guidelines.xhtml

[1] Nuffield Department of Primary Care Health Sciences, University of Oxford, Oxford OX2 6GG, UK

[2] Department of Experimental Psychology, University of Oxford, Oxford OX2 6GG, UK

[3] Institute of Global Health Innovation, Imperial College London, London

[4] Ardens-Q Ltd

Corresponding author: Professor Trisha Greenhalgh, Nuffield Department of Primary Care Health Sciences, University of Oxford, Oxford OX2 6GG, UK - phone: +44 (0)1865 289294, email: trish.greenhalgh@phc.ox.ac.uk.

Keywords: Early warning score, RECAP (Remote Covid Assessment in Primary Care) score, COVID-19, Medical record systems, computerized.

Word count: 3956

For peer review only

ABSTRACT

Background

To develop items for an early warning score (RECAP: REmote COVID-19 Assessment in Primary Care) for patients with suspected COVID-19 who need escalation to next level of care.

Methods

The study was based in UK primary health care. The mixed-methods design included rapid review, Delphi panel, interviews, focus groups and software development. Participants were 112 primary care clinicians and 20 patients recovered from COVID-19, recruited through social media and snowballing. Using rapid literature review, we identified signs and symptoms which are commoner in severe COVID-19. Building a preliminary score from these data, we ran four rounds of an online Delphi panel with 72 clinicians, the last incorporating fictional vignettes, collating data on R software. We refined the score iteratively in response to quantitative and qualitative feedback. Items in the penultimate score were checked against narrative interviews with 20 COVID-19 patients. We required, for each item, at least 80% clinician agreement on relevance, wording and cut-off values, and that the item addressed issues and concerns raised by patients. In focus groups, 40 clinicians suggested further refinements and discussed workability of the instrument in relation to local resources and care pathways. This informed design of an electronic template for primary care systems.

Results

The pre-validation RECAP-V0 score comprises a red flag alert box and 10 assessment items: pulse, shortness of breath or respiratory rate, trajectory of breathlessness, pulse oximeter reading (with brief exercise test if appropriate) or symptoms suggestive of hypoxia, temperature or fever symptoms, duration of symptoms, muscle aches, new confusion,

For peer review only - http://bmjopen.bmj.com/site/about/guidelines.xhtml

shielded list, and known risk factors for poor outcome. It is not yet known how sensitive or specific it is.

Conclusions

Items on this preliminary score align strongly with published evidence, clinical judgement and patient experience. We are conducting an urgent validation study and invite other researchers to work alongside us.

Study registration

Clinicaltrials.gov (NCT04435041).

Article summary

Strengths and limitations of the study

- First systematic study to develop a COVID-19 early warning score for primary care
- Captures clinician and patient experience of the deteriorating patient with COVID-19
- Combines extensive qualitative research and quantitative consensus methodology
- Conducted at speed in the midst of first wave of COVID-19
- Items have strong face validity but formal validation is still ongoing

For peer review only

Introduction

Some patients with COVID-19 experience deterioration (usually at around day 8),[1 2] so there is a need for research to develop accurate early warning scores – clinical prediction models designed to identify patients who need escalation to next level of care.[3] Such scores need to be both highly sensitive (detecting all patients who need onward referral) and fairly specific (excluding all or most patients who do not). A recent systematic review of prediction models for COVID-19 concluded that "proposed models are poorly reported, at high risk of bias, and their reported performance is probably optimistic" (page 2).[4] That review identified no evidence-based prediction models for primary care settings.

Assessment of a patient with suspected COVID-19 in primary care is fraught with uncertainty, since it is a new disease whose clinical course does not mirror other pneumonias[5] and because most patients will be assessed remotely (i.e. by phone or video).[6] The UK Royal College of General Practitioners has cautiously endorsed the use of NEWS2 (National Early Warning Score 2) alongside clinical judgement for the assessment of patients with COVID-19.[7] NEWS2 is calculated from the patient's temperature, pulse rate, respiratory rate, systolic blood pressure, pulse oximetry reading and new confusion.[8] The National Institute for Health and Care Excellence (NICE) rapid guideline on management of COVID-19 pneumonia in the community makes the guarded statement that the NEWS2 score "may be useful" in assessing deterioration but that the patient should not be brought in for a face-to-face assessment solely to calculate a NEWS2 score (paragraph 3.7).[9] But NEWS2 was developed for a different purpose (see Discussion), and requires data that may be difficult to obtain. A recent preprint suggest that it correlates poorly with severity of COVID-19.[10]

We sought to develop and validate a primary care early warning score for COVID-19 based on data that can be reliably collected during a remote consultation. This paper describes the development of a version 0 of the score which can be formally validated. It does not cover the actual validation of the score.

For peer review only - http://bmjopen.bmj.com/site/about/guidelines.xhtml

Method

Study design and governance

The study was part of the Remote By Default research programme, funded by UK Research and Innovation COVID-19 Emergency Research Fund, and overseen by an independent advisory group with a lay chair and a separate patient advisory group. It consisted of five phases (Figure 1): rapid review, a four-round Delphi panel of primary care clinicians, semi-structured interviews with patients, focus groups with primary care clinicians, and electronic template development. Ethics approvals are included at the end of the paper.

FIGURE 1

Rapid review

Detailed methods have been published elsewhere [5]. Briefly, fortnightly keyword searches were conducted from March to June 2020 of PubMed for English-language systematic reviews and LitCovid and MedRxiv for reprints. Methodological quality of reviews was assessed using AMSTAR-2.[11] Primary studies within included reviews were cross-checked for duplication before extracting data on each symptom or sign in both mild and severe COVID-19. The synthesis was continually updated over the course of the study period from the most up-to-date, highest-quality systematic reviews. To construct the draft RECAP score, we selected symptoms and signs that were significantly commoner in severe than mild disease.

Delphi panel

Delphi is a structured approach to working towards consensus.[12] Steps include defining a problem, selecting a panel of experts (both academics and clinicians), supplying evidence along with key uncertainties, collecting quantitative (numerical scores) and qualitative (free text) data on a set of statements, feeding scores and comments back to panel members, and repeating until residual disagreement cannot be resolved. Advantages method include practicality (it can be done online, asynchronously, without specialist tools), anonymity

For peer review only - http://bmjopen.bmj.com/site/about/guidelines.xhtml

(participants know the average group score but not individuals' scores) and iteration (feedback prompts outliers to either defend their score to the group or change it).[13 14]

Following established methodology,[12] we used a social media announcement (TG's Twitter account) and snowballing from people thus recruited to obtain 72 volunteers (68 general practitioners, three nurse practitioners and one paramedic), all of whom were actively involved in assessing acutely ill patients with suspected COVID-19. Ten of these were clinical academics. The 'draft 0.1' RECAP score developed from rapid review was entered onto a questionnaire using Survey Monkey (homeworking during lockdown prevented us from accessing our usual professional survey software).

We sent brief instructions to participants, including a summary of what was known about prognostic factors in COVID-19. We gave them five days to complete the survey, scoring each item for relevance (should it be included in the score?) and wording (how would they improve it?), and add free text comments. We sent two prompts to non-responders. A researcher (PT) imported the raw data into R and collated quantitative responses using simple descriptive statistics (percentage selecting, for example, yes as currently worded, yes but change wording, not sure, probably not and definitely not), and tabulated qualitative responses, which TG subsequently arranged under broad clinical categories and themes. We amended the draft RECAP score before circulating a summary of data, notes on changes and reasons for them, and the next iteration of the score. This was repeated for a second and third round.

The fourth round included only three residual items on which we had not yet achieved our goal of 80% consensus, plus a set of five fictional vignettes which incorporated various scenarios (such as missing data, untrustworthy technology, complex social circumstances, and comorbidities) that participants had proposed in their free text responses. They were asked to calculate a RECAP score for each of these five vignettes and indicate their level of clinical concern. This fourth round was repeated following a report from several participants that they had been unsure how to calculate the RECAP score (so had guessed); in the repeat round, we adjusted the software to calculate the score automatically.

For peer review only - http://bmjopen.bmj.com/site/about/guidelines.xhtml

Patient interviews

UK-based survivors of COVID-19 were recruited using a social media call (again, TG's Twitter account) and snowballing from those who responded (to access those not on social media).

AR conducted 20 audio recorded interviews by telephone or MS Teams, taking verbal consent. Patients were asked to narrate their various contacts with healthcare services, including how symptoms were assessed by call handlers or clinicians. Relevant sections of interviews were transcribed and read independently by AR and TG. Data were extracted in to a Word document and arranged into categories to correspond to the items on the RECAP score, and any additional categories not included in the score.

Clinician focus groups

Focus groups are a qualitative method designed to capture both individual perspectives and group dynamics (such as empathy, humour and conflict).[15 16] There is an established methodology for conducting them by video-conference.[17] A new sample of 40 primary care clinicians (28 general practitioners, 11 nurse practitioners, 1 paramedic) who regularly assess suspected COVID-19 patients was recruited by a social media invitation enhanced by snowballing. Focus groups were held by Zoom and lasted 90 minutes. Prior to the group, we circulated ground rules (e.g. about confidentiality) and invited participants to read and score the five vignettes to familiarise themselves with RECAP-V0. Each focus group was facilitated by two academic general practitioners (TG, ALN or SW) guided by a list of prompt questions (reproduced in Additional File 1 on bmj.com). Briefly, we sought their views on the RECAP-V0 score (including a mock-up of the electronic template), and how they felt it would align with local clinical presentations, care pathways, electronic templates, resources and workflows. We asked what challenges they anticipated with its implementation.

Focus groups were video-recorded with consent. They were not fully transcribed but the clinical researchers all listened independently and transcribed selected sections. This free text was organised thematically on an Excel spreadsheet using the Framework method.[18]

For peer review only - http://bmjopen.bmj.com/site/about/guidelines.xhtml

Insights from focus groups were fed iteratively into the software design phase described below.

Electronic template design and development

MD and BD matched individual RECAP-0 items with existing SNOMED-CT codes where possible. A template was constructed using drop-down menus, and inserted as a supplement to the Ardens COVID-19 assessment template built for EMIS Web (Egton Medical Information Systems). It included a prompt to capture the patient's verbal consent for data linkage, as well as data previous entered and saved as part of the standard COVID-19 assessment. Layout and ordering of items was adapted in response to focus group comments and insights. Once the EMIS template is validated, a version will be built for use in SystmOne (the other major provider of English GP clinical systems) and Adastra (the system used in out-of-hours) using the same parameters and codes as the EMIS version.

Results

Rapid review

Five factors appeared to predict poor outcome in COVID-19: persistent fever, shortness of breath, low oxygen saturation, muscle aches, and certain comorbidities.[5] Patients with severe COVID-19 may develop shock (manifest, for example, as deteriorating conscious level, hypotension, and reduced urine output). Based on these findings, we constructed a preliminary 13-item score. Later in the study, additional questions emerged which prompted new rapid reviews: assessment of breathlessness,[19 20] how to measure exertional desaturation accurately and safely in patients with suspected COVID-19,[21] and the reliability of smartphone oximeters[22] and smartphone blood pressure measuring apps.[23]

For peer review only - http://bmjopen.bmj.com/site/about/guidelines.xhtml

Delphi panel

The Delphi panel was conducted in April and May 2020. Round 1 had a sample of 69 clinicians; three more joined for rounds 2 to 4. Of these, 62, 53, 56 and 51 completed the surveys respectively – response rates of 90%, 74%, 78% and 71%. Progression towards quantitative consensus across the four rounds is shown in Table 1.

TABLE 1

In sum, at least 80% agreement on relevance, wording and cut-off values for particular scores was eventually achieved for eight variables: pulse, temperature, symptoms of fever (for use particularly if the patient does not have a reliable thermometer), oxygen saturation level, tiredness (which, if severe, may indicate hypoxia and hence potentially substitute for an oximeter reading), muscle aches, and known risk factors (comorbidities). Additional items with moderate agreement included demographic risk factors (77% agreement), oxygen saturation level after a 40-step exercise test (75%), trajectory of breathlessness (73%) and duration of temperature (63%).

Our qualitative dataset from the Delphi panel included over 200 pages of comments. Key themes are summarised and illustrated in Additional File 2 on bmj.com. These, along with rapid review, allowed us to characterise the clinical features of the deteriorating COVID-19 patient in primary care (Box 1)

BOX 1

In addition to prompting new rapid reviews, the Delphi qualitative data shaped the development of the score in several ways. Particular forms of words (for example, to question patients about the severity and rate of deterioration of breathlessness) enabled us to refine our items and cut-off levels. Participants alerted us to existing guidance and protocols used either nationally or locally (there was strong consensus that any new score should complement rather than replace these). Comments about missing or untrustworthy data when a patient was being assessed at home via telephone or video link prompted us to develop default scores or alternative questions to compensate for such deficiencies. The

For peer review only - http://bmjopen.bmj.com/site/about/guidelines.xhtml
For peer review only

free text comments included rich data, based on real clinical experiences, from which we were able to construct the vignettes used in discussions. Numerous comments on the practicalities of applying the score prompted us to set up focus groups to explore these operational challenges further.

The five fictional vignettes and the results of the scoring exercise on these are reproduced in Additional File 3 on bmj.com. In sum, whilst there was considerable variation in the actual score given (for example, in whether clinicians judged an ill-defined set of symptoms as "moderately" or "severely" tired), in all five vignettes the RECAP score as calculated appeared to prompt an appropriate and cautious response. For example, in a case of an elderly South Asian patient who spoke no English and with no equipment at home for the family to take measurements, all but one participant were prompted by the RECAP score to assess the patient in a face-to-face encounter. In a case of an African Caribbean man with profound tiredness and rapidly worsening breathlessness in the second week of his illness, all participants were prompted to arrange urgent transfer to hospital. Free text comments in several vignettes indicated that some respondents' level of clinical concern had not been especially high and that they were surprised that the RECAP score was so high.

Patient interviews

Of the 20 patient interviews, 15 included descriptions of deteriorating symptoms in the acute phase of COVID-19. These included worsening breathlessness, inability to speak in sentences, profound fatigue without feeling short of breath, high temperature, and anxiety. Patients also described being reassured by remote assessments using video examination and home monitoring equipment.

> *"… when I was speaking to them they were listening to me breathing and watching my chest and things. And they were seeing my breathing through the video link, wanting to see a clear vision of my chest area and counting my respiratory rate. And looking back when I spoke to them I was breathless. […] I've already got a pulse oximeter and a blood pressure machine. […] The pulse ox[imeter] was really good.*

For peer review only - http://bmjopen.bmj.com/site/about/guidelines.xhtml

What I had to do is when they video called I had to put it on my finger and wave it at the screen and they were able to monitor it." *[patient NM1]*

Some patients described long waits to get through to NHS 111 (the English telephone triage service), being asked "tick box" questions by call handlers or clinicians, and feeling dismissed on the basis of such questions even when they were concerned about the extent and pace of their own (or a relative's) deterioration. For example:

"You can't make a diagnosis on the phone because you don't get to see the physical symptoms. They [clinicians] never saw the [patient's] rash, I described it but they didn't actually get to see it. They couldn't see she was sweating all around her hairline and her face was super pale. The overall picture they couldn't see – they just had written down some numbers." [relative of patient, HG1]

The "exercise" being referred to in the quote below is probably the Roth score,[24] which is likely neither sensitive nor specific in the assessment of COVID-19 patients.[20]

"There was an exercise where I need to count to something a certain number of times. They did a small exercise. But now they know you can have severe hypoxia without clear shortness of breath when talking. That was the assessment. They said 'I know it's rough, solider through. You're 34 years old. There's no point in escalating this.'" *[patient SN1]*

These patient data helped us refine the wording of the questions on the RECAP score and also the clinical description in Box 1. In particular, the patient data emphasised the importance of developing the score as an adjunct to expert clinical judgement and not as a substitute for it.

Focus groups

In focus groups, clinicians described COVID-19 as a disease with "surprises". They were especially concerned with patients whose initial course was unremarkable but who

For peer review only - http://bmjopen.bmj.com/site/about/guidelines.xhtml

subsequently deteriorated rapidly. They wanted an early warning score not primarily to identify “red flag” patients (whose need for urgent hospital referral they felt was usually obvious), but for assessing moderately sick patients and tracking their progress over time. They considered the RECAP score to have high face validity and to reflect their level of clinical concern on the vignettes. Some felt that RECAP would be less useful for assessing an unwell patient who did not have COVID-19. All three groups proposed the same additional item on the score (patient less alert than usual or new confusion), and there was also agreement on a minor change to one item (changing “duration of temperature” to “duration of symptoms”). Participants made suggestions for layout and ordering of items on the electronic template, emphasising simplicity, ease of use and “human factors”.

Electronic template design and development

MD worked with BD and others to adapt the existing Ardens COVID19 clinical template for EMIS Web to ensure that the codes used were consistent with the required data capture for the RECAP score. Once the validation stage is complete, the score will be integrated into its own template page within EMIS and other electronic systems and, subject to available codes and regulatory considerations, will assist the user in calculating the total score.

Discussion

Statement of principal findings

This mixed-method study has produced five key findings. First, we have developed, through consensus, a rich description of clinical deterioration in the primary care patient with COVID-19 (Box 1). Second, we have achieved very high agreement among 72 front-line primary care clinicians and academics for the inclusion of a particular wording on the following variables for inclusion in an early warning score: pulse rate (88% agreement), temperature (89%), fever symptoms (91%), respiratory rate (84%), shortness of breath (79%), pulse oximeter reading (80%), post-exercise fall in pulse oximeter reading (75%), tiredness (88%), muscle aches (82%), shielded list (86%), and other risk factors for poor

For peer review only - http://bmjopen.bmj.com/site/about/guidelines.xhtml

outcome (77%), along with affirmation of these by a second sample of 40 clinicians, who added duration of symptoms and new confusion. Third, we have obtained a high level of agreement on the numerical or descriptive cut-off levels for allocating points to different signs and symptoms (details in Table 1). Fourth, we have confirmed that the items on the score reflect the concerns of patients. Finally, we have surfaced, and begun to address, some of the human factors and operational challenges of implementing the RECAP-V0 score in different primary care services and settings across the UK.

Strengths and limitations of the study

This is the first study systematically to capture the clinical experience and wisdom of front-line primary care practitioners, as well as the experience of patients, in relation to the assessment of the deteriorating COVID-19 patient who has not yet been referred to hospital. Early Chinese studies on COVID-19 included only hospital patients.[1 25] Other community-based studies identified symptoms (notably loss of smell) associated with COVID-19 but which lack prognostic significance.[26]

Our study was undertaken at speed, in the midst of the first wave of the pandemic, with a judicious trade-off between rigour and speed. Despite these pressures, we believe we have achieved a sufficiently large and diverse sample of front-line practitioners and patients to be confident that the findings reflect current best practice and patient priorities.

The main limitation is that we have not yet validated the score (see ‘Next steps’ below). Another limitation is that the score relies partly on UK-specific data (specifically the ‘shielded’ category). This aspect of the score is, however, readily adaptable to categories used in other countries. Because the score is preliminary, we do not yet have firm evidence that the weightings assigned to different items and cut-off levels are appropriate.

Comparison with previous literature

To date, most early warning scores have been developed for use in hospital inpatients using routinely collected vital sign data.[27] In UK hospitals, the NEWS2 score has become a

For peer review only - http://bmjopen.bmj.com/site/about/guidelines.xhtml

common language of sickness with positive implications for patient safety (especially in relation to sepsis).[28] NEWS2 is recommended by NICE guidelines both in general[29] and as a component of the critical care of COVID-19 patients,[30] though it has been heavily criticised even in hospital settings.[27 31-34] NEWS2 has been used in pre-hospital settings by ambulance crews[31] and in early detection of suspected sepsis in primary care[35]. However, it has not been formally validated in general practice,[36] so its sensitivity and specificity in that context are unknown. Its positive predictive value is low even in hospital and ambulance settings, and is likely to be even worse in primary care because serious illness is rare.[37] A rise in NEWS2 appears to be a relatively late indicator of deterioration, typically triggering only in the last 12 hours before transfer to critical care.[27] For all these reasons, NEWS2 might cause harm from both under- and over-referral in a primary care setting, though there is preliminary evidence that it may have some value for COVID-19 in care home residents, who tend to be sicker.[38]

The key differences between NEWS2 and RECAP-0 are as follows. Whereas NEWS2 was designed to be calculated from observations taken in hospital by trained staff and is based solely on signs, RECAP is designed to be completed in primary care as part of a clinician-patient conversation along with a (limited) remote physical examination, and is based on both symptoms and signs. With the exception of pulse rate (which defaults to a value of 1 if no reading is possible), all items requiring a physical examination include an alternative item based solely on symptoms. This will allow the clinician to populate the score even when the assessment is being done remotely and the patient is unable or unwilling to use equipment, thereby reducing the danger of missing data.[27] Whereas NEWS2 is promoted as disease-agnostic, RECAP-0 was designed to be disease-specific to pick up the clinical trajectory shown in Box 1 (though there is no evidence yet that it is less sensitive or specific than NEWS2 for non-COVID-19 conditions).

The RECAP-0 score, which is based largely on professional consensus at this stage, is nevertheless a significant improvement on existing primary care triage tools for COVID-19, which appear to have been developed pragmatically.[27]

For peer review only - http://bmjopen.bmj.com/site/about/guidelines.xhtml

Next steps

The transparent reporting of a multivariable prediction model for individual prognosis or diagnosis (TRIPOD) statement[39] states that development of a prognostic model (of which an early warning score is one example) requires two phases: instrument development and instrument validation. The study reported here has ensured that the first component of instrument development has captured three important dimensions: the existing evidence base from the research literature; the experience and intuition of front-line primary care clinicians; and the experience of patients.

In the next phase of this study (ongoing), we are validating the RECAP score using data linkage between general practice electronic records and national datasets using the primary outcome of hospital admission and secondary outcomes of intensive care unit admission and death. Further details are obtainable from the authors.

Because of the novelty of the disease and the urgency of the research question, we decided to place our interim findings in the public domain so as to allow other teams to test and improve the score in parallel with our own continuing research (rather than, as is more commonly the case, seeking to ensure that our own validation study is published first). We welcome offers of collaboration from established research teams.

For peer review only

Figure 1: Study flowchart

See separate file

Figure 2: Summary of RECAP-0 score

See separate file

For peer review only

For peer review only - http://bmjopen.bmj.com/site/about/guidelines.xhtml

Table 1: Results of Delphi panel

Focus of item	Percentage agreeing with item as worded			
	Round 1 (n = 62)	Round 2 (n = 53)	Round 3 (n = 56)	Round 4 (n = 51)
Pulse rate (wording of item)	73%	70%	**88%**	
Cut-off values for each category	71%	77%	**84%**	
Temperature (wording)	58%	75%	**89%**	
Cut-off values	55%	81%	**89%**	
Duration of temperature* (wording)	58%			**63%**
Cut-off values				**76%**
Symptoms of fever (e.g. chills, shivers)	29%	75%	**91%**	
Cut-off values	63%	87%	**95%**	
Respiratory rate (wording)	61%	85%	**84%**	
Cut-off values	76%	85%	**91%**	
Shortness of breath (wording)	39%	75%	**79%**	
Cut-off values	44%	79%	**93%**	
Trajectory of breathlessness				**73%**
Cut-off values				**67%**
Oxygen saturation level	65%	79%	**80%**	
Cut-off values	65%	85%	**82%**	
Oxygen saturation level after exercise	-	68%	**75%**	
Cut-off values		85%	**91%**	
Clinical suspicion of hypoxia	47%			
Cut-off values	55%			
Tiredness (wording)	42%	83%	**88%**	
Cut-off values	73%	89%	**84%**	
Muscle pains or aches	66%	-	64%	**82%**
Cut-off values	47%	-	**79%**	
Risk factors (comorbidities)	53%	57%	**86%**	
Cut-off values			**82%**	
Risk factors (demographic e.g. age, ethnicity)			**77%**	
			**86%**	
Indicators of shock (including conscious level, new confusion**, low or no urine output, cold and clammy, mottled skin)	61%	64%	Moved to 'red flag' box to align with national guidance	-
Other red flag symptoms e.g. central chest pain	73%	34%		
Clinical concern	74%	77%	**86%**	

* 'Duration of temperature' was changed to 'duration of symptoms' after focus group discussion, with 8 days seen as the cut-off for clinical concern

** New confusion was subsequently proposed as a separate RECAP item by focus group participants, who did not agree that it should be an automatic 'red flag' sign

For peer review only

Box 1: The clinical course of the deteriorating COVID-19 patient in primary care

Synthesised from our qualitative data, supplemented from published sources

COVID-19 may present in primary care as a viral upper respiratory tract infection (e.g. sore throat), lower respiratory tract infection (e.g. cough, fever and mild dyspnoea), influenza-like illness (with fever, chills, headache and myalgia), or gastro-intestinal illness (with abdominal pains, nausea and diarrhoea).[2 6] Most patients have a relatively mild, self-limiting illness, but an unknown proportion (perhaps 10%) deteriorate, usually in week 2. Certain symptoms common in week 1, such as cough, mild fatigue and anosmia, do not appear to have prognostic significance.[5 40]

It is important to date-stamp the onset of first symptoms.[6] Severe dyspnoea, especially at rest, may indicate progression of lung involvement. The trajectory of dyspnoea is important, as acute respiratory distress syndrome occasionally follows quickly from the onset of breathlessness.[41] Formal scores for assessing dyspnoea severity appear to have a significant false negative rate and should not be used.[20] A careful history, noting what the patient is able to do and what they cannot do today that they could do yesterday, is likely to be more important.[20]

Pulse oximeter readings are extremely useful in assessing unwell patients with COVID-19, so long as the device is reliable (smartphone apps are inherently inaccurate and should not be used[22]) and the patient or a relative is capable and confident to use it. The finger must be warm. Whilst a low oximeter reading is concerning, a normal one should not necessarily reassure, as young fit patients in particular can compensate well in the early stages of deterioration.

So-called silent hypoxia, defined as the development of respiratory failure without the subjective perception of dyspnoea, is a recently-described feature of severe COVID-19 and appears to have a poor prognosis.[40 42-45] Anecdotal accounts suggest that in some patients, silent hypoxia may manifest as profound tiredness, but we could not find published research on this association. New confusion (especially in older patients with comorbidity) was considered by clinicians in our sample to be a poor prognostic sign in COVID-19, but at the time of writing, evidence for this is limited.[46]

COVID-19 lung damage tends to be manifest as a perfusion defect (i.e. difficulty transferring oxygen across the alveolar membrane) rather than a ventilatory defect (difficulty getting air to the alveoli, as in asthma).[41] This may explain why COVID-19 can behave similarly to pneumocystis pneumonia in producing a fall in pulse oximetry reading on exertion (or in the minutes following exertion).[21 47] Because of this, patients with suspected COVID-19 should not be subject to exercise testing unless there is a clinician present if their resting pulse oximetry reading is abnormal (below 96%).

An unwell patient may or may not have COVID-19. An overall assessment is needed using questions relating to (for example) hydration status, dizziness, falls, central chest pain, fall in blood pressure (if the patient has equipment at home), change in mental status (including lethargy, new confusion, difficulty in rousing), central cyanosis (e.g. blue lips), and severe reduction in urine output. For this reason, a standard 'red flag' checklist should be quickly reviewed in all unwell patients.

There are some well-established risk factors for developing COVID-19 and worse outcome (e.g. age, non-white ethnicity, high body mass index and comorbidities including cardiovascular disease, hypertension and active cancer).[48-50] The extent to which these risk factors should be applied to 'load the score' of a patient who appears to have a mild form of the disease is not yet known, especially since shielded patients are the ones for whom a hospital or clinic visit carries most risk.

For peer review only - http://bmjopen.bmj.com/site/about/guidelines.xhtml

Declarations

Ethics approvals and consent to participate

Ethical approval for the patient interviews was obtained from East Midlands – Leicester Central Research Ethics Committee (IRAS Project ID: 283196; REC ref 20/EM0128) on 4th May 2020. Ethical approval for the validation study (including holding the focus groups and building and installing the software on primary care record systems) was obtained from Greater Manchester Research Ethics Committee on 20th May 2020 (IRAS Project ID: 283024; REC ref 20/NW/0266) and subsequent amendments. In accordance with our ethics approvals, all patient participants gave written informed consent and all clinician focus group participants gave verbal consent which was recorded on the video. We sought advice from the chair of our local NHS Research Ethics Committee who considered that the online Delphi panel did not require formal ethical approval; consent from these participants was obtained by email.

Contributorship statement

TG conceptualised the study and oversaw all work packages described in the paper. She led or contributed to the various rapid reviews, recruited participants, led data analysis and summarising, and wrote the paper. PT designed and led the operational and quantitative aspects of the Delphi study and the quantitative anlyses. SW and ALN recruited participants, co-facilitated the focus groups and participated in data analysis and writing up. LH provided assistance with all aspects of the research. MD designed the templates for putting RECAP items on general practice electronic records and participated in focus groups. AR interviewed patient participants and extracted relevant data for this study. DN led rapid reviews on predictive value of signs and symptoms. SdeL and BD contributed to data interpretation and analysis, especially in relation to coding and linkage of electronic data, and design of the validation study. All authors checked and approved the final manuscript.

For peer review only - http://bmjopen.bmj.com/site/about/guidelines.xhtml

Consent for publication

Not applicable.

Availability of data and materials

All relevant data are included in the appendices.

Competing interests

MD's company makes templates for general practice record systems. All other authors declare no competing interests.

Funding statement

The RECAP study was funded primarily from a UK Research and Innovation grant 'Remote By Default' under the COVID-19 Emergency Fund. Additional funding for some staff salaries came from National Institute for Health Research (BRC-1215-20008) and Wellcome Trust (WT104830MA).

Acknowledgements

We thank the patients and clinicians who participated in the study at a particularly busy and stressful time. James Ray gave general advice and suggested the RECAP acronym.

Data sharing

Additional data, including an extensive summary of qualitative findings, is supplied as appendices. Researchers seeking further raw data should contact the corresponding author.

For peer review only - http://bmjopen.bmj.com/site/about/guidelines.xhtml

Patient and public involvement statement

Several of the research team are clinicians who developed COVID-19 and thus bring lived experience of the condition. The study was overseen by an independent advisory group with a lay chair and a separate patient advisory group consisting of people from diverse social and ethnic backgrounds who have had COVID-19; this group had input to the study design. The experience of patients was also captured systematically through patient interviews as an integral component of the research, and this led to amendments to the RECAP score. Some patient participants were recruited by snowballing from other patient participants. Results will be disseminated to all patient participants by emailing or posting a lay summary.

For peer review only

For peer review only - http://bmjopen.bmj.com/site/about/guidelines.xhtml

Additional material

Filename	Additional File 1 Focus group prompts
File format	Word (.docx)
Title of data	Focus group prompt questions
Description of data	Focus group prompt questions

Filename	Additional File 2 Qualitative comments
File format	Word (.docx)
Title of data	Qualitative comments during Delphi process
Description of data	Examples of qualitative comments made in Delphi exercise

Filename	Additional File 3 Summary of vignette study
File format	Word (.docx)
Title of data	Summary of vignette study
Description of data	Methods and findings from vignette sub-study of Delphi process

Filename	Additional File 4 RECAP-0 full
File format	Word (.docx)
Title of data	Full version of RECAP-0 score
Description of data	RECAP-v0 score with full detail of how to complete each item

For peer review only - http://bmjopen.bmj.com/site/about/guidelines.xhtml

References

1. Wu Z, McGoogan JM. Characteristics of and important lessons from the coronavirus disease 2019 (COVID-19) outbreak in China: summary of a report of 72 314 cases from the Chinese Center for Disease Control and Prevention. *JAMA* 2020;323:1239-42.
2. Cohen P, Blau J. Coronavirus disease 2019 (COVID-19): Outpatient management in adults. UpToDate: Wolters Kluwer 2020 (Last updated 28th May). Accessed 31st May 2020 at https://www.uptodate.com/contents/coronavirus-disease-2019-covid-19-outpatient-management-in-adults.
3. Sperrin M, Grant SW, Peek N. Prediction models for diagnosis and prognosis in Covid-19: British Medical Journal Publishing Group, 2020.
4. Wynants L, Van Calster B, Bonten MM, et al. Prediction models for diagnosis and prognosis of covid-19 infection: systematic review and critical appraisal. *bmj* 2020;369
5. Nunan D, Brassey J, Mahtani K, et al. COVID-19 Signs and Symptoms Tracker. Oxford: Centre for Evidence-Based Medicine 2020. Accessed 10th June 2020 at https://www.cebm.net/covid-19/covid-19-signs-and-symptoms-tracker/.
6. Greenhalgh T, Koh GCH, Car J. Covid-19: a remote assessment in primary care. *Bmj* 2020;368:m1182. doi: 10.1136/bmj.m1182 [published Online First: 2020/03/28]
7. Royal College of General Practitioners. Using the National Early Warning Score (NEWS2) in Primary Care. London: RCGP 2020. Accessed 8th April 2020 at https://elearning.rcgp.org.uk/mod/page/view.php?id=10568.
8. Royal College of Physicians (UK). National Early Warning Score (NEWS) 2. London: RCP 2017. Accessed 5th April 2020 at https://www.rcplondon.ac.uk/projects/outputs/national-early-warning-score-news-2.
9. National Institute for Health and Clincial Excellence. COVID-19 rapid guideline: managing suspected or confirmed pneumonia in adults in the community. London: NICE 2020. Accessed 4th April 2020 at https://www.nice.org.uk/guidance/ng165.
10. Carr E, Bendayan R, Bean D, et al. Supplementing the National Early Warning Score (NEWS2) for anticipating early deterioration among patients with COVID-19 infection. *medRxiv* 2020
11. Shea BJ, Reeves BC, Wells G, et al. AMSTAR 2: a critical appraisal tool for systematic reviews that include randomised or non-randomised studies of healthcare interventions, or both. *bmj* 2017;358:j4008.
12. Boulkedid R, Abdoul H, Loustau M, et al. Using and reporting the Delphi method for selecting healthcare quality indicators: a systematic review. *PloS one* 2011;6(6)
13. Khodyakov D, Chen C. Response Changes in Delphi Processes: Why Is It Important to Provide High-Quality Feedback to Delphi Participants? *Journal of Clinical Epidemiology* 2020
14. Belton I, MacDonald A, Wright G, et al. Improving the practical application of the Delphi method in group-based judgment: A six-step prescription for a well-founded and defensible process. *Technological Forecasting and Social Change* 2019;147:72-82.
15. Kitzinger J. The methodology of focus groups: the importance of interaction between research participants. *Sociology of health & illness* 1994;16(1):103-21.
16. Wilkinson S. Focus group methodology: a review. *International journal of social research methodology* 1998;1(3):181-203.
17. Abrams KM, Gaiser TJ. Online focus groups. *The SAGE handbook of online research methods* 2017:435-50.
18. Ritchie J, Spencer L. Qualitative data analysis for applied policy research. Analyzing qualitative data: Routledge 2002:187-208.
19. Greenhalgh T, Kotze K, Van Der Westhuizen H-M. Are there any evidence-based ways of assessing dyspnoea (breathlessness) by telephone or video? : Oxford COVID-19 Evidence Service Rapid Review. 30th March 2020. Accessed 1.4.20 at https://www.cebm.net/covid-19/are-there-any-evidence-based-ways-of-assessing-dyspnoea-breathlessness-by-telephone-or-video/.
20. Greenhalgh T. Should the Roth score be used in the remote assessment of breathlessness? Oxford: Oxford COVID-19 Evidence Service. Accessed 30th May 2020 at https://www.cebm.net/covid-19/roth-score-not-recommended-to-assess-breathlessness-over-the-phone/ 2020.
21. Greenhalgh T, Javid B, Knight M, et al. What is the efficacy and safety of rapid exercise tests for exertional desaturation in covid-19? Oxford: Oxford COVID-19 Evidence Service. Accessed 30th May 2020 at

For peer review only - http://bmjopen.bmj.com/site/about/guidelines.xhtml

https://www.cebm.net/covid-19/what-is-the-efficacy-and-safety-of-rapid-exercise-tests-for-exertional-desaturation-in-covid-19/. 2020.
22. Tarassenko L, Greenhalgh T. Should smartphone apps be used as oximeters? Oxford: Oxford COVID-19 Evidence Service. Accessed 30th May 2020 at https://www.cebm.net/covid-19/question-should-smartphone-apps-be-used-as-oximeters-answer-no// 2020.
23. Tarassenko L, McManus R, Greenhalgh T. Is the 'Lifelight' app adequately validated for blood pressure measurement? Oxford: Oxford COVID-19 Evidence Service. Accessed 30th May 2020 at https://www.cebm.net/covid-19/question-is-the-lifelight-app-adequately-validated-for-blood-pressure-measurement-answer-no/. 2020.
24. Chorin E, Padegimas A, Havakuk O, et al. Assessment of Respiratory Distress by the Roth Score. *Clinical cardiology* 2016;39(11):636-39.
25. Guan W-j, Ni Z-y, Hu Y, et al. Clinical characteristics of coronavirus disease 2019 in China. *New England Journal of Medicine* 2020
26. Menni C, Valdes AM, Freidin MB, et al. Real-time tracking of self-reported symptoms to predict potential COVID-19. *Nature medicine* 2020:1-4.
27. Gerry S, Bonnici T, Birks J, et al. Early warning scores for detecting deterioration in adult hospital patients: systematic review and critical appraisal of methodology. *bmj* 2020;369:m1501.
28. Inada-Kim M. NEWS 2: An opportunity to standardise the management of deterioration and sepsis *BMJ (blog)* 2018;8th February. Accessed 4th April 2020 at http://blogs.bmj.com/bmj/2018/02/08/news-2-an-opportunity-to-standardise-the-management-of-deterioration-and-sepsis/
29. National Institute for Health and Clincial Excellence. National Early Warning Score systems that alert to deteriorating adult patients in hospital (Medtech innovation briefing 205). LOndon: NICE 2020.
30. National Institute for Health and Clincial Excellence. COVID-19 rapid guideline: critical care in adults. Accessed 4th April 2020 at https://www.nice.org.uk/guidance/ng159/resources/covid19-rapid-guideline-critical-care-pdf-66141848681413. London: NICE 2020.
31. Patel R, Nugawela MD, Edwards HB, et al. Can early warning scores identify deteriorating patients in pre-hospital settings? A systematic review. *Resuscitation* 2018;132:101-11.
32. Grant S, Crimmons K. Limitations of track and trigger systems and the National Early Warning Score. Part 2: sensitivity versus specificity. *Br J Nurs* 2018;27(12):705-10. doi: 10.12968/bjon.2018.27.12.705 [published Online First: 2018/06/29]
33. Grant S. Limitations of track and trigger systems and the National Early Warning Score. Part 3: cultural and behavioural factors. *Br J Nurs* 2019;28(4):234-41. doi: 10.12968/bjon.2019.28.4.234 [published Online First: 2019/02/28]
34. Grant S. Limitations of track and trigger systems and the National Early Warning Score. Part 1: areas of contention. *Br J Nurs* 2018;27(11):624-31. doi: 10.12968/bjon.2018.27.11.624 [published Online First: 2018/06/13]
35. Pullyblank A, Tavaré A, Little H, et al. Implementation of the National Early Warning Score in patients with suspicion of sepsis: evaluation of a system-wide quality improvement project. *British Journal of General Practice* 2020;70(695):e381-e88.
36. Roland DT, Rowland A, Cotterill S, et al. Fine balance between pragmatism and rigidness in system approaches to acute care. *Bmj* 2019;367:l6226.
37. Scott LJ, Redmond NM, Garrett J, et al. Distributions of the National Early Warning Score (NEWS) across a healthcare system following a large-scale roll-out. *Emerg Med J* 2019;36(5):287-92.
38. Stow D, Barker RO, Matthews FE, et al. National Early Warning Scores (NEWS/NEWS2) and COVID-19 deaths in care homes: a longitudinal ecological study. *medRxiv* 2020
39. Collins GS, Reitsma JB, Altman DG, et al. Transparent reporting of a multivariable prediction model for individual prognosis or diagnosis (TRIPOD): the TRIPOD statement. *British Journal of Surgery* 2015;102(3):148-58.
40. Chen T, Wu D, Chen H, et al. Clinical characteristics of 113 deceased patients with coronavirus disease 2019: retrospective study. *Bmj* 2020;368
41. Gattinoni L, Chiumello D, Caironi P, et al. COVID-19 pneumonia: different respiratory treatments for different phenotypes? *Intensive Care Medicine* 2020:1.
42. Ottestad W, Seim M, Mæhlen JO. COVID-19 with silent hypoxemia. *Tidsskrift for Den norske legeforening* 2020
43. Wilkerson RG, Adler JD, Shah NG, et al. Silent hypoxia: A harbinger of clinical deterioration in patients with COVID-19. *The American Journal of Emergency Medicine* 2020

For peer review only

For peer review only - http://bmjopen.bmj.com/site/about/guidelines.xhtml

44. Chauhan V, Galwankar SC, Yellapu V, et al. State of the globe: The trials and tribulations of the COVID-19 pandemic: Separated but together, telemedicine revolution, frontline struggle against "Silent Hypoxia," the relentless search for novel therapeutics and vaccines, and the daunting prospect of "COVIFLU". *Journal of Global Infectious Diseases* 2020;12(2):39.
45. Xie J, Tong Z, Guan X, et al. Critical care crisis and some recommendations during the COVID-19 epidemic in China. *Intensive care medicine* 2020:1-4.
46. Niazkar HR, Zibaee B, Nasimi A, et al. The neurological manifestations of COVID-19: a review article. *Neurological Sciences* 2020:1.
47. Smith D, Wyatt J, McLuckie A, et al. Severe exercise hypoxaemia with normal or near normal X-rays: a feature of Pneumocystis carinii infection. *The Lancet* 1988;332(8619):1049-51.
48. Zheng Z, Peng F, Xu B, et al. Risk factors of critical & mortal COVID-19 cases: A systematic literature review and meta-analysis. *Journal of Infection* 2020
49. Public Health England. Disparities in the risk and outcomes of COVID-19. . London: PHE Publications. Gateway number: GW-1311. Accessed 8th June 2020 at https://assets.publishing.service.gov.uk/government/uploads/system/uploads/attachment_data/file/890258/disparities_review.pdf 2020.
50. de Lusignan S, Dorward J, Correa A, et al. Risk factors for SARS-CoV-2 among patients in the Oxford Royal College of General Practitioners Research and Surveillance Centre primary care network: a cross-sectional study. *The Lancet Infectious Diseases* 2020

For peer review only

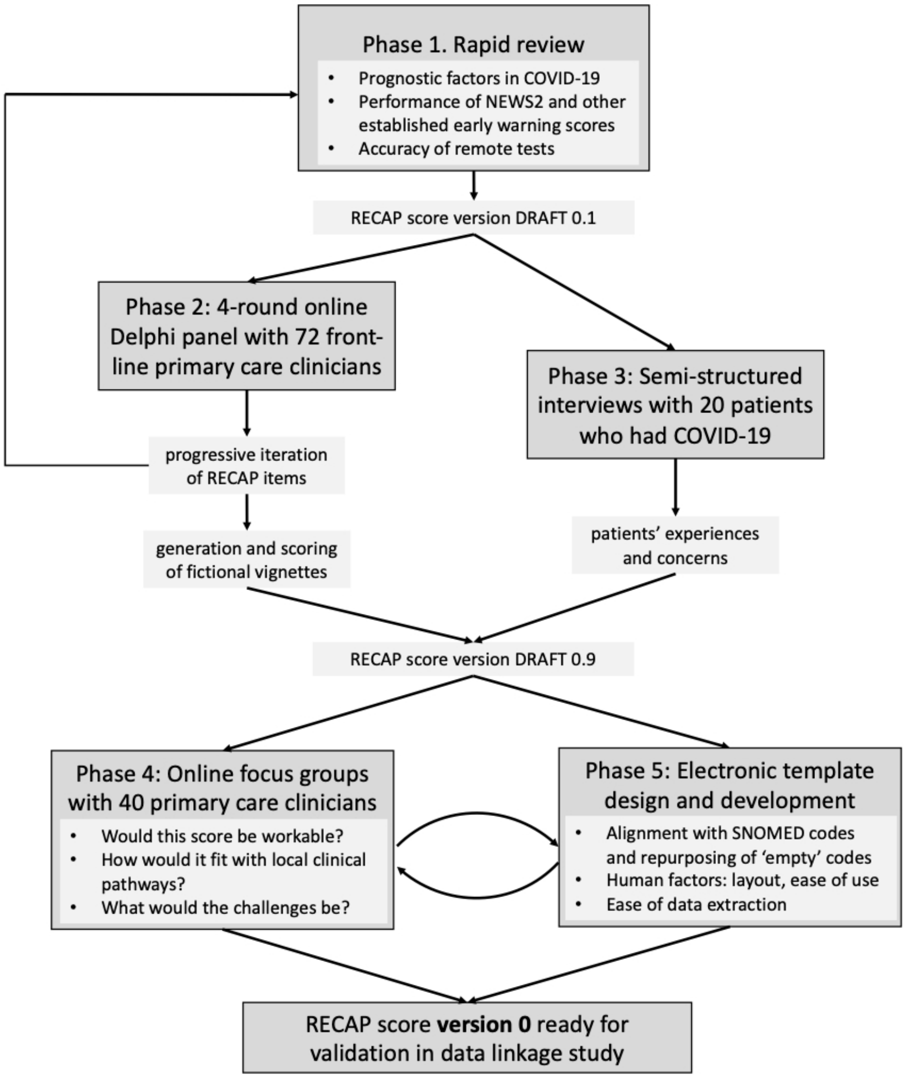

Figure 1: Study flowchart

RED ALERT CRITERIA: If patients have any of the following, consider 999

Adapted from national primary care covid-19 recommendations

Shock or peripheral shutdown

- Reduced level of consciousness
- Extremities – cold and clammy to touch
- Pallor – skin is mottled, ashen, blue or very pale
- Reduced urine output – little or no urine in last 24 hours

Severe breathlessness

- Rapid, significant deterioration in breathing in last hour
- New breathlessness at rest
- Newly unable to complete sentences
- Sudden onset of breathlessness

Other red flags which may be non-covid-19 related e.g.

- Severe central chest pain
- Collapse

RECAP-V0 SCORE FOR PATIENTS WHO DO NOT HAVE RED ALERT SYMPTOMS OR SIGNS

		Score 0	Score 1	Score 2	Score 3 => refer urgently	Score
1	Heart rate (per minute) *(if heart rate not available, score 1)*	51-90	41-50 or 91-110 or missing data	111-130	≤ 40 OR > 130, if unexplained	
2a	Shortness of breath	Not breathless at all	Breathless on moderate exertion e.g. walking room to room	Breathless on mild exertion e.g. getting out of a chair	Severe breathing difficulty; can't complete sentences at rest	Highest of 2a or 2b
2b	<u>or</u> Respiratory rate (per minute)	12-20	21-24	9-11 or 25-29	8 or less, or 30 or more	
3	Trajectory of breathlessness	Same or better than yesterday	Breathless, worse than yesterday	-	Significant deterioration in last hour	
4a	Oxygen saturation at rest	96% or above	95% (don't do 40-step test unsupervised)	94% (don't do 40-step test unsupervised)	93% or below (don't do 40-step test)	Highest of 4a, 4b & 4c
4b	<u>or</u> Saturation after 40 steps	Fall of 0-1%	-	Fall of 2%	Fall of 3% or more	
4c	<u>or</u> Profound tiredness or fatigue	None or mild	Noticeably more tired doing usual activities	Struggling to get out of bed	Unable to speak because of tiredness	
5a	Temperature	≤ 38 °C	38.1-39 °C	> 39 °C or < 35 °C	-	Highest of 5a or 5b
5b	<u>or</u> Feeling feverish with shivers	None	Feverish or chills	Uncontrollable shivering	-	
6	Time from first symptom (days)	7 or fewer	8 or more	-		
7	Muscle aches	None or mild	Moderate	Severe		
8	Cognitive decline	No	Less mentally alert than usual	New and worsening confusion	Reduced level of consciousness	
9	On COVID-19 shielded list (or has been inadvertently left off it)?	No	Yes	-	-	
10	Other risk factors for poor outcome? e.g. age, ethnicity	0-2	3 or more	-	-	
TOTAL						

RECAP score	Meaning	Recommended action
7 or more total <u>or</u> 3 on any item <u>or</u> extremely high level of clinical concern	HIGH RISK	Consider urgent referral
4-6 or more total <u>or</u> high level of clinical concern	MODERATE RISK	See in hot hub or virtual ward, or arrange home visit
0-3 total	LOW RISK	Advice and monitor at home

Figure 2: Summary of RECAP score

Appendix A1: Focus group prompt questions

1. How has your **experience managing COVID-19** been? **How do you make decisions about who to refer on**?
2. Do you **use any clinical decision score**? If so, which one?
3. What would a warning score **need to do for you**?
4. **Which patients does it need to select** for you?
5. How was your overall **experience using RECAP**?
6. Which were the main **positive aspects of RECAP**?
7. Did you have **any difficulties using RECAP**? Which?
    a. In particular, how does clinical uncertainty (i.e. difficulties assessing objective measures, such as HR) impact the application of RECAP?
    b. How do remote communication barriers (i.e. language, no video, poor phone line) impact the application of RECAP?
    c. Was time a problem? (i.e. Is it short enough to be useful in clinical practice?)
8. **How do you think these problems/difficulties could be eliminated or overcome**? Can you anticipate any possible solutions?
9. How do your **local and structural arrangements** influence your ability to apply RECAP?
10. Do you feel you need any additional information or training on how to use RECAP?
11. Is there anything else you want to add or feel we should have spoken about?
12. Demonstration of RECAP template using screen share.

***

Other possible questions to be discussed:

Are there any other ways to achieve #3?

Does RECAP seem capable to select those who would, or not benefit from oxygen therapy or heparin?

For peer review only - http://bmjopen.bmj.com/site/about/guidelines.xhtml

Appendix A2: Qualitative comments during Delphi process

Pulse rate

"We have noted that about 50% of our patients have BP monitors at home, which has been very helpful in our video consultations. In addition, we send out sats bus out to those patients we are concerned about, so have been able to include the PR [pulse rate] even in patients without." [round 1]

"I think it [pulse rate] is really helpful in identifying sick young fit people who are otherwise compensating quite well and may have normal resting sats and a slightly high RR [respiratory rate] and significant fatigue and look ok. Less helpful in older adults unless very high. I have no idea of its predictive value for outcomes though." [round 1]

"We have seen a lot of tachycardia in patients with milder symptoms so I think 2 rather than 3 is an appropriate score" [round 2]

"A bradycardia below 40 is an emergency whether or not a patient is on a B-blocker so the advice to adjust by 10 should not apply. Just because a patient uses a B blocker doesn't mean they can tolerate a low cardiac output when septic/unwell." [round 3]

"They [pulse rate cut-offs] don't align with London guidance"

Temperature and symptoms of fever (chills, shivers)

"In my (pre-Covid) experience, patients are excellent at recognising rigors as being different from a normal high temperature." [round 1]

"Temp 37.8 by NHS 111 is I think more about meeting clinical criteria for isolation, rather than because of any clinical utility. I think your cut offs are sensible." [round 2]

"'use peak temperature before paracetamol' implies a level of control that the clinician won't have. Why not 'highest recorded temperature in the last 24 hours' or similar?" [round 3]

"I would just score 1 for a temperature, rather than the height of the temperature" [round 3]

"If you mean 'rigors' then the orange box should have the word 'rigors' in it. Shivers and chills aren't rigors (I've had both). 'Feverish or chills with uncontrollable shivering' perhaps is closer to the implied meaning." [round 3]

"I suspect that chills is a greater reflection of malaise and temperature than the height of pyrexia and therefore reflects greater illness (but I'm guessing!)." [round 3]

Respiratory rate

"We have found RR [respiratory rate] as a very helpful tool with our video consultations and are using it to determine which patients require home visit/sats bus/hub attendance, with the cut off of more than 24 and less than 9. We are requiring RR for all referrals to our hot hub." [round 1]

"Below 9 to score two- no way! That's a 999 emergency. I have MRCEM and was an intensivist for several years. A low resp rate scares me a lot more than a high one. A low RR tells you the patient is peri-arrest. A high RR is worrying but tells you that the patient is - for the moment - responding appropriately to their respiratory

For peer review only

or metabolic problem. A low RR tells you that they are no longer able to do so and are about to die without intervention (some exceptions, of course, e.g. BZD plus alcohol OD when people often maintain a scarily low RR without apparent significant compromise - but not relevant here)." [round 2]

"I would amend to 2 = 25-29 and score 3 if greater than or equal to 30, to align with CURB and other scores. A RR of 30 is pretty rare and severe." [round 2]

"Almost certainly a good sign but worried about its feasibility" [round 3]

Breathlessness and silent hypoxia

"We have seen many patients with silent hypoxia. These patients have tended to be extremely fatigued. Would you consider including a normal RR but with extreme fatigue as a score 3?" [round 1]

"In practice we have noticed that patients are not articulating their breathlessness well - asking them about how their symptoms (both breathlessness and fatigue) are affecting their usual activities has been key - struggling to get out of bed, for example, is often a very significant change, for others it's not being able to manage the hoovering, for all it's changes to their usual activities and routine" [round 1]

"Would be good to have something to try and capture the silent hypoxics - such as feeling more fatigued and exhausted on exertion? Which I have had patients describe? Or a feeling that breathing is laboured but not breathless. Like at altitude." [round 1]

"Persistent, progressive SOB is a red flag" [round 1]

"Listening to friends (sadly several) who have suffered with COVID and even been to ICU, lot of them were that tired that they could not even speak." [round 2]

"Maybe 2 and 3 are too close together? How about 0: as it is 1: new breathless on moderate exertion eg up stairs 2: Breathless on mild exertion eg walking across room. 3: unable to complete sentences or severe difficultly breathing" [round 2]

"Struggling to get out of bed might not be that unusual for some people!" [round 2]

Illness trajectory

"The patients I've been worried about tend to be pyrexial >38 in week 2" [round 1]

"From the various narratives on the illness I wonder about days since symptoms began - it seems that patients in the first 7 days of symptoms may be less likely to deteriorate, whilst those whose symptoms go on after this probably need assessment. Careful assessment of confusion feels important - for my phone calls I am spending time talking to patients to assess for any level of confusion. This for me is a 'sign of hypoxia' - but that is not flagged clearly enough in the above for my liking." [round 1]

"[Tiredness] varies so much on stage of illness. Those with mild symptoms all c/o feeling very tired in the first 5 days, often not able to get out of bed for a day or two. But can manage their ADL [activities of daily living]. Worsening fatigue around day 7-10, having initially been feeling better, is a much worse prognostic feature in my limited experience." [round 1]

For peer review only

Pulse oximetry and exertional desaturation

"I think the walk test is really important." [round 1]

"Validated scoring system already in place I ask patients to walk 40 steps and then check by video with hand in their chest easier to see and count and sees how they are on exertion in comparison to rest ." [round 1]

"Until we know more I'm not sure that we can leave patients with pre-existing normal lungs at home with a score of 93%- this may be the best early warning that we can find." [round 2]

"Always a problem what to do with chronic lung disease. 6% [correction for chronic lung disease] is quite steep (since obviously depends on degree of chronic hypoxia) but I don't have any more rational suggestion." [round 2]

"NB: pan-London respiratory guidance decided not to specify a number for desaturations in latest draft. I had to miss that meeting so not sure about the reasoning, but I think it was something along the lines of needing to use clinical judgement. Personally, I think 'clinical judgement' needs some guidance as this is a new disease entity, so strongly support having specific numbers on this to guide clinicians." [round 2]

Muscle aches

"This is interesting. I just have not spoken to or seen many people who complain of severe muscle pains at all. This might be because I am seeing people (in the community as a GP) who have mild to moderate disease, and those with really severe disease with severe myalgia bypass their GPs and go straight to hospital. If hospital colleagues are saying it does seem to predict severity then leave it in." [round 4]

"muscle pain has been very common in our unwell patients" [round 4]

Red flag signs

"Central cyanosis needs 999." [round 1; 30 similar comments]

Comorbidities and demographic risk factors

"3 for 61yo male with BMI of 32 is not equal to active cancer, immunosuppressed and high fever in week 2?" [round 2]

"If the other questions don't raise concerns then i don't want to send thousands of obese diabetic hypertensives with mild disease for assessment - I think they need to be given specific advice that they are at risk of becoming more unwell quickly - so please call back if any concerns" [round 2]

"I think the cut offs are perfectly rational, but the comorbidities are complicated and hypertension in particular." [round 2]

"All patients on shielded list and having 3 risk factors will score 4 meeting the criteria for F2F assessment even if they have no concerning clinical features. This means that any non-white male patient over 65 and on the shielding list needs a F2F assessment before we even start with the clinical assessment, which is not appropriate. So I think the scoring levels need to be reconsidered and reduced for this or 6b. My inclination would be to reduce this to a 1 if on list and leave 6b as it is." [round 3]

For peer review only

"I would have the top score as '4 or more'. A fit 68yr old Asian male with no other illnesses, or a white male with either well controlled hypertension or diabetes scoring 2 feels a little high." [round 3]

Other comments

"I don't trust China data" [round 1]

"I think you have covered the important things. The key here is to assess physiology. I think history is less important. The patients who become very unwell have a large systemic inflammatory response or severe pneumonia/pneumonitis so we need to focus on identify when people have SIRS or significant respiratory compromise." [round 1]

"I am very concerned about the application of clinical scores to patients that don't capture the individuality of patient circumstances - particularly if they are to be used to inform important decisions such as admission to hospital or deciding that a patient is in need of end of life care. There is always a danger that scores will be used to reduce and resolve these hugely complex issues - and are very clinician dependent." [round 1]

"I am not clear on the aim of the scoring system, and whether it acts as 'protocol' rather than a decision augmenting tool.. I hope you don't mind my honesty as I would like to help produce a simple, user-friendly diagnostic and prognostic tool.. but I think it needs to augment existing high level clinical skills rather than be another protocol to fill, creating over-reliance on this tool and cognitive distraction in the field. " [round 2]

"a really important part of our assessment in primary care is social circumstance and / or ability to mobilise community services. Lack of either of these can lead to admission too - while they may not be clinical "red flags" they may be worth including somehow as they contribute to the rationale for the decision to admit to hospital (and capture a little about the person being admitted)" [round 2]

"An observation. I realise that it is an unavoidable consequence of remote assessment but if you have a device to measure sats or pulse rate, you have 'more chance' of having a higher overall score." [round 3]

"There are too many questions if all included, so it is too easy to get a high score - need to reduce the number - could aim for 8 questions as in NEWS2? Would it be worth considering a table top exercise before sending out for initial testing? (eg give some scenarios & decide if admit or not & then do score to see if confirms decision or not?)." [round 3]

For peer review only

Appendix A3: Summary of vignette study

We used five vignettes, deliberately presented as 'grey cases' with considerable clinical, technical and social uncertainty (missing data, vague symptoms, potentially irrelevant material). In the first round of vignettes, we asked participants to score each item on the RECAP instrument, calculate the RECAP score and then say what they would recommend for the patient and whether the score reflected their clinical concern.

The 51 responses to this round showed surprising variation (e.g. the RECAP score varied from 2 to 16 in one vignette). However, free text comments suggested that the respondents were unsure how to calculate the score from the individual items (uncertainty which to add with 'and' and which to use as alternates with 'or', and whether to count the clinical concern within the RECAP score or separately from it).

We therefore ran a second round of vignettes (using three of the five), and using an automated scoring system. This produced 37 responses and a more consistent response, though still a fairly wide spread. Free text responses suggested that respondents found the vignettes time-consuming and somewhat confusing.

Details analysis is below.

VIGNETTE 1: Mrs Rahman

> *Mrs Rahman (South Asian ethnicity) is 78; she lives with her extended family who are concerned about her. She has had a dry cough and has felt hot for 6 days. Her appetite is poor (she has apparently lost her sense of taste). She spends most of her time in bed and shows no signs of getting better. When you try speak to her, you have no common language but her daughter-in-law says she is not short of breath, just very tired. She says her mother-in-law gets up to go to the toilet, and comes downstairs once a day to watch a TV programme. She says she doesn't look blue or pale. She's been hot and sweaty but has not had uncontrollable shivering, but she is aching quite badly in her back and down her legs, and is very worried. Mrs Rahman has well-controlled type 2 diabetes (on metformin). Her BMI is 27 and a recent BP was 130/85. She had stage 2 breast cancer 20 years ago but no recurrence. The family do not have a thermometer, blood pressure machine, oximeter or other equipment, and nobody has access to a video application.*

Scoring in round 1 (51 responses):

For peer review only

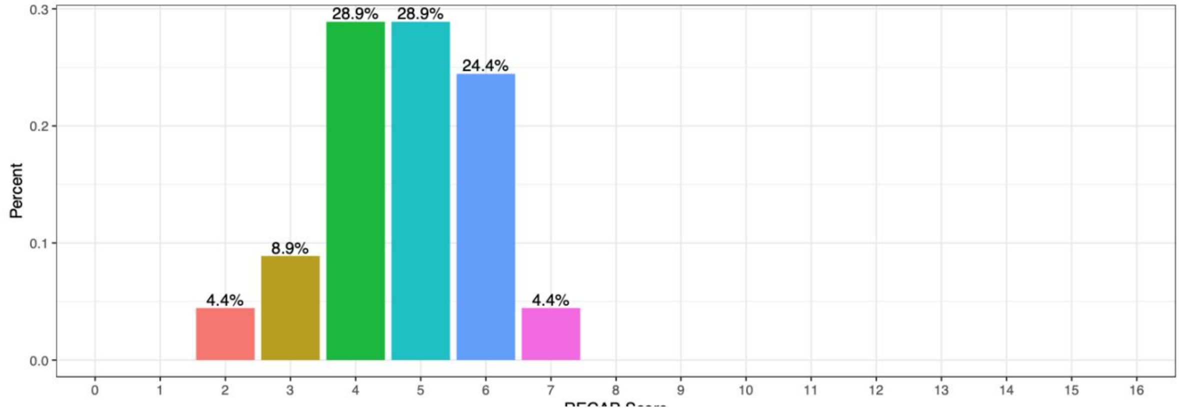

Scoring in round 2 (37 responses)

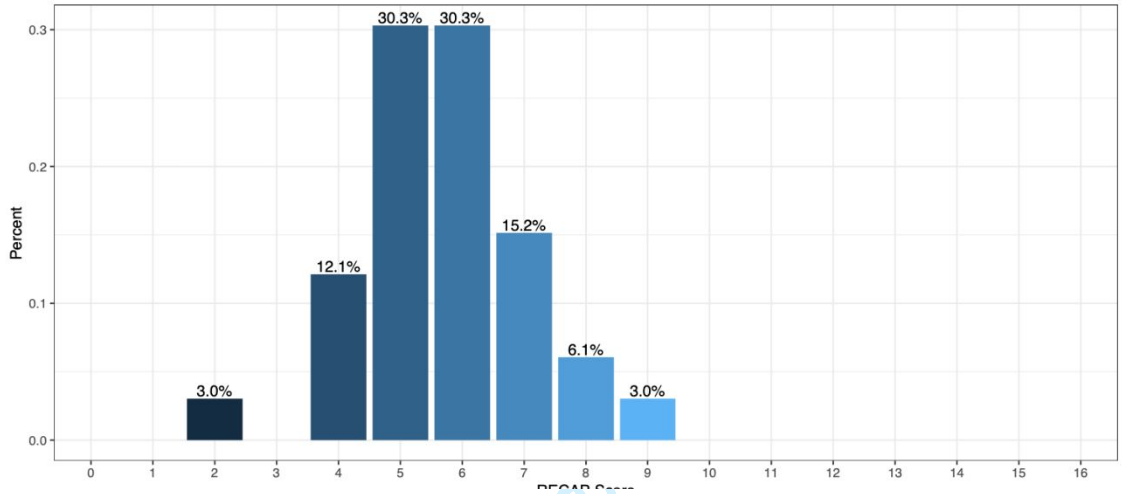

In sum, 82% of the first sample and 72% of the second sample would see in hot hub. 13% of first sample and 3% of second sample would reassure. Interestingly, 4% of the first sample and 24% of second sample would refer to hospital.

Responses to individual items showed that the main ones accounting for variation were

- Tiredness: Noticeably more tired doing usual activities (70%) versus struggling to get out of bed (30%)
- Fever: None (10%), feverish or chills (87%), feverish or chills with uncontrolled shivering (3%)
- Duration of temperature: not applicable (9%), fewer than 7 days (91%)
- Muscle aches: none (3%), moderate (61%), severe (36%)
- Is patient on shielded list? Yes 6%, no 94%.
- Risk factors for poor outcome? No 9%, yes 91%.
- Clinical concern: low 3%, moderate 70%, high 27%.
- Did RECAP reflect clinical concern? Yes 90% No 10%
- What would you do? Reassure 3%, see in hot hub 91%, refer urgently 6%

Qualitative comments:

Many said can't remember what the score is.

"It effectively has a bunch of 'not assessed' elements though, so the risk is that they cannot contribute to the score because missing, but missing does not equal 'not present' if there is a communication barrier (language but also no video or a poor phone line, or an inarticulate person who struggles to explain what they feel etc). I think it delivers the 'right' outcome though in the sense of face to face community review."

For peer review only - http://bmjopen.bmj.com/site/about/guidelines.xhtml

"Not clear how to score if no HR available Q4b) states feverish with shivers but then the score says "feverish or chills" scores 1 point... Q6b) not clear if I would have counted controlled hypertension as one factor, and also if I would use the BP cut off for diabetes in giving a point for diastolic >80... Scored 6 and then gave additional points for moderate worry = 8, which would result in urgent referral but I think I would have seen this patient in a hot site rather than admitting."

"Seeing her in the Red COVID Assessment Unit would feel right. Then we could assess parameters and see what her obs are."

Conclusion: The early warning score would prompt almost all GPs to assess this patient further. This seems appropriate given the multiple risk factors, missing data and language barrier.

VIGNETTE 2: Mr Jamieson

Mr Jamieson (African-Caribbean ethnicity) calls you urgently; he is 57 and lives with his wife. He has been unwell for 9 days, but only started feeling really bad yesterday. He has absolutely no energy and finds it hard to breathe, but is wondering if that's due to his asthma (for which he normally takes a Ventolin inhaler). He isn't out of breath at rest but was very short of breath brushing his teeth this evening, whereas this morning he could do that without a problem. He doesn't sound out of breath on the phone to you, and is able to complete sentences; an attempt at making a video connection fails. He says his peak flow rate is 425 (predicted for age 510; last asthma check 450). He forced himself to get up this morning, and has spent all day in a chair, finding it a struggle even to get to the toilet. He has a BP machine because he's on medication (amlodipine) for high blood pressure. Using his machine, his blood pressure today is 120/70 and pulse 105 (recent clinic values were 148/90 and 78). He's taken his temperature with a mouth thermometer he found in the cupboard but it only reads 35 even though he's sweated so much his wife had to change the sheets. When asked about feeling hot with shivering and chills, he says yes he's had all those, quite badly on one occasion. His muscles don't ache. He has sickle cell trait, but was told this would never cause him any problems. His BMI is 33, and a recent check-up showed an eGFR of 80.

Scoring in round 1 (51 responses):

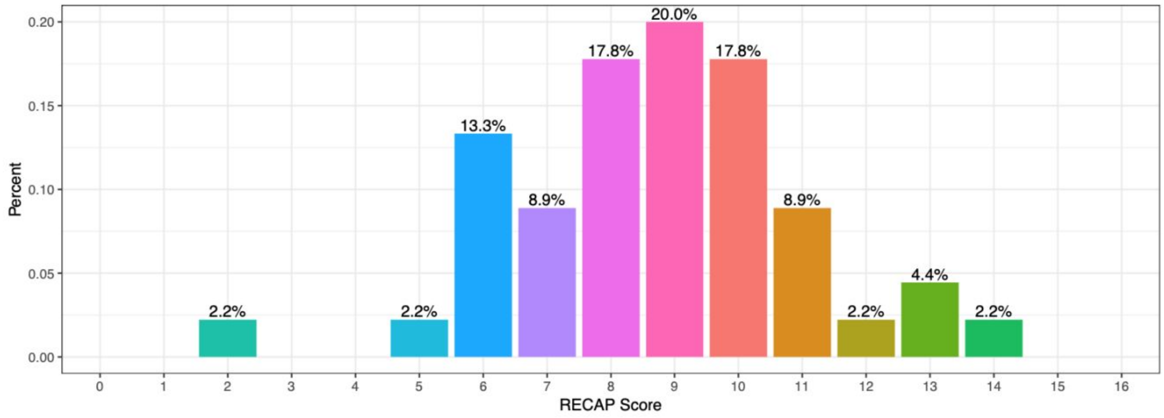

Scoring in round 2 (37 responses):

For peer review only - http://bmjopen.bmj.com/site/about/guidelines.xhtml

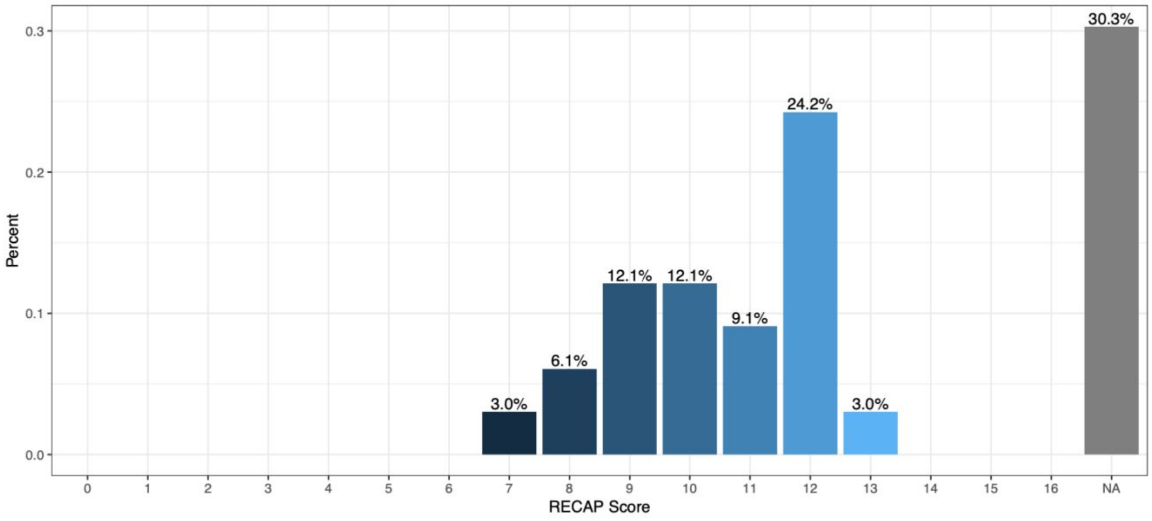

(the N/A column is because the patient has been referred urgently without completing the RECAP score)

In sum, 82% of first sample and 100% of second sample would refer this patient urgently.

Responses to individual items showed that the main discrepancies were

- Tiredness: Noticeably more tired doing usual activities (10%) versus struggling to get out of bed (90%)
- Shielded list yes 77% no 23%
- Duration of temp not applicable 42%, 8 or more days 45%
- Other risk factors for poor outcome 0-2 37%, 3 or more 63%
- Whether to refer urgently without completing RECAP or continue with RECAP
- Level of clinical concern: high 66% extremely high 31%
- Did RECAP capture your level of concern? Yes 77%, no 23% r1 and 90%, 10% r2.

Qualitative comments

Some wanted to ask him more questions about his asthma.

"Rapid deterioration in symptoms over 12 hours - high number of risks. Is at that 9-10 day tipping point for COVID - I would be admitting urgently."

"composite assessment - day 9, acute deterioration SOB, underlying asthma, risk factors. degree of SOB and deterioration is overriding concern"

"I think the profound fatigue is very concerning - I don't know that we are certain whether this is always a sign of silent hypoxia or whether it may just be due to immune response to SARS COV-2. I think I would want to check oxygen saturations, and most likely admit."

"Day 9, SOB brushing his teeth! Likely heading towards ARDS. Wouldn't want to waste time with Hub clinic with this one but would call him an ambulance."

"Although there is an argument (maybe) for see urgently in a hub - if his O2 sats and work of breathing are normal, what is hospital going to do? Xray? Rate of deterioration and duration of illness I think are the critical factors here that tip towards hospital assessment."

[The last comment above illustrates, I think, a GP who is less aware of the possibility of ARDS than the penultimate commenter above – ie RECAP score is budging this GP towards taking more aggressive action than they would otherwise have done – this is good!]

For peer review only - http://bmjopen.bmj.com/site/about/guidelines.xhtml

Conclusion: In every case in round 2 (where we calculated the score automatically), this high-risk patient would be referred urgently. The lower scores on round 1 may have been arithmetical or interpretation errors. Even in round 2, 31% of responders only had “moderate” or “high” clinical concern but would have been prompted to refer urgently.

VIGENTTE 3: Mrs Finlay

> *Mrs Finlay (White Irish ethnicity) is 88 and in a care home. She has multimorbidity (ischaemic heart disease, osteoarthritis, chronic pancreatitis and hypothyroidism) and on multiple medications, but her quality of life is good and she is normally mentally sharp. A recent conversation about ’ceiling of care’ is documented in her medical record: she would like to be referred to hospital if appropriate. Her carers are concerned because after three days of a low-grade fever (37.5 on tympanic thermometer) and worsening breathlessness (which, since this morning, is present even when lying in bed), she has become delirious. Her blood pressure is 110/65 and pulse 120.*

Scoring in round 1 (51 responses)

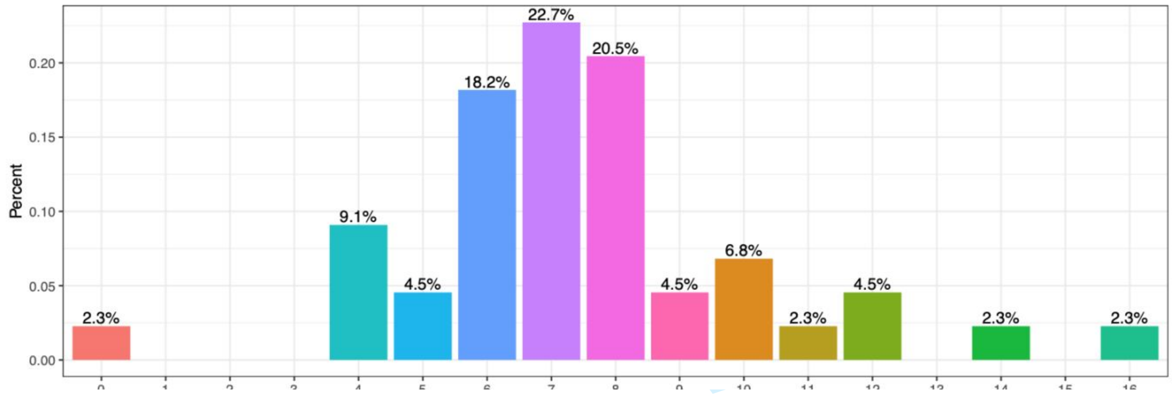

Responses to individual items showed that the main discrepancies were

- Clinical concern: moderate 7%, high 27%, extremely high 66%.
- Did RECAP reflect clinical concern? Yes 64% No 36%
- What would you do? See in hot hub 16%, refer urgently 82%

(Not included in round 2).
Qualitative comments:

“In practice, I would want a more detailed discussion with the carers about whether referral to hospital was “appropriate”. Sounds like it should be, but hard to capture the nuance in a vignette. Unlike the one before or the one after, she is a hospital referral/999 as no point going to a community service if she’s suitable for admission.”

“Actually what I would do if at all possible is eyeball her (by video or in person) to establish if she is really delirious and very SOB. However, if I have to choose from only the 3 options above, I would have to admit. (Note: I am not sure whether you are using ’virtual ward’ to include video consultation - this is not how we use it locally).”

“Given the delirium, and ceiling of care wishes, she needs an urgent work up.”

For peer review only - http://bmjopen.bmj.com/site/about/guidelines.xhtml

"Delirium very difficult to score Also lots of causes of this in elderly eg UTI"

"Trajectory is important here, as is the new delirium and breathlessness at rest. Admit."

"RECAP itself didn't [capture my concern] (only 6, however does have 3 in one area)- however she has a red flag symptom - interestingly because of temperature/ no description of duration-/muscle aches etc, (really a lack of information as May happen in care home residents), the score is lower, but the presence of the red flag (confusion and new sobar) means that the score is less important - so overall the tool Accurately captured my concern."
[this DID capture the concern because RECAP says score 3 in one item → refer urgently]

Conclusion: This was the vignette with the most variation, due to two things: a) some GPs didn't see delirium as a red flag on the front page; b) some felt the delirium wasn't an indication to refer urgently (eg may have been a UTI). But more than 80% of GPs would refer given this story and score.

VIGNETTE 4: Mr Hughes

Mr Hughes is 72 (white British ethnicity) and lives alone. He has been unwell with Covid symptoms for 11 days. He was seen last week in the hot hub clinic because of breathlessness, and supplied with a home oximeter. Today, he feels more breathless than yesterday but says he can still walk around the living room. He's been measuring his saturations daily and the last three days have been 97%, 96%, 96%, but he feels much more tired today than he did yesterday. The oximeter measures his pulse at 98. Using a video app, you measure his respiratory rate at 24. You ask him to take 40 steps around his flat and re-measure his saturation levels. He does this, and says the level is still 96%, but a minute later it has fallen to 94%. His temperature with a mouth thermometer is 37.6 and he hasn't had shivers or chills. He had prostate cancer 10 years ago and was treated for depression last year after his wife died. His BMI is 28.

Scoring in round 1 (51 responses)

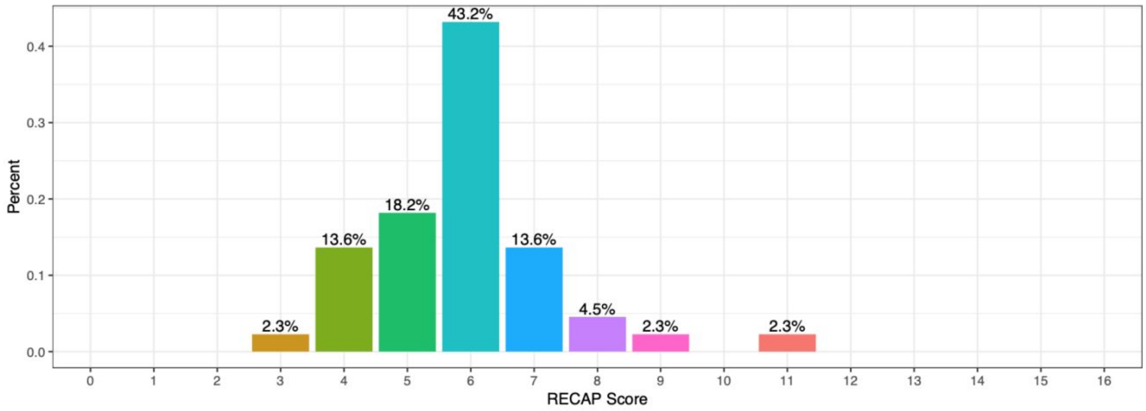

(no round 2 with this case)

In sum, 22% of this sample would refer this patient urgently; all but one responder would review in hot hub or at home.

Responses to individual items showed that the main discrepancies were

- Duration of temp: not applicable 42%, 8 or more days 45%
- Risk factors for poor outcome: 0-2 36%, 3 or more 64%

For peer review only - http://bmjopen.bmj.com/site/about/guidelines.xhtml

- Clinical concern: low 7% moderate 52% high 36% extremely high 5
- Did RECAP reflect clinical concern? Yes 80% No 20%
- What would you do? Reassure and advise 16%, See in hot hub 50%, refer urgently 32%

In sum, there was quite a bit of variation in what GPs subjectively felt was needed, but in all but one case, the patient would have had a hot hub review or hospital referral if score was followed.

Qualitative comments:

"Moderate risk - think the desaturations on walking are main red flag here"

"The post exertional hypoxia is a concern, and the isolation but there are psychosocial factors that might contribute to his malaise. He is not so ill that he needs hospital and a prompt Hub appointment could suffice."

"Deteriorating despite home management, desaturating, and vulnerable living alone. I'm not sure here what the hot hub would add to his assessment. I think he needs ED review."

"He already has a pulse oximeter. He will need daily calls but is probably safe to be managed remotely today."

Conclusion: The spread here was because some GPs did not interpret exertional desaturation as a serious 'red flag' sign. The RECAP score pushed those GPs to reviewing the patient urgently or sending him to hospital. This is what the score is designed to do.

VIGNETTE 5: Mr Liu

Mr Liu is a 37-year old postgraduate student from Taiwan who lives in a shared house of multiple occupation. He's worried because he has been unwell for 10 days and has a cough and a high fever (measured at 39 with a mouth thermometer). He's also finding it hard to catch his breath – something that's been going on for three or four days. He doesn't think it's worse today than yesterday. He doesn't know his housemates well, but he knows two of them have been unwell. He is not registered with your practice so you have no previous records on him, but he tells you he might have been diagnosed with diabetes back in Taiwan, he's not sure. His father had it and he is a bit overweight, though he doesn't know his actual height and weight. He's been lent an oximeter by one of his housemates and taken a reading – his pulse is 115 and saturation 95%. On a video call, you think he looks pale and anxious, and his respiratory rate is 22.

Scoring in round 1 (51 responses)

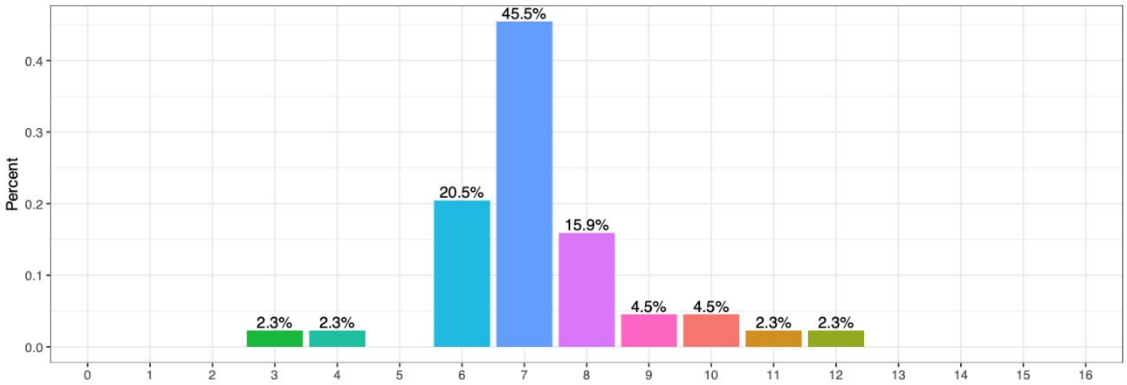

Scoring in round 2 (37 responses)

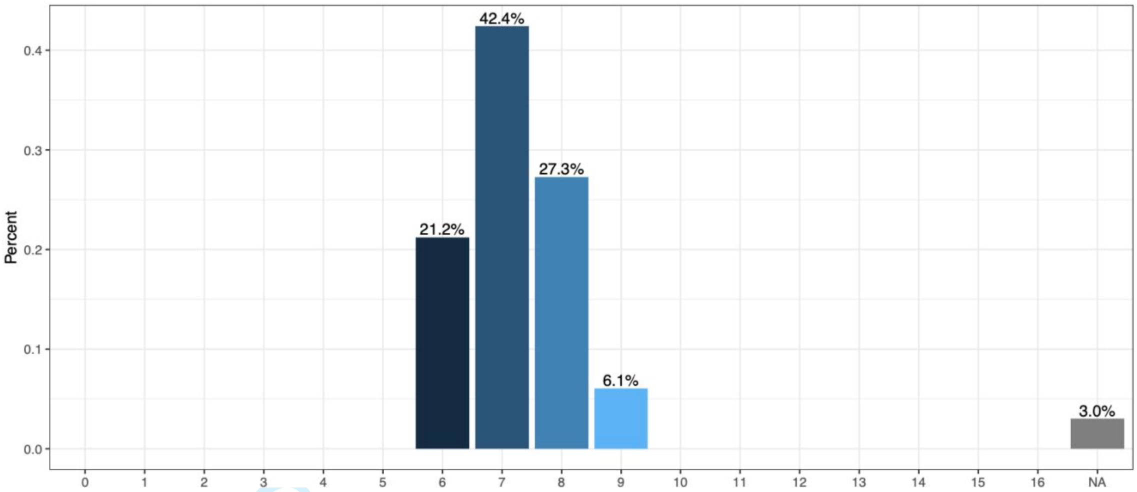

- Level of clinical concern r1 low 2%, moderate 52%, high 39%, extremely high 7%; r2 moderate 3%, high 66%, extremely high 31%.
- What would you do? R1 reassure and advise 5%, see in hot hub 65%, refer urgently 27%; R2 Reassure and advise 18%, See in hot hub 48%, refer urgently 33%
- Did score capture level of clinical concern r1 yes 80% no 20%; r2 90%, 10%.

In sum, 75% of r1 and 80% of r2 would have referred this man urgently.

Qualitative comments

“I think it came up higher than I would have expected.”

“score suggests consider urgent referral - well I’ve considered and am happy for him to be seen in hub. score would be higher still if he has DM and BMI >35 - which Ive not scored - but why he needs to be seen rather than have phone advice.”

“I think there’s a degree of uncertainty around this case. We don’t know the patient and there is some uncertainty regarding his PMH, but the safest thing is to assume he does have diabetes. It can be quite hard to differentiate between anxiety and an unwell patient over video especially, and paleness and tachycardia makes me concerned regarding shock. I would be admitting him.”

“During vignette, I was wondering would I really want to see this patient in a hot hub, to be knower, with Sats of 95 in a fit and well young man; that is worrying enough to me So yes I think overall though not so barn door, I feel direct referral to hospital is appropriate for him mainly given his sats/ duration etc”

Conclusion: This vague case had a number of potentially worrying features and inevitably they were interpreted slightly differently by the responders, but in every case the patient would have either been referred straight to hospital or seen in a hot hub. Once again, the score seems to be doing its job of prompting the GP to act on a sign or symptom they may not be aware is associated with poor prognosis.

For peer review only - http://bmjopen.bmj.com/site/about/guidelines.xhtml

ADDITIONAL COMMENTS

Complaints about layout and not seeing score in front of them.

Complaints about time it took.

--

TG, PT
1.6.20

For peer review only

Appendix A4: Full version of the RECAP-V0 score

REMOTE COVID-19 ASSESSMENT FOR PRIMARY CARE

This is a draft early warning score for use in a remote consultation. It is designed to support assessment of, and communication about, COVID-19 patients at the primary-secondary care interface. It is not intended to replace clinical judgement. It has been produced by professional consensus but has not yet been clinically validated.

If the RECAP score is high, it will not tell you whether to refer for active management (e.g. hospital or hot hub), or arrange palliative care (other factors will of course influence this decision).

RED ALERT CRITERIA: If patients have any of the following, consider 999

Adapted from national primary care covid-19 recommendations

Shock or peripheral shutdown
- New confusion (including delirium)
- Reduced level of consciousness
- Extremities – cold and clammy to touch
- Pallor – skin is mottled, ashen, blue or very pale
- Reduced urine output – little or no urine in last 24 hours

Severe breathlessness
- Rapid, significant deterioration in breathing in last hour
- New breathlessness at rest
- Newly unable to complete sentences
- Sudden onset of breathlessness

Other red flags which may be non-covid-19 related e.g.
- Severe central chest pain
- Collapse

RECAP-V0 SCORE FOR PATIENTS WHO DO NOT HAVE RED ALERT SYMPTOMS OR SIGNS

		Score 0	Score 1	Score 2	Score 3 => refer urgently	Score
1	Heart rate (per minute)<br>*(if heart rate not available, score 1)*	51-90	41-50 or 91-110<br>or missing data	111-130	≤ 40 OR > 130,<br>if unexplained	

Use medically approved device if available, or patient's own. Lower threshold for tachycardia by 10 bpm if beta-blocker or other heart-slowing drug taken in past 24h, but use standard thresholds for bradycardia. Adjust score if known to have physiological bradycardia (e.g. athlete).

		Score 0	Score 1	Score 2	Score 3 => refer urgently	Score
2a	Shortness of breath	Not breathless at all	Breathless on moderate exertion e.g. walking room to room	Breathless on mild exertion e.g. getting out of a chair	Severe breathing difficulty; can't complete sentences at rest	Highest of 2a or 2b
2b	or Respiratory rate (per minute)	12-20	21-24	9-11 or<br>25-29	8 or less, or<br>30 or more	

Score any breathlessness that patient or carer is concerned about. Take account of pre-existing conditions such as COPD.
Assess respiratory rate by video, ask patient to place hand on chest. An anxious patient may be hyperventilating.

		Score 0	Score 1	Score 2	Score 3 => refer urgently	Score
3	Trajectory of breathlessness	Same or better than yesterday	Breathless, worse than yesterday	-	Significant deterioration in last hour	

Pay careful attention to a history of recent worsening of breathlessness, especially if this is what concerns patient or carer.

		Score 0	Score 1	Score 2	Score 3 => refer urgently	Score
4a	Oxygen saturation at rest	96%<br>or above	95% (don't do 40-step test unsupervised)	94% (don't do 40-step test unsupervised)	93% or below (don't do 40-step test)	Highest of 4a, 4b & 4c
4b	or Saturation after 40 steps	Fall of 0-1%	-	Fall of 2%	Fall of 3% or more	
4c	or Profound tiredness or fatigue	None<br>or mild	Noticeably more tired doing usual activities	Struggling to get out of bed	Unable to speak because of tiredness	

Patient should have warm hands and place oximeter device correctly. Lower thresholds if patient has chronic lung disease with known hypoxia (typically by 6%, but will vary – use usual baseline readings to adjust if known). Do exertion test only if clinician in attendance or if saturation is 96% or higher at rest (lower threshold for chronic lung disease). Saturation levels may fall for 1 min after stopping exercise. Most patients

with covid-19 feel some fatigue, but profound fatigue may be a feature of 'silent hypoxia'. Take account of patient's baseline level of fatigue.

5a	Temperature	≤ 38 °C	38.1-39 °C	> 39 °C or < 35 °C	-	Highest of 5a or 5b
5b	or Feeling feverish with shivers	None	Feverish or chills	Uncontrollable shivering	-	

A tympanic thermometer is preferred. Use highest recorded level in last 24 hours. A low reading may reflect user error.
If the patient has no reliable thermometer (or in addition to the temperature reading), explore for a description consistent with rigors.

6	Time from first symptom (days)	7 or fewer	8 or more	-		

Number of days since onset of first symptom

7	Muscle aches	None or mild	Moderate	Severe		

8	Cognitive decline	No	Less mentally alert than usual	New and worsening confusion	Reduced level of consciousness	

9	On COVID-19 shielded list (or has been inadvertently left off it)?	No	Yes	-	-	

Includes: • organ transplant • current chemotherapy or immunotherapy • severe lung condition such as cystic fibrosis • sickle cell anaemia • high dose steroids or other immunosuppressants • blood or bone marrow cancer • lung cancer on radiotherapy • splenectomy

10	Other risk factors for poor outcome in COVID-19?	0-2	3 or more	-	-	

For example: *• Age > 65 • BMI > 35 • male • non-White ethnicity • diabetes • hypertension • coronary heart disease • chronic kidney disease • vulnerable and isolated*

TOTAL	

Clinical concern component (be guided by clinical concern whatever the RECAP score; do not add this into the score but use it to over-ride the score if appropriate):

After assessing the patient, what is your level of clinical concern (regardless of RECAP score)?	Low	Moderate	High	Extremely high

RECAP score	Meaning	Recommended action
7 or more total or 3 on any item or extremely high level of clinical concern	HIGH RISK	Consider urgent referral
4-6 or more total or high level of clinical concern	MODERATE RISK	See in hot hub or virtual ward, or arrange home visit
0-3 total	LOW RISK	Advice and monitor at home

For peer review only

BMJ Open

Remote COVID-19 Assessment in Primary Care (RECAP) early warning score: item development

Journal:	*BMJ Open*
Manuscript ID	bmjopen-2020-042626.R1
Article Type:	Original research
Date Submitted by the Author:	05-Oct-2020

Complete List of Authors:	Greenhalgh, Trisha; University of Oxford, Nuffield Department of Primary Care Health Sciences<br>Thompson, Paul; University of Oxford, Department of Experimental Psychology<br>Weiringa, Sietse; University of Oxford, Nuffield Department of Primary Care Health Sciences<br>Neves, Ana Luisa; Imperial College London,<br>Husain, Laiba; University of Oxford, Nuffield Department of Primary Care Health Sciences<br>Dunlop, Merlin; Ardens Ltd<br>Rushforth, Alexander; University of Oxford, Nuffield Department of Primary Care Health Sciences<br>Nunan, David; University of Oxford, Nuffield Department of Primary Care Health Sciences<br>de Lusignan, Simon; Oxford University, Nuffield Department of Primary Care Health Sciences<br>Delaney, Brendan; Imperial College London, Surgery and Cancer

**Primary Subject Heading**:	Health services research
Secondary Subject Heading:	Infectious diseases, General practice / Family practice, Health informatics
Keywords:	INFECTIOUS DISEASES, Health informatics < BIOTECHNOLOGY & BIOINFORMATICS, VIROLOGY, COVID-19

SCHOLARONE™
Manuscripts

I, the Submitting Author has the right to grant and does grant on behalf of all authors of the Work (as defined in the below author licence), an exclusive licence and/or a non-exclusive licence for contributions from authors who are: i) UK Crown employees; ii) where BMJ has agreed a CC-BY licence shall apply, and/or iii) in accordance with the terms applicable for US Federal Government officers or employees acting as part of their official duties; on a worldwide, perpetual, irrevocable, royalty-free basis to BMJ Publishing Group Ltd ("BMJ") its licensees and where the relevant Journal is co-owned by BMJ to the co-owners of the Journal, to publish the Work in this journal and any other BMJ products and to exploit all rights, as set out in our licence.

The Submitting Author accepts and understands that any supply made under these terms is made by BMJ to the Submitting Author unless you are acting as an employee on behalf of your employer or a postgraduate student of an affiliated institution which is paying any applicable article publishing charge ("APC") for Open Access articles. Where the Submitting Author wishes to make the Work available on an Open Access basis (and intends to pay the relevant APC), the terms of reuse of such Open Access shall be governed by a Creative Commons licence – details of these licences and which Creative Commons licence will apply to this Work are set out in our licence referred to above.

Other than as permitted in any relevant BMJ Author's Self Archiving Policies, I confirm this Work has not been accepted for publication elsewhere, is not being considered for publication elsewhere and does not duplicate material already published. I confirm all authors consent to publication of this Work and authorise the granting of this licence.

For peer review only

Original research paper resubmitted to BMJ Open October 2020

Remote Covid-19 Assessment in Primary Care (RECAP) early warning score: item development

Trisha Greenhalgh (Professor)[1]

Paul Thompson (Researcher) [2]

Sietse Weiringa (Researcher)[1]

Ana Luisa Neves (Researcher)[3]

Laiba Husain (Researcher)[1]

Merlin Dunlop (GP)[4]

Alex Rushforth (Researcher)[1]

David Nunan (Researcher)[1]

Simon de Lusignan (Professor)[1]

Brendan Delaney (Professor)[3]

For peer review only - http://bmjopen.bmj.com/site/about/guidelines.xhtml

[1] Nuffield Department of Primary Care Health Sciences, University of Oxford, Oxford OX2 6GG, UK

[2] Department of Experimental Psychology, University of Oxford, Oxford OX2 6GG, UK

[3] Institute of Global Health Innovation, Imperial College London, London

[4] Ardens-Q Ltd

Corresponding author: Professor Trisha Greenhalgh, Nuffield Department of Primary Care Health Sciences, University of Oxford, Oxford OX2 6GG, UK - phone: +44 (0)1865 289294, email: trish.greenhalgh@phc.ox.ac.uk.

Keywords: Early warning score, RECAP (Remote Covid Assessment in Primary Care) score, Covid-19, Medical record systems, computerized.

Word count: 3956

For peer review only

ABSTRACT

Background

To develop items for an early warning score (RECAP: REmote Covid-19 Assessment in Primary Care) for patients with suspected Covid-19 who need escalation to next level of care.

Methods

The study was based in UK primary health care. The mixed-methods design included rapid review, Delphi panel, interviews, focus groups and software development. Participants were 112 primary care clinicians and 50 patients recovered from Covid-19, recruited through social media, patient groups and snowballing. Using rapid literature review, we identified signs and symptoms which are commoner in severe Covid-19. Building a preliminary score from these, we ran four rounds of an online Delphi panel with 72 clinicians, the last incorporating fictional vignettes, collating data on R software. We refined the score iteratively in response to quantitative and qualitative feedback. Items in the penultimate score were checked against narrative interviews with 50 Covid-19 patients. We required, for each item, at least 80% clinician agreement on relevance, wording and cut-off values, and that the item addressed issues and concerns raised by patients. In focus groups, 40 clinicians suggested further refinements and discussed workability of the instrument in relation to local resources and care pathways. This informed design of an electronic template for primary care systems.

Results

The pre-validation RECAP-V0 score comprises a red flag alert box and 10 assessment items: pulse, shortness of breath or respiratory rate, trajectory of breathlessness, pulse oximeter reading (with brief exercise test if appropriate) or symptoms suggestive of hypoxia, temperature or fever symptoms, duration of symptoms, muscle aches, new confusion,

For peer review only - http://bmjopen.bmj.com/site/about/guidelines.xhtml

shielded list, and known risk factors for poor outcome. It is not yet known how sensitive or specific it is.

Conclusions

Items on this preliminary score align strongly with published evidence, clinical judgement and patient experience. The validation phase of this study is ongoing.

Study registration

Clinicaltrials.gov (NCT04435041).

Article summary

Strengths and limitations of the study

- First systematic study to develop a Covid-19 early warning score for primary care
- Captures clinician and patient experience of the deteriorating patient with Covid-19
- Combines extensive qualitative research and quantitative consensus methodology
- Conducted at speed in the midst of first wave of Covid-19
- Items have strong face validity but formal validation is still ongoing
- RECAP is a severity prediction score, not a diagnostic score

For peer review only

Introduction

Some patients with Covid-19 experience deterioration (usually at around day 8).[1 2] There is therefore a need for research to develop accurate early warning scores – clinical prediction models designed to identify patients who need escalation to next level of care.[3] Such scores need to be both highly specific (detecting all patients who need onward referral) and fairly sensitive (excluding all or most patients who do not). A recent systematic review of prediction models for Covid-19 concluded that "proposed models are poorly reported, at high risk of bias, and their reported performance is probably optimistic" (page 2).[4] That review identified no evidence-based prediction models for primary care settings, nor did a citation-track of the article (which identified over 300 subsequent papers).

Assessment of a patient with suspected acute Covid-19 in primary care is fraught with uncertainty, since its clinical course differs from other pneumonias[5] and because most patients will be assessed remotely (i.e. by phone or video).[6] The UK Royal College of General Practitioners has cautiously endorsed the use of NEWS2 (National Early Warning Score 2) alongside clinical judgement for the assessment of patients with suspected acute Covid-19.[7] NEWS2 is calculated from the patient's temperature, pulse rate, respiratory rate, systolic blood pressure, pulse oximetry reading and new confusion.[8] The National Institute for Health and Care Excellence (NICE) rapid guideline on management of Covid-19 pneumonia in the community makes the guarded statement that the NEWS2 score "may be useful" in assessing deterioration but that the patient should not be brought in for a face-to-face assessment solely to calculate a NEWS2 score (paragraph 3.7).[9] But NEWS2 was developed for a different purpose (see Discussion), and requires data that may be difficult to obtain. A recent preprint suggest that it correlates poorly with severity of Covid-19.[10]

We sought to develop and validate a primary care early warning score for Covid-19 based on data that can be reliably collected during a remote consultation. This paper describes the development of a version 0 of the score which can be formally validated. It does not cover the actual validation of the score.

For peer review only - http://bmjopen.bmj.com/site/about/guidelines.xhtml

Method

Study design and governance

The study was part of the Remote By Default research programme, funded by UK Research and Innovation Covid-19 Emergency Research Fund. It consisted of five phases (Figure 1): rapid review, a four-round Delphi panel of primary care clinicians, semi-structured interviews and focus groups with patients, focus groups with primary care clinicians, and electronic template development. This in-depth qualitative design was chosen because of the novelty of the condition, the high degree of clinical uncertainty surrounding its acute management, and the added complexity of the need for remote assessment (which required judgements to be made without having fully examined the patient). For all these reasons, a detailed qualitative phase was considered essential before progressing to a standard validation exercise. The study was overseen by an independent advisory group with a lay chair and a separate patient advisory group. Ethics approvals are included at the end of the paper.

FIGURE 1

Rapid review

Detailed methods have been published elsewhere [5]. Briefly, fortnightly keyword searches were conducted from March to June 2020 of PubMed for English-language systematic reviews and LitCovid and MedRxiv for reprints. Methodological quality of reviews was assessed using AMSTAR-2.[11] Primary studies within included reviews were cross-checked for duplication before extracting data on each symptom or sign in both mild and severe Covid-19. The synthesis was continually updated over the course of the study period from the most up-to-date, highest-quality systematic reviews. To construct the draft RECAP score, we selected symptoms and signs that were significantly commoner in severe than mild disease.

For peer review only - http://bmjopen.bmj.com/site/about/guidelines.xhtml

Delphi panel

Delphi is a structured approach to working towards consensus.[12] Steps include defining a problem, selecting a panel of experts (both academics and clinicians), supplying evidence along with key uncertainties, collecting quantitative (numerical scores) and qualitative (free text) data on a set of statements, feeding scores and comments back to panel members, and repeating until residual disagreement cannot be resolved. Advantages of this method include practicality (it can be done online, asynchronously, without specialist tools), anonymity (participants know the average group score but not individuals' scores) and iteration (feedback prompts outliers to either defend their score to the group or change it).[13 14]

Following established methodology,[12] we used a social media announcement (TG's Twitter account) and snowballing from people thus recruited to obtain 72 volunteers (68 general practitioners, three nurse practitioners and one paramedic), all of whom were actively involved in assessing acutely ill patients with suspected Covid-19. Ten of these were clinical academics. The 'draft 0.1' RECAP score developed from rapid review was entered onto a questionnaire using Survey Monkey (homeworking during lockdown prevented us from accessing our usual professional survey software).

We sent brief instructions to participants, including a summary of what was known about prognostic factors in Covid-19. We gave them five days to complete the survey, scoring each item for relevance (should it be included in the score?) and wording (how would they improve it?), and add free text comments. We sent two prompts to non-responders. A researcher (PT) imported the raw data into R and collated quantitative responses using simple descriptive statistics (percentage selecting, for example, yes as currently worded, yes but change wording, not sure, probably not and definitely not), and tabulated qualitative responses, which TG subsequently arranged under broad clinical categories and themes. We amended the draft RECAP score before circulating a summary of data, notes on changes and reasons for them, and the next iteration of the score. This was repeated for a second and third round.

For peer review only - http://bmjopen.bmj.com/site/about/guidelines.xhtml

The fourth round included only three residual items on which we had not yet achieved our goal of 80% consensus, plus a set of five fictional vignettes which incorporated various scenarios (such as missing data, untrustworthy technology, complex social circumstances, and comorbidities) that participants had proposed in their free text responses. They were asked to calculate a RECAP score for each of these five vignettes and indicate their level of clinical concern. This fourth round was repeated following a report from several participants that they had been unsure how to calculate the RECAP score (so had guessed); in the repeat round, we adjusted the software to calculate the score automatically.

Patient interviews

UK-based survivors of Covid-19 were recruited using three methods: a social media call (again, TG's Twitter account), snowballing from those who responded (to access those not on social media), and an email sent from a support group for people with prolonged Covid-19 symptoms (LongCovidSOS).

AR conducted an initial round of 20 audio recorded interviews by telephone or MS Teams, taking verbal consent. Patients were asked to narrate their various contacts with healthcare services, including how symptoms were assessed by call handlers or clinicians. Relevant sections of interviews were transcribed and read independently by AR and TG. Early in the study, data were extracted into a word document and arranged into categories to correspond to the items on the RECAP score, and any additional categories not included in the score. Later in the study, these data were combined with comments from 30 additional survivors of acute Covid-19 extracted from a larger dataset of interviews and focus groups. The data were formally coded in NVIVO software and checked against the final RECAP-0 score to confirm that all relevant issues raised by patients had been captured.

Clinician focus groups

Focus groups are a qualitative method designed to capture both individual perspectives and group dynamics (such as empathy, humour and conflict).[15 16] There is an established methodology for conducting them by video-conference.[17] A new sample of 40 primary care clinicians (28 general practitioners, 11 nurse practitioners, 1 paramedic) who regularly

For peer review only - http://bmjopen.bmj.com/site/about/guidelines.xhtml

assess suspected Covid-19 patients was recruited by a social media invitation enhanced by snowballing. Focus groups were held by Zoom and lasted 90 minutes. Prior to the group, we circulated ground rules (e.g. about confidentiality) and invited participants to read and score the five vignettes to familiarise themselves with RECAP-V0. Each focus group was facilitated by two academic general practitioners (TG, ALN or SW) guided by a list of prompt questions (reproduced in Additional File 1 on bmj.com). Briefly, we sought their views on the RECAP-V0 score (including a mock-up of the electronic template), and how they felt it would align with local clinical presentations, care pathways, electronic templates, resources and workflows. We asked what challenges they anticipated with its implementation.

Focus groups were video-recorded with consent. They were not fully transcribed but the clinical researchers all listened independently and transcribed selected sections. This free text was organised thematically on an Excel spreadsheet using the Framework method.[18] Insights from focus groups were fed iteratively into the software design phase described below.

Electronic template design and development

MD and BD matched individual RECAP-0 items with existing SNOMED-CT codes where possible. A template was constructed using drop-down menus, and inserted as a supplement to the Ardens Covid-19 assessment template built for EMIS Web (Egton Medical Information Systems). A further round of review of these codes was conducted with input from NW London CCGs (S-J Knight) and Dr Simon Gordon.

Results

Rapid review

Five factors appeared to predict poor outcome in Covid-19: persistent fever, shortness of breath, low oxygen saturation, muscle aches, and certain comorbidities.[5] Patients with severe Covid-19 may develop shock (manifest, for example, as deteriorating conscious level,

For peer review only - http://bmjopen.bmj.com/site/about/guidelines.xhtml

hypotension, and reduced urine output). Based on these findings, we constructed a preliminary 13-item score. Later in the study, additional questions emerged which prompted new rapid reviews: assessment of breathlessness,[19 20] how to measure exertional desaturation accurately and safely in patients with suspected Covid-19,[21] and the reliability of smartphone oximeters[22] and smartphone blood pressure measuring apps.[23]

Delphi panel

The Delphi panel was conducted in April and May 2020. Round 1 had a sample of 69 clinicians; three more joined for rounds 2 to 4. Of these, 62, 53, 56 and 51 completed the surveys respectively – response rates of 90%, 74%, 78% and 71%. Progression towards quantitative consensus across the four rounds is shown in Table 1.

TABLE 1

In sum, at least 80% agreement on relevance, wording and cut-off values for particular scores was eventually achieved for eight variables: pulse, temperature, symptoms of fever (for use particularly if the patient does not have a reliable thermometer), oxygen saturation level, tiredness (which, if severe, may indicate hypoxia and hence potentially substitute for an oximeter reading), muscle aches, and known risk factors (comorbidities). Additional items with moderate agreement included demographic risk factors (77% agreement), oxygen saturation level after a 40-step exercise test (75%), trajectory of breathlessness (73%) and duration of temperature (63%).

Our qualitative dataset from the Delphi panel included over 200 pages of comments. Key themes are summarised and illustrated in Additional File 2 on bmj.com. These, along with rapid review, allowed us to characterise the clinical features of the deteriorating Covid-19 patient in primary care (Box 1)

BOX 1

In addition to prompting new rapid reviews, the Delphi qualitative data shaped the development of the score in several ways. Particular forms of words (for example, to

For peer review only - http://bmjopen.bmj.com/site/about/guidelines.xhtml

question patients about the severity and rate of deterioration of breathlessness) enabled us to refine our items and cut-off levels. Participants alerted us to existing guidance and protocols used either nationally or locally (there was strong consensus that any new score should complement rather than replace these). Comments about missing or untrustworthy data when a patient was being assessed at home via telephone or video link prompted us to develop default scores or alternative questions to compensate for such deficiencies. The free text comments included rich data, based on real clinical experiences, from which we were able to construct the vignettes used in discussions. Numerous comments on the practicalities of applying the score prompted us to set up focus groups to explore these operational challenges further.

The five fictional vignettes and the results of the scoring exercise on these are reproduced in Additional File 3 on bmj.com. In sum, whilst there was considerable variation in the actual score given (for example, in whether clinicians judged an ill-defined set of symptoms as “moderately” or “severely” tired), in all five vignettes the RECAP score as calculated appeared to prompt an appropriate and cautious response. For example, in a case of an elderly South Asian patient who spoke no English and with no equipment at home for the family to take measurements, all but one participant were prompted by the RECAP score to assess the patient in a face-to-face encounter. In a case of an African Caribbean man with profound tiredness and rapidly worsening breathlessness in the second week of his illness, all participants were prompted to arrange urgent transfer to hospital. Free text comments in several vignettes indicated that some respondents’ level of clinical concern had not been especially high and that they were surprised that the RECAP score was so high.

Patient interviews

Of the 20 patient interviews in our original sample, 15 included detailed descriptions of deteriorating symptoms in the acute phase of Covid-19. These included worsening breathlessness, inability to speak in sentences, profound fatigue without feeling short of breath, high temperature, and symptoms interpreted as anxiety but which may have been acute tachycardia. Patients also described being reassured by remote assessments using video examination and home monitoring equipment.

For peer review only - http://bmjopen.bmj.com/site/about/guidelines.xhtml

"… when I was speaking to them they were listening to me breathing and watching my chest and things. And they were seeing my breathing through the video link, wanting to see a clear vision of my chest area and counting my respiratory rate. And looking back when I spoke to them I was breathless. […] I've already got a pulse oximeter and a blood pressure machine. […] The pulse ox[imeter] was really good. What I had to do is when they video called I had to put it on my finger and wave it at the screen and they were able to monitor it." [patient NM1]

Some patients described long waits to get through to NHS 111 (the English telephone triage service), being asked "tick box" questions by call handlers or clinicians, and feeling dismissed on the basis of such questions even when they were concerned about the extent and pace of their own (or a relative's) deterioration. For example:

"You can't make a diagnosis on the phone because you don't get to see the physical symptoms. They [clinicians] never saw the [patient's] rash, I described it but they didn't actually get to see it. They couldn't see she was sweating all around her hairline and her face was super pale. The overall picture they couldn't see – they just had written down some numbers." [relative of patient, HG1]

The "exercise" being referred to in the quote below is probably the Roth score,[24] which is likely neither sensitive nor specific in the assessment of Covid-19 patients.[20]

"There was an exercise where I need to count to something a certain number of times. They did a small exercise. But now they know you can have severe hypoxia without clear shortness of breath when talking. That was the assessment. They said 'I know it's rough, soldier through. You're 34 years old. There's no point in escalating this.'" [patient SN1]

These patient data helped us refine the wording of the questions on the RECAP score and also the clinical description in Box 1. In particular, the patient data emphasised the importance of developing the score as an adjunct to expert clinical judgement and not as a

For peer review only - http://bmjopen.bmj.com/site/about/guidelines.xhtml

substitute for it. Additional qualitative data from 30 further Covid-19 survivors confirmed these themes and identified one additional relevant finding: that mismatches between the clinician's and patient's assessment of severity occurred, and were sometimes attributed by the clinician to anxiety:

> *"He [call handler who was being advised by nurse] said you seem to be able to talk in complete sentences. I was concerned because I thought I couldn't. I said I'm trying my best but I am struggling to talk, you might not hear it but it's definitely been happening this evening. […]. And the nurse asked the call handler to ask me if I suffer from anxiety."*

Focus groups

In focus groups, clinicians described Covid-19 as a disease with "surprises". They were especially concerned with patients whose initial course was unremarkable but who subsequently deteriorated rapidly. They wanted an early warning score not primarily to identify "red flag" patients (whose need for urgent hospital referral they felt was usually obvious), but for assessing moderately sick patients and tracking their progress over time. They considered the RECAP score to have high face validity and to reflect their level of clinical concern on the vignettes. Some felt that RECAP would be less useful for assessing an unwell patient who did not have Covid-19. All three groups proposed the same additional item on the score (patient less alert than usual or new confusion), and there was also agreement on a minor change to one item (changing "duration of temperature" to "duration of symptoms"). Participants made suggestions for layout and ordering of items on the electronic template, emphasising simplicity, ease of use and "human factors".

The RECAP score

Data from the rapid reviews, Delphi panel, patient interviews and clinician focus groups contributed to development and refinement of the v0 version of the RECAP score (Figure 2) which will go forward for further development and validation. RECAP-v0 consists of 10 items, three of which include alternative options designed to enable assessment even in the

For peer review only - http://bmjopen.bmj.com/site/about/guidelines.xhtml

absence of technologies (video camera, thermometer, oximeter). Further explanatory text is given in Additional File 4 on bmj.com.

FIGURE 2

Electronic template design and development

To make an electronic version of the RECAP score, we selected pre-coordinated terms that were uniformly present in EMIS and SystmOne EHR systems, so that the same terms could be used and exported for linkage nationwide. The template included a prompt to capture the patient's verbal consent (supported by a url for the information sheet) for data linkage (as the relevant SNOMED code bound to our unique NHS Clinical Research Network Portfolio Number), as well as data previous entered and saved as part of the standard Covid-19 assessment. Layout and ordering of items was adapted in response to focus group comments and insights. It proved impossible to identify suitable codes for severity of myalgia, so the final template only supports presence/absence of myalgia.

Discussion

Statement of principal findings

This mixed-method study has produced five key findings. First, we have developed, through consensus, a rich description of clinical deterioration in the primary care patient with Covid-19 (Box 1). Second, we have achieved very high agreement among 72 front-line primary care clinicians and academics for the inclusion of a particular wording on the following variables for inclusion in an early warning score: pulse rate (88% agreement), temperature (89%), fever symptoms (91%), respiratory rate (84%), shortness of breath (79%), pulse oximeter reading (80%), post-exercise fall in pulse oximeter reading (75%), tiredness (88%), muscle aches (82%), shielded list (86%), and other risk factors for poor outcome (77%), along with affirmation of these by a second sample of 40 clinicians, who added duration of symptoms and new confusion. Third, we have obtained a high level of agreement on the numerical or

For peer review only - http://bmjopen.bmj.com/site/about/guidelines.xhtml

descriptive cut-off levels for allocating points to different signs and symptoms (details in Table 1). Fourth, we have confirmed that the items on the score reflect the concerns of patients. Finally, we have surfaced, and begun to address, some of the human factors and operational challenges of implementing the RECAP-V0 score in different primary care services and settings across the UK.

Strengths and limitations of the study

This is the first study systematically to capture the clinical experience and wisdom of front-line primary care practitioners, as well as the experience of patients, in relation to the assessment of the deteriorating Covid-19 patient who has not yet been referred to hospital. Early Chinese studies on Covid-19 included only hospital patients.[1 25] Other community-based studies identified symptoms (notably loss of smell) associated with Covid-19 but which lack prognostic significance.[26]

Our study was undertaken at speed, in the midst of the first wave of the pandemic, and was influenced by the practicalities of the UK National Health Service under unprecedented stress. Despite these pressures, we believe we have achieved a sufficiently large and diverse sample of front-line practitioners and patients to be confident that the findings reflect current best practice and patient priorities.

The main limitation is that we have not yet validated the score (see 'Next steps' below). Another limitation is that the score relies partly on UK-specific data (specifically the 'shielded' category). This aspect of the score is, however, readily adaptable to categories used in other countries. Because the score is preliminary, we do not yet have firm evidence that the weightings assigned to different items and cut-off levels are appropriate.

Comparison with previous literature

To date, most early warning scores have been developed for use in hospital inpatients using routinely collected vital sign data.[27] In UK hospitals, the NEWS2 score has become a common language of sickness with positive implications for patient safety (especially in

For peer review only - http://bmjopen.bmj.com/site/about/guidelines.xhtml

relation to sepsis).[28] NEWS2 is recommended by NICE guidelines both in general[29] and as a component of the critical care of Covid-19 patients,[30] though it has been heavily criticised even in hospital settings.[27 31-34] NEWS2 has been used in pre-hospital settings by ambulance crews[31] and in early detection of suspected sepsis in primary care[35]. However, it has not been formally validated in general practice,[36] so its sensitivity and specificity in that context are unknown. Its positive predictive value is low even in hospital and ambulance settings, and is likely to be even worse in primary care because serious illness is rare.[37] A rise in NEWS2 appears to be a relatively late indicator of deterioration, typically triggering only in the last 12 hours before transfer to critical care.[27] For all these reasons, NEWS2 might cause harm from both under- and over-referral in a primary care setting, though there is preliminary evidence that it may have some value for Covid-19 in care home residents, who tend to be sicker.[38]

The key differences between NEWS2 and RECAP-0 are as follows. Whereas NEWS2 was designed to be calculated from observations taken in hospital by trained staff and is based solely on signs, RECAP is designed to be completed in primary care as part of a clinician-patient conversation along with a (limited) remote physical examination, and is based on both symptoms and signs. With the exception of pulse rate (which defaults to a value of 1 if no reading is possible), all items requiring a physical examination include an alternative item based solely on symptoms. This will allow the clinician to populate the score even when the assessment is being done remotely and the patient is unable or unwilling to use equipment, thereby reducing the danger of missing data.[27]

Whilst we developed the RECAP score as a disease-specific instrument, a reviewer of a previous draft of this paper suggested that it score may prove useful in both Covid-19 and non-Covid acute deterioration since (he hypothesised) most symptoms of acute deterioration are not disease-specific. He drew our attention to a new study from Uganda which identified 12 "high-risk chief complaints" in a pre-hospital population which were associated with increased acute mortality in the subsequent days.[39] Interestingly, one of these was "difficulty speaking" which had not previously been included as a red flag symptom or prognostic marker but which was prominent in our qualitative data.

For peer review only - http://bmjopen.bmj.com/site/about/guidelines.xhtml

A recent publication describes the development and validation of an in-hospital severity score for suspected Covid-19.[40] The International Severe Acute Respiratory and emerging Infections Consortium Coronavirus Clinical Characterisation Consortium (ISARIC-4C) study included over 35,000 patients in the derivation dataset and over 22,000 in the validation dataset. The final 4C Mortality Score included eight variables, six of which (respiratory rate, conscious level, peripheral oxygen saturation, comorbidities, gender and age) overlap with RECAP. The other two (urea and C-reactive protein) require a blood test. Unlike the 4C Mortality Score, RECAP includes several items based on (or substitutable with) the patient's *subjective* symptoms including shortness of breath and muscle aches, both of which have been shown to correlate strongly with disease severity.[5] RECAP also includes heart rate, as well as items based on time course (persisting fever on day 8 and trajectory of breathlessness), which reflect the clinical judgement of our Delphi panel of clinicians. The validation phase of the study will test whether any or all of these additional items improve the predictive value of the RECAP score.

Next steps

The transparent reporting of a multivariable prediction model for individual prognosis or diagnosis (TRIPOD) statement[41] states that development of a prognostic model (of which an early warning score is one example) requires two phases: instrument development and instrument validation. The study reported here has ensured that the first component of instrument development has captured three important dimensions: the existing evidence base from the research literature; the experience and intuition of front-line primary care clinicians; and the experience of patients. The second component of instrument development is to gather data on these important dimensions of clinical observation and carry out analysis.

In the next phase of this study (ongoing), we are completing development and validating the RECAP score using data linkage between general practice electronic records and national datasets (local data from North West London's iCare and nationally via the RCGP Research and Surveillance Centre) using the primary outcome of hospital admission and secondary

For peer review only - http://bmjopen.bmj.com/site/about/guidelines.xhtml

outcomes of intensive care unit admission and death. Further details are obtainable from the RECAP study website.[42]

Because of the novelty of the disease and the urgency of the research question, we decided to place our interim findings in the public domain so as to allow other teams to test and improve the RECAP score in parallel with our own continuing research (rather than, as is more commonly the case, seeking to ensure that our own validation study is published first). We welcome offers of collaboration from established research teams.

Until the findings of the validation study are published, the validity of this instrument is unknown. Even if RECAP proves sensitive and specific for identifying the need for urgent escalation of care, it should be noted that this instrument is a *severity prediction tool*, not a diagnostic tool. It does not include items which are highly specific for diagnosing Covid-19 but are not predictive of its severity (e.g. loss of smell), and it includes many items (such as standard red flag indicators) which are not specific for Covid-19.

For peer review only

For peer review only - http://bmjopen.bmj.com/site/about/guidelines.xhtml

Figure 1: Study flowchart

See separate file

Figure 2: Summary of RECAP-0 score

See separate file

For peer review only

For peer review only - http://bmjopen.bmj.com/site/about/guidelines.xhtml

Table 1: Results of Delphi panel

Focus of item	Percentage agreeing with item as worded			
	Round 1 (n = 62)	Round 2 (n = 53)	Round 3 (n = 56)	Round 4 (n = 51)
Pulse rate (wording of item)	73%	70%	**88%**	
Cut-off values for each category	71%	77%	**84%**	
Temperature (wording)	58%	75%	**89%**	
Cut-off values	55%	81%	**89%**	
Duration of temperature* (wording)	58%			**63%**
Cut-off values				**76%**
Symptoms of fever (e.g. chills, shivers)	29%	75%	**91%**	
Cut-off values	63%	87%	**95%**	
Respiratory rate (wording)	61%	85%	**84%**	
Cut-off values	76%	85%	**91%**	
Shortness of breath (wording)	39%	75%	**79%**	
Cut-off values	44%	79%	**93%**	
Trajectory of breathlessness				**73%**
Cut-off values				**67%**
Oxygen saturation level	65%	79%	**80%**	
Cut-off values	65%	85%	**82%**	
Oxygen saturation level after exercise	-	68%	**75%**	
Cut-off values		85%	**91%**	
Clinical suspicion of hypoxia	47%			
Cut-off values	55%			
Tiredness (wording)	42%	83%	**88%**	
Cut-off values	73%	89%	**84%**	
Muscle pains or aches	66%	-	64%	**82%**
Cut-off values	47%	-	**79%**	
Risk factors (comorbidities)	53%	57%	**86%**	
Cut-off values			**82%**	
Risk factors (demographic e.g. age, ethnicity)			**77%** **86%**	
Indicators of shock (including conscious level, new confusion**, low or no urine output, cold and clammy, mottled skin)	61%	64%	Moved to 'red flag' box to align with national guidance	-
Other red flag symptoms e.g. central chest pain	73%	34%		
Clinical concern	74%	77%	**86%**	

* 'Duration of temperature' was changed to 'duration of symptoms' after focus group discussion, with 8 days seen as the cut-off for clinical concern

** New confusion was subsequently proposed as a separate RECAP item by focus group participants, who did not agree that it should be an automatic 'red flag' sign

For peer review only

Box 1: The clinical course of the deteriorating Covid-19 patient in primary care

Synthesised from our qualitative data, supplemented from published sources

Covid-19 may present in primary care as a viral upper respiratory tract infection (e.g. sore throat), lower respiratory tract infection (e.g. cough, fever and mild dyspnoea), influenza-like illness (with fever, chills, headache and myalgia), or gastro-intestinal illness (with abdominal pains, nausea and diarrhoea).[2 6] Most patients have a relatively mild, self-limiting illness, but an unknown proportion (perhaps 10%) deteriorate, usually in week 2. Certain symptoms common in week 1, such as cough, mild fatigue and anosmia, do not appear to have prognostic significance.[5 43]

It is important to date-stamp the onset of first symptoms.[6] Severe dyspnoea, especially at rest, may indicate progression of lung involvement. The trajectory of dyspnoea is important, as acute respiratory distress syndrome occasionally follows quickly from the onset of breathlessness.[44] Formal scores for assessing dyspnoea severity appear to have a significant false negative rate and should not be used.[20] A careful history, noting what the patient is able to do and what they cannot do today that they could do yesterday, is likely to be more important.[20] A patient's or carer's concern about the severity of breathlessness may be significant and should not be dismissed as "anxiety".

Pulse oximeter readings are extremely useful in assessing unwell patients with Covid-19, so long as the device is reliable (smartphone apps are inherently inaccurate and should not be used[22]) and the patient or a relative is capable and confident to use it. The finger must be warm. Whilst a low oximeter reading is concerning, a normal one should not necessarily reassure, as young fit patients in particular can compensate well in the early stages of deterioration.

So-called silent hypoxia, defined as the development of respiratory failure without the subjective perception of dyspnoea, is a recently-described feature of severe Covid-19 and appears to have a poor prognosis.[43 45-48] Anecdotal accounts suggest that in some patients, silent hypoxia may manifest as profound tiredness, but we could not find published research on this association. New confusion (especially in older patients with comorbidity) was considered by clinicians in our sample to be a poor prognostic sign in Covid-19, but at the time of writing, evidence for this is limited.[49]

Covid-19 lung damage tends to be manifest as a perfusion defect (i.e. difficulty transferring oxygen across the alveolar membrane) rather than a ventilatory defect (difficulty getting air to the alveoli, as in asthma).[44] This may explain why Covid-19 can behave similarly to pneumocystis pneumonia in producing a fall in pulse oximetry reading on exertion (or in the minutes following exertion).[21 50] Because of this, patients with suspected Covid-19 should not be subject to exercise testing unless there is a clinician present if their resting pulse oximetry reading is abnormal (below 96%).

An unwell patient may or may not have Covid-19. An overall assessment is needed using questions relating to (for example) hydration status, dizziness, falls, central chest pain, fall in blood pressure (if the patient has equipment at home), change in mental status (including lethargy, new confusion, difficulty in rousing), central cyanosis (e.g. blue lips), and severe reduction in urine output. For this reason, a standard 'red flag' checklist should be quickly reviewed in all unwell patients.

There are some well-established risk factors for developing Covid-19 and worse outcome (e.g. age, non-white ethnicity, high body mass index and comorbidities including cardiovascular disease, hypertension and active cancer).[51-53] The extent to which these risk factors should be applied to 'load the score' of a patient who appears to have a mild form of the disease is not yet known, especially since shielded patients are the ones for whom a hospital or clinic visit carries most risk.

For peer review only - http://bmjopen.bmj.com/site/about/guidelines.xhtml

Declarations

Ethics approvals and consent to participate

Ethical approval for the patient interviews was obtained from East Midlands – Leicester Central Research Ethics Committee (IRAS Project ID: 283196; REC ref 20/EM0128) on 4th May 2020. Ethical approval for the validation study (including holding the focus groups and building and installing the software on primary care record systems) was obtained from Greater Manchester Research Ethics Committee on 20th May 2020 (IRAS Project ID: 283024; REC ref 20/NW/0266) and subsequent amendments. In accordance with our ethics approvals, all patient participants gave written informed consent and all clinician focus group participants gave verbal consent which was recorded on the video. We sought advice from the chair of our local NHS Research Ethics Committee who considered that the online Delphi panel did not require formal ethical approval; consent from these participants was obtained by email.

Contributorship statement

TG conceptualised the study and oversaw all work packages described in the paper. She led or contributed to the various rapid reviews, recruited participants, led data analysis and summarising, and wrote the paper. PT designed and led the operational and quantitative aspects of the Delphi study and the quantitative anlyses. SW and ALN recruited participants, co-facilitated the focus groups and participated in data analysis and writing up. LH provided assistance with all aspects of the research. MD designed the templates for putting RECAP items on general practice electronic records and participated in focus groups. AR interviewed patient participants and extracted relevant data for this study. DN led rapid reviews on predictive value of signs and symptoms. SdeL and BD contributed to data

For peer review only - http://bmjopen.bmj.com/site/about/guidelines.xhtml

interpretation and analysis, especially in relation to coding and linkage of electronic data, and design of the validation study. All authors checked and approved the final manuscript.

Consent for publication

Not applicable.

Availability of data and materials

All relevant data are included in the appendices.

Competing interests

MD's company makes templates for general practice record systems. All other authors declare no competing interests.

Funding statement

The RECAP study was funded primarily from a UK Research and Innovation grant 'Remote By Default' under the Covid-19 Emergency Fund. Additional funding for some staff salaries came from National Institute for Health Research (BRC-1215-20008) and Wellcome Trust (WT104830MA). Development of the coding templates was part funded by the Community Jameel Imperial College COVID-19 Excellence Fund.

Acknowledgements

We thank the patients and clinicians who participated in the study at a particularly busy and stressful time. James Ray gave general advice and suggested the RECAP acronym. Emma Ladds and Sietse Wieringa undertook additional interviews and focus groups and provided data extractions of relevant material from this wider dataset. Emma and Ella Mi assisted with the SNOMED coding.

For peer review only - http://bmjopen.bmj.com/site/about/guidelines.xhtml

Data sharing

Additional data, including an extensive summary of qualitative findings, is supplied as appendices. Researchers seeking further raw data should contact the corresponding author.

Patient and public involvement statement

Several of the research team are clinicians who developed Covid-19 and thus bring lived experience of the condition. The study was overseen by an independent advisory group with a lay chair and a separate patient advisory group consisting of people from diverse social and ethnic backgrounds who have had Covid-19; this group had input to the study design. The experience of patients was also captured systematically through patient interviews as an integral component of the research, and this led to amendments to the RECAP score. Some patient participants were recruited by snowballing from other patient participants and some from a patient support group. Results will be disseminated to all patient participants by emailing or posting a lay summary.

For peer review only

For peer review only - http://bmjopen.bmj.com/site/about/guidelines.xhtml

Additional material

Filename	Additional File 1 Focus group prompts
File format	pdf
Title of data	Focus group prompt questions
Description of data	Focus group prompt questions

Filename	Additional File 2 Qualitative comments
File format	pdf
Title of data	Qualitative comments during Delphi process
Description of data	Examples of qualitative comments made in Delphi exercise

Filename	Additional File 3 Summary of vignette study
File format	pdf
Title of data	Summary of vignette study
Description of data	Methods and findings from vignette sub-study of Delphi process

Filename	Additional File 4 RECAP-0 full
File format	pdf
Title of data	Full version of RECAP-0 score
Description of data	RECAP-v0 score with full detail of how to complete each item

For peer review only - http://bmjopen.bmj.com/site/about/guidelines.xhtml

References

1. Wu Z, McGoogan JM. Characteristics of and important lessons from the coronavirus disease 2019 (COVID-19) outbreak in China: summary of a report of 72 314 cases from the Chinese Center for Disease Control and Prevention. *JAMA* 2020;323:1239-42.
2. Cohen P, Blau J. Coronavirus disease 2019 (COVID-19): Outpatient management in adults. UpToDate: Wolters Kluwer 2020 (Last updated 28th May). Accessed 31st May 2020 at https://www.uptodate.com/contents/coronavirus-disease-2019-covid-19-outpatient-management-in-adults.
3. Sperrin M, Grant SW, Peek N. Prediction models for diagnosis and prognosis in Covid-19: British Medical Journal Publishing Group, 2020.
4. Wynants L, Van Calster B, Bonten MM, et al. Prediction models for diagnosis and prognosis of covid-19 infection: systematic review and critical appraisal. *bmj* 2020;369
5. Nunan D, Brassey J, Mahtani K, et al. COVID-19 Signs and Symptoms Tracker. Oxford: Centre for Evidence-Based Medicine 2020. Accessed 10th June 2020 at https://www.cebm.net/covid-19/covid-19-signs-and-symptoms-tracker/.
6. Greenhalgh T, Koh GCH, Car J. Covid-19: a remote assessment in primary care. *Bmj* 2020;368:m1182. doi: 10.1136/bmj.m1182 [published Online First: 2020/03/28]
7. Royal College of General Practitioners. Using the National Early Warning Score (NEWS2) in Primary Care. London: RCGP 2020. Accessed 8th April 2020 at https://elearning.rcgp.org.uk/mod/page/view.php?id=10568.
8. Royal College of Physicians (UK). National Early Warning Score (NEWS) 2. London: RCP 2017. Accessed 5th April 2020 at https://www.rcplondon.ac.uk/projects/outputs/national-early-warning-score-news-2.
9. National Institute for Health and Clincial Excellence. COVID-19 rapid guideline: managing suspected or confirmed pneumonia in adults in the community. London: NICE 2020. Accessed 4th April 2020 at https://www.nice.org.uk/guidance/ng165.
10. Carr E, Bendayan R, Bean D, et al. Supplementing the National Early Warning Score (NEWS2) for anticipating early deterioration among patients with COVID-19 infection. *medRxiv* 2020
11. Shea BJ, Reeves BC, Wells G, et al. AMSTAR 2: a critical appraisal tool for systematic reviews that include randomised or non-randomised studies of healthcare interventions, or both. *bmj* 2017;358:j4008.
12. Boulkedid R, Abdoul H, Loustau M, et al. Using and reporting the Delphi method for selecting healthcare quality indicators: a systematic review. *PloS one* 2011;6(6)
13. Khodyakov D, Chen C. Response Changes in Delphi Processes: Why Is It Important to Provide High-Quality Feedback to Delphi Participants? *Journal of Clinical Epidemiology* 2020
14. Belton I, MacDonald A, Wright G, et al. Improving the practical application of the Delphi method in group-based judgment: A six-step prescription for a well-founded and defensible process. *Technological Forecasting and Social Change* 2019;147:72-82.
15. Kitzinger J. The methodology of focus groups: the importance of interaction between research participants. *Sociology of health & illness* 1994;16(1):103-21.
16. Wilkinson S. Focus group methodology: a review. *International journal of social research methodology* 1998;1(3):181-203.
17. Abrams KM, Gaiser TJ. Online focus groups. *The SAGE handbook of online research methods* 2017:435-50.
18. Ritchie J, Spencer L. Qualitative data analysis for applied policy research. Analyzing qualitative data: Routledge 2002:187-208.
19. Greenhalgh T, Kotze K, Van Der Westhuizen H-M. Are there any evidence-based ways of assessing dyspnoea (breathlessness) by telephone or video? : Oxford COVID-19 Evidence Service Rapid Review. 30th March 2020. Accessed 1.4.20 at https://www.cebm.net/covid-19/are-there-any-evidence-based-ways-of-assessing-dyspnoea-breathlessness-by-telephone-or-video/.
20. Greenhalgh T. Should the Roth score be used in the remote assessment of breathlessness? Oxford: Oxford COVID-19 Evidence Service. Accessed 30th May 2020 at https://www.cebm.net/covid-19/roth-score-not-recommended-to-assess-breathlessness-over-the-phone/ 2020.
21. Greenhalgh T, Javid B, Knight M, et al. What is the efficacy and safety of rapid exercise tests for exertional desaturation in covid-19? Oxford: Oxford COVID-19 Evidence Service. Accessed 30th May 2020 at

For peer review only - http://bmjopen.bmj.com/site/about/guidelines.xhtml

https://www.cebm.net/covid-19/what-is-the-efficacy-and-safety-of-rapid-exercise-tests-for-exertional-desaturation-in-covid-19/. 2020.
22. Tarassenko L, Greenhalgh T. Should smartphone apps be used as oximeters? Oxford: Oxford COVID-19 Evidence Service. Accessed 30th May 2020 at https://www.cebm.net/covid-19/question-should-smartphone-apps-be-used-as-oximeters-answer-no// 2020.
23. Tarassenko L, McManus R, Greenhalgh T. Is the 'Lifelight' app adequately validated for blood pressure measurement? Oxford: Oxford COVID-19 Evidence Service. Accessed 30th May 2020 at https://www.cebm.net/covid-19/question-is-the-lifelight-app-adequately-validated-for-blood-pressure-measurement-answer-no/. 2020.
24. Chorin E, Padegimas A, Havakuk O, et al. Assessment of Respiratory Distress by the Roth Score. *Clinical cardiology* 2016;39(11):636-39.
25. Guan W-j, Ni Z-y, Hu Y, et al. Clinical characteristics of coronavirus disease 2019 in China. *New England Journal of Medicine* 2020
26. Menni C, Valdes AM, Freidin MB, et al. Real-time tracking of self-reported symptoms to predict potential COVID-19. *Nature medicine* 2020:1-4.
27. Gerry S, Bonnici T, Birks J, et al. Early warning scores for detecting deterioration in adult hospital patients: systematic review and critical appraisal of methodology. *bmj* 2020;369:m1501.
28. Inada-Kim M. NEWS 2: An opportunity to standardise the management of deterioration and sepsis *BMJ (blog)* 2018;8th February. Accessed 4th April 2020 at http://blogs.bmj.com/bmj/2018/02/08/news-2-an-opportunity-to-standardise-the-management-of-deterioration-and-sepsis/
29. National Institute for Health and Clincial Excellence. National Early Warning Score systems that alert to deteriorating adult patients in hospital (Medtech innovation briefing 205). LOndon: NICE 2020.
30. National Institute for Health and Clincial Excellence. COVID-19 rapid guideline: critical care in adults. Accessed 4th April 2020 at https://www.nice.org.uk/guidance/ng159/resources/covid19-rapid-guideline-critical-care-pdf-66141848681413. London: NICE 2020.
31. Patel R, Nugawela MD, Edwards HB, et al. Can early warning scores identify deteriorating patients in pre-hospital settings? A systematic review. *Resuscitation* 2018;132:101-11.
32. Grant S, Crimmons K. Limitations of track and trigger systems and the National Early Warning Score. Part 2: sensitivity versus specificity. *Br J Nurs* 2018;27(12):705-10. doi: 10.12968/bjon.2018.27.12.705 [published Online First: 2018/06/29]
33. Grant S. Limitations of track and trigger systems and the National Early Warning Score. Part 3: cultural and behavioural factors. *Br J Nurs* 2019;28(4):234-41. doi: 10.12968/bjon.2019.28.4.234 [published Online First: 2019/02/28]
34. Grant S. Limitations of track and trigger systems and the National Early Warning Score. Part 1: areas of contention. *Br J Nurs* 2018;27(11):624-31. doi: 10.12968/bjon.2018.27.11.624 [published Online First: 2018/06/13]
35. Pullyblank A, Tavaré A, Little H, et al. Implementation of the National Early Warning Score in patients with suspicion of sepsis: evaluation of a system-wide quality improvement project. *British Journal of General Practice* 2020;70(695):e381-e88.
36. Roland DT, Rowland A, Cotterill S, et al. Fine balance between pragmatism and rigidness in system approaches to acute care. *Bmj* 2019;367:l6226.
37. Scott LJ, Redmond NM, Garrett J, et al. Distributions of the National Early Warning Score (NEWS) across a healthcare system following a large-scale roll-out. *Emerg Med J* 2019;36(5):287-92.
38. Stow D, Barker RO, Matthews FE, et al. National Early Warning Scores (NEWS/NEWS2) and COVID-19 deaths in care homes: a longitudinal ecological study. *medRxiv* 2020
39. Rice B, Leanza J, Mowafi H, et al. Defining high-risk emergency chief complaints: data-driven triage for low-and middle-income countries. *Academic Emergency Medicine* 2020
40. Knight SR, Ho A, Pius R, et al. Risk stratification of patients admitted to hospital with covid-19 using the ISARIC WHO Clinical Characterisation Protocol: development and validation of the 4C Mortality Score. *bmj* 2020;370
41. Collins GS, Reitsma JB, Altman DG, et al. Transparent reporting of a multivariable prediction model for individual prognosis or diagnosis (TRIPOD): the TRIPOD statement. *British Journal of Surgery* 2015;102(3):148-58.
42. Delaney B. RECAP project. London: Imperial College London. Accessed 3rd October 2020 at https://imperialbrc.nihr.ac.uk/research/covid-19/covid-19-ongoing-studies/recap/ 2020.
43. Chen T, Wu D, Chen H, et al. Clinical characteristics of 113 deceased patients with coronavirus disease 2019: retrospective study. *Bmj* 2020;368

For peer review only

44. Gattinoni L, Chiumello D, Caironi P, et al. COVID-19 pneumonia: different respiratory treatments for different phenotypes? *Intensive Care Medicine* 2020:1.
45. Ottestad W, Seim M, Mæhlen JO. COVID-19 with silent hypoxemia. *Tidsskrift for Den norske legeforening* 2020
46. Wilkerson RG, Adler JD, Shah NG, et al. Silent hypoxia: A harbinger of clinical deterioration in patients with COVID-19. *The American Journal of Emergency Medicine* 2020
47. Chauhan V, Galwankar SC, Yellapu V, et al. State of the globe: The trials and tribulations of the COVID-19 pandemic: Separated but together, telemedicine revolution, frontline struggle against “Silent Hypoxia,” the relentless search for novel therapeutics and vaccines, and the daunting prospect of “COVIFLU”. *Journal of Global Infectious Diseases* 2020;12(2):39.
48. Xie J, Tong Z, Guan X, et al. Critical care crisis and some recommendations during the COVID-19 epidemic in China. *Intensive care medicine* 2020:1-4.
49. Niazkar HR, Zibaee B, Nasimi A, et al. The neurological manifestations of COVID-19: a review article. *Neurological Sciences* 2020:1.
50. Smith D, Wyatt J, McLuckie A, et al. Severe exercise hypoxaemia with normal or near normal X-rays: a feature of Pneumocystis carinii infection. *The Lancet* 1988;332(8619):1049-51.
51. Zheng Z, Peng F, Xu B, et al. Risk factors of critical & mortal COVID-19 cases: A systematic literature review and meta-analysis. *Journal of Infection* 2020
52. Public Health England. Disparities in the risk and outcomes of COVID-19. . London: PHE Publications. Gateway number: GW-1311. Accessed 8th June 2020 at https://assets.publishing.service.gov.uk/government/uploads/system/uploads/attachment_data/file/890258/disparities_review.pdf 2020:354-360.
53. de Lusignan S, Dorward J, Correa A, et al. Risk factors for SARS-CoV-2 among patients in the Oxford Royal College of General Practitioners Research and Surveillance Centre primary care network: a cross-sectional study. *The Lancet Infectious Diseases* 2020

For peer review only

For peer review only - http://bmjopen.bmj.com/site/about/guidelines.xhtml

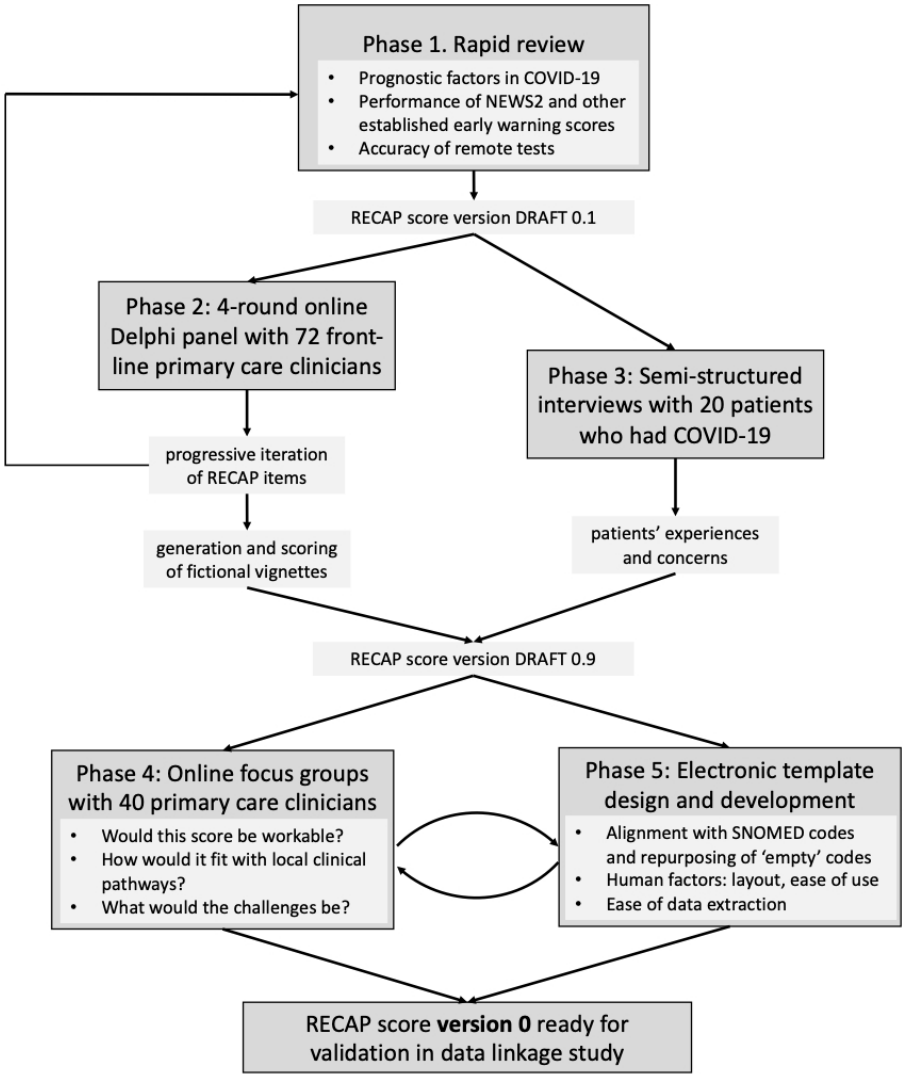

Figure 1: Study flowchart

RED ALERT CRITERIA: If patients have any of the following, consider 999

Adapted from national primary care covid-19 recommendations

Shock or peripheral shutdown

- Reduced level of consciousness
- Extremities – cold and clammy to touch
- Pallor – skin is mottled, ashen, blue or very pale
- Reduced urine output – little or no urine in last 24 hours

Severe breathlessness

- Rapid, significant deterioration in breathing in last hour
- New breathlessness at rest
- Newly unable to complete sentences
- Sudden onset of breathlessness

Other red flags which may be non-covid-19 related e.g.

- Severe central chest pain
- Collapse

RECAP-V0 SCORE FOR PATIENTS WHO DO NOT HAVE RED ALERT SYMPTOMS OR SIGNS

		Score 0	Score 1	Score 2	Score 3 => refer urgently	Score
1	Heart rate (per minute) *(if heart rate not available, score 1)*	51-90	41-50 or 91-110 or missing data	111-130	≤ 40 OR > 130, if unexplained	
2a	Shortness of breath	Not breathless at all	Breathless on moderate exertion e.g. walking room to room	Breathless on mild exertion e.g. getting out of a chair	Severe breathing difficulty; can't complete sentences at rest	Highest of 2a or 2b
2b	or Respiratory rate (per minute)	12-20	21-24	9-11 or 25-29	8 or less, or 30 or more	
3	Trajectory of breathlessness	Same or better than yesterday	Breathless, worse than yesterday	-	Significant deterioration in last hour	
4a	Oxygen saturation at rest	96% or above	95% (don't do 40-step test unsupervised)	94% (don't do 40-step test unsupervised)	93% or below (don't do 40-step test)	Highest of 4a, 4b & 4c
4b	or Saturation after 40 steps	Fall of 0-1%	-	Fall of 2%	Fall of 3% or more	
4c	or Profound tiredness or fatigue	None or mild	Noticeably more tired doing usual activities	Struggling to get out of bed	Unable to speak because of tiredness	
5a	Temperature	≤ 38 °C	38.1-39 °C	> 39 °C or < 35 °C	-	Highest of 5a or 5b
5b	or Feeling feverish with shivers	None	Feverish or chills	Uncontrollable shivering	-	
6	Time from first symptom (days)	7 or fewer	8 or more	-		
7	Muscle aches	None or mild	Moderate	Severe		
8	Cognitive decline	No	Less mentally alert than usual	New and worsening confusion	Reduced level of consciousness	
9	On COVID-19 shielded list (or has been inadvertently left off it)?	No	Yes	-	-	
10	Other risk factors for poor outcome? e.g. age, ethnicity	0-2	3 or more	-	-	
TOTAL						

RECAP score	Meaning	Recommended action
7 or more total or 3 on any item or extremely high level of clinical concern	HIGH RISK	Consider urgent referral
4-6 or more total or high level of clinical concern	MODERATE RISK	See in hot hub or virtual ward, or arrange home visit
0-3 total	LOW RISK	Advice and monitor at home

Figure 2: Summary of RECAP score

Additional File 1: Focus group prompt questions

1. How has your **experience managing COVID-19** been? **How do you make decisions about who to refer on**?
2. Do you **use any clinical decision score**? If so, which one?
3. What would a warning score **need to do for you**?
4. **Which patients does it need to select** for you?
5. How was your overall **experience using RECAP**?
6. Which were the main **positive aspects of RECAP**?
7. Did you have **any difficulties using RECAP**? Which?
    a. In particular, how does clinical uncertainty (i.e. difficulties assessing objective measures, such as HR) impact the application of RECAP?
    b. How do remote communication barriers (i.e. language, no video, poor phone line) impact the application of RECAP?
    c. Was time a problem? (i.e. Is it short enough to be useful in clinical practice?)
8. **How do you think these problems/difficulties could be eliminated or overcome**? Can you anticipate any possible solutions?
9. How do your **local and structural arrangements** influence your ability to apply RECAP?
10. Do you feel you need any additional information or training on how to use RECAP?
11. Is there anything else you want to add or feel we should have spoken about?
12. Demonstration of RECAP template using screen share.

***

Other possible questions to be discussed:

Are there any other ways to achieve #3?

Does RECAP seem capable to select those who would, or not benefit from oxygen therapy or heparin?

For peer review only

Additional File 2: Qualitative comments during Delphi process

Pulse rate

“We have noted that about 50% of our patients have BP monitors at home, which has been very helpful in our video consultations. In addition, we send out sats bus out to those patients we are concerned about, so have been able to include the PR [pulse rate] even in patients without.” [round 1]

“I think it [pulse rate] is really helpful in identifying sick young fit people who are otherwise compensating quite well and may have normal resting sats and a slightly high RR [respiratory rate] and significant fatigue and look ok. Less helpful in older adults unless very high. I have no idea of its predictive value for outcomes though.” [round 1]

“We have seen a lot of tachycardia in patients with milder symptoms so I think 2 rather than 3 is an appropriate score” [round 2]

“A bradycardia below 40 is an emergency whether or not a patient is on a B-blocker so the advice to adjust by 10 should not apply. Just because a patient uses a B blocker doesn’t mean they can tolerate a low cardiac output when septic/unwell.” [round 3]

“They [pulse rate cut-offs] don’t align with London guidance”

Temperature and symptoms of fever (chills, shivers)

“In my (pre-Covid) experience, patients are excellent at recognising rigors as being different from a normal high temperature.” [round 1]

“Temp 37.8 by NHS 111 is I think more about meeting clinical criteria for isolation, rather than because of any clinical utility. I think your cut offs are sensible.” [round 2]

“’use peak temperature before paracetamol’ implies a level of control that the clinician won’t have. Why not ’highest recorded temperature in the last 24 hours’ or similar?” [round 3]

“I would just score 1 for a temperature, rather than the height of the temperature” [round 3]

“If you mean ’rigors’ then the orange box should have the word ’rigors’ in it. Shivers and chills aren’t rigors (I’ve had both). ‘Feverish or chills with uncontrollable shivering’ perhaps is closer to the implied meaning.” [round 3]

“I suspect that chills is a greater reflection of malaise and temperature than the height of pyrexia and therefore reflects greater illness (but I’m guessing!).” [round 3]

Respiratory rate

“We have found RR [respiratory rate] as a very helpful tool with our video consultations and are using it to determine which patients require home visit/sats bus/hub attendance, with the cut off of more than 24 and less than 9. We are requiring RR for all referrals to our hot hub.” [round 1]

“Below 9 to score two- no way! That’s a 999 emergency. I have MRCEM and was an intensivist for several years. A low resp rate scares me a lot more than a high one. A low RR tells you the patient is peri-arrest. A high RR is worrying but tells you that the patient is - for the moment - responding appropriately to their respiratory or metabolic problem. A low RR tells you that they are no longer able to do so and are about to die without

For peer review only

intervention (some exceptions, of course, e.g. BZD plus alcohol OD when people often maintain a scarily low RR without apparent significant compromise - but not relevant here)." [round 2]

"I would amend to 2 = 25-29 and score 3 if greater than or equal to 30, to align with CURB and other scores. A RR of 30 is pretty rare and severe." [round 2]

"Almost certainly a good sign but worried about its feasibility" [round 3]

Breathlessness and silent hypoxia

"We have seen many patients with silent hypoxia. These patients have tended to be extremely fatigued. Would you consider including a normal RR but with extreme fatigue as a score 3?" [round 1]

"In practice we have noticed that patients are not articulating their breathlessness well - asking them about how their symptoms (both breathlessness and fatigue) are affecting their usual activities has been key - struggling to get out of bed, for example, is often a very significant change, for others it's not being able to manage the hoovering, for all it's changes to their usual activities and routine" [round 1]

"Would be good to have something to try and capture the silent hypoxics - such as feeling more fatigued and exhausted on exertion? Which I have had patients describe? Or a feeling that breathing is laboured but not breathless. Like at altitude." [round 1]

"Persistent, progressive SOB is a red flag" [round 1]

"Listening to friends (sadly several) who have suffered with COVID and even been to ICU, lot of them were that tired that they could not even speak." [round 2]

"Maybe 2 and 3 are too close together? How about 0: as it is 1: new breathless on moderate exertion eg up stairs 2: Breathless on mild exertion eg walking across room. 3: unable to complete sentences or severe difficultly breathing" [round 2]

"Struggling to get out of bed might not be that unusual for some people!" [round 2]

Illness trajectory

"The patients I've been worried about tend to be pyrexial >38 in week 2" [round 1]

"From the various narratives on the illness I wonder about days since symptoms began - it seems that patients in the first 7 days of symptoms may be less likely to deteriorate, whilst those whose symptoms go on after this probably need assessment. Careful assessment of confusion feels important - for my phone calls I am spending time talking to patients to assess for any level of confusion. This for me is a 'sign of hypoxia' - but that is not flagged clearly enough in the above for my liking." [round 1]

"[Tiredness] varies so much on stage of illness. Those with mild symptoms all c/o feeling very tired in the first 5 days, often not able to get out of bed for a day or two. But can manage their ADL [activities of daily living]. Worsening fatigue around day 7-10, having initially been feeling better, is a much worse prognostic feature in my limited experience." [round 1]

Pulse oximetry and exertional desaturation

For peer review only

"I think the walk test is really important." [round 1]

"Validated scoring system already in place I ask patients to walk 40 steps and then check by video with hand in their chest easier to see and count and sees how they are on exertion in comparison to rest ." [round 1]

"Until we know more I'm not sure that we can leave patients with pre-existing normal lungs at home with a score of 93%- this may be the best early warning that we can find." [round 2]

"Always a problem what to do with chronic lung disease. 6% [correction for chronic lung disease] is quite steep (since obviously depends on degree of chronic hypoxia) but I don't have any more rational suggestion." [round 2]

"NB: pan-London respiratory guidance decided not to specify a number for desaturations in latest draft. I had to miss that meeting so not sure about the reasoning, but I think it was something along the lines of needing to use clinical judgement. Personally, I think 'clinical judgement' needs some guidance as this is a new disease entity, so strongly support having specific numbers on this to guide clinicians." [round 2]

Muscle aches

"This is interesting. I just have not spoken to or seen many people who complain of severe muscle pains at all. This might be because I am seeing people (in the community as a GP) who have mild to moderate disease, and those with really severe disease with severe myalgia bypass their GPs and go straight to hospital. If hospital colleagues are saying it does seem to predict severity then leave it in." [round 4]

"muscle pain has been very common in our unwell patients" [round 4]

Red flag signs

"Central cyanosis needs 999." [round 1; 30 similar comments]

Comorbidities and demographic risk factors

"3 for 61yo male with BMI of 32 is not equal to active cancer, immunosuppressed and high fever in week 2?" [round 2]

"If the other questions don't raise concerns then i don't want to send thousands of obese diabetic hypertensives with mild disease for assessment - I think they need to be given specific advice that they are at risk of becoming more unwell quickly - so please call back if any concerns" [round 2]

"I think the cut offs are perfectly rational, but the comorbidities are complicated and hypertension in particular." [round 2]

"All patients on shielded list and having 3 risk factors will score 4 meeting the criteria for F2F assessment even if they have no concerning clinical features. This means that any non-white male patient over 65 and on the shielding list needs a F2F assessment before we even start with the clinical assessment, which is not appropriate. So I think the scoring levels need to be reconsidered and reduced for this or 6b. My inclination would be to reduce this to a 1 if on list and leave 6b as it is." [round 3]

"I would have the top score as '4 or more'. A fit 68yr old Asian male with no other illnesses, or a white male with either well controlled hypertension or diabetes scoring 2 feels a little high." [round 3]

For peer review only

Other comments

"I don't trust China data" [round 1]

"I think you have covered the important things. The key here is to assess physiology. I think history is less important. The patients who become very unwell have a large systemic inflammatory response or severe pneumonia/pneumonitis so we need to focus on identify when people have SIRS or significant respiratory compromise." [round 1]

"I am very concerned about the application of clinical scores to patients that don't capture the individuality of patient circumstances - particularly if they are to be used to inform important decisions such as admission to hospital or deciding that a patient is in need of end of life care. There is always a danger that scores will be used to reduce and resolve these hugely complex issues - and are very clinician dependent." [round 1]

"I am not clear on the aim of the scoring system, and whether it acts as 'protocol' rather than a decision augmenting tool.. I hope you don't mind my honesty as I would like to help produce a simple, user-friendly diagnostic and prognostic tool.. but I think it needs to augment existing high level clinical skills rather than be another protocol to fill, creating over-reliance on this tool and cognitive distraction in the field. " [round 2]

"a really important part of our assessment in primary care is social circumstance and / or ability to mobilise community services. Lack of either of these can lead to admission too - while they may not be clinical "red flags" they may be worth including somehow as they contribute to the rationale for the decision to admit to hospital (and capture a little about the person being admitted)" [round 2]

"An observation. I realise that it is an unavoidable consequence of remote assessment but if you have a device to measure sats or pulse rate, you have 'more chance' of having a higher overall score." [round 3]

"There are too many questions if all included, so it is too easy to get a high score - need to reduce the number - could aim for 8 questions as in NEWS2? Would it be worth considering a table top exercise before sending out for initial testing? (eg give some scenarios & decide if admit or not & then do score to see if confirms decision or not?)." [round 3]

For peer review only

Additional File 3: Summary of vignette study

We used five vignettes, deliberately presented as ‘grey cases’ with considerable clinical, technical and social uncertainty (missing data, vague symptoms, potentially irrelevant material). In the first round of vignettes, we asked participants to score each item on the RECAP instrument, calculate the RECAP score and then say what they would recommend for the patient and whether the score reflected their clinical concern.

The 51 responses to this round showed surprising variation (e.g. the RECAP score varied from 2 to 16 in one vignette). However, free text comments suggested that the respondents were unsure how to calculate the score from the individual items (uncertainty which to add with ‘and’ and which to use as alternates with ‘or’, and whether to count the clinical concern within the RECAP score or separately from it).

We therefore ran a second round of vignettes (using three of the five), and using an automated scoring system. This produced 37 responses and a more consistent response, though still a fairly wide spread. Free text responses suggested that respondents found the vignettes time-consuming and somewhat confusing.

Details analysis is below.

VIGNETTE 1: Mrs Rahman

> *Mrs Rahman (South Asian ethnicity) is 78; she lives with her extended family who are concerned about her. She has had a dry cough and has felt hot for 6 days. Her appetite is poor (she has apparently lost her sense of taste). She spends most of her time in bed and shows no signs of getting better. When you try speak to her, you have no common language but her daughter-in-law says she is not short of breath, just very tired. She says her mother-in-law gets up to go to the toilet, and comes downstairs once a day to watch a TV programme. She says she doesn’t look blue or pale. She’s been hot and sweaty but has not had uncontrollable shivering, but she is aching quite badly in her back and down her legs, and is very worried. Mrs Rahman has well-controlled type 2 diabetes (on metformin). Her BMI is 27 and a recent BP was 130/85. She had stage 2 breast cancer 20 years ago but no recurrence. The family do not have a thermometer, blood pressure machine, oximeter or other equipment, and nobody has access to a video application.*

Scoring in round 1 (51 responses):

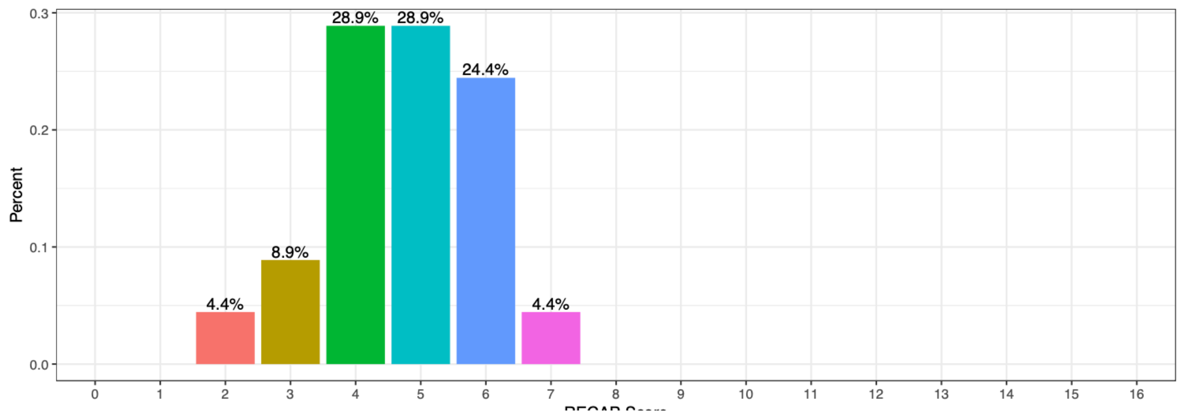

For peer review only

Scoring in round 2 (37 responses)

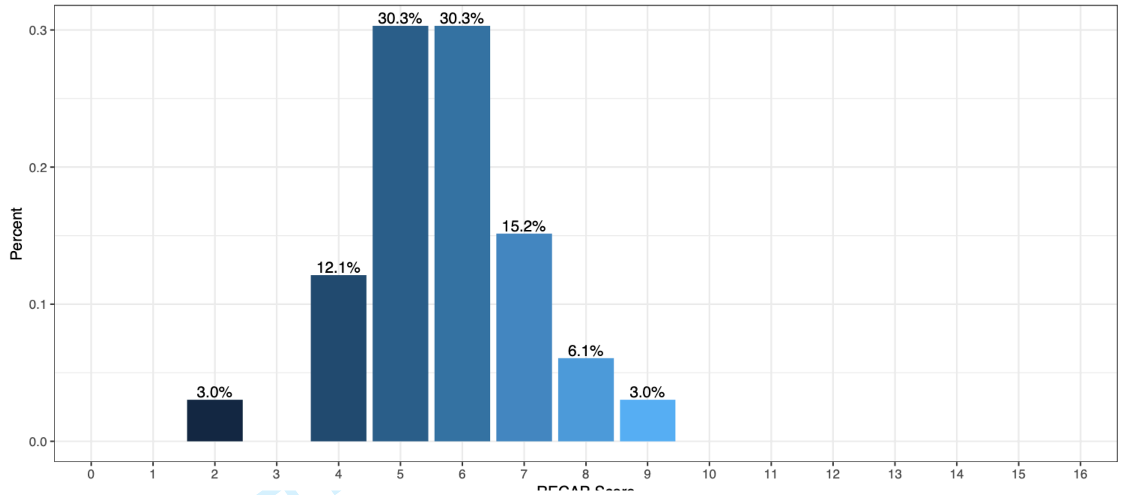

In sum, 82% of the first sample and 72% of the second sample would see in hot hub. 13% of first sample and 3% of second sample would reassure. Interestingly, 4% of the first sample and 24% of second sample would refer to hospital.

Responses to individual items showed that the main ones accounting for variation were

- Tiredness: Noticeably more tired doing usual activities (70%) versus struggling to get out of bed (30%)
- Fever: None (10%), feverish or chills (87%), feverish or chills with uncontrolled shivering (3%)
- Duration of temperature: not applicable (9%), fewer than 7 days (91%)
- Muscle aches: none (3%), moderate (61%), severe (36%)
- Is patient on shielded list? Yes 6%, no 94%.
- Risk factors for poor outcome? No 9%, yes 91%.
- Clinical concern: low 3%, moderate 70%, high 27%.
- Did RECAP reflect clinical concern? Yes 90% No 10%
- What would you do? Reassure 3%, see in hot hub 91%, refer urgently 6%

Qualitative comments:

Many said can't remember what the score is.

"It effectively has a bunch of 'not assessed' elements though, so the risk is that they cannot contribute to the score because missing, but missing does not equal 'not present' if there is a communication barrier (language but also no video or a poor phone line, or an inarticulate person who struggles to explain what they feel etc). I think it delivers the 'right' outcome though in the sense of face to face community review."

"Not clear how to score if no HR available Q4b) states feverish with shivers but then the score says "feverish or chills" scores 1 point... Q6b) not clear if I would have counted controlled hypertension as one factor, and also if I would use the BP cut off for diabetes in giving a point for diastolic >80... Scored 6 and then gave additional points for moderate worry = 8, which would result in urgent referral but I think I would have seen this patient in a hot site rather than admitting."

"Seeing her in the Red COVID Assessment Unit would feel right. Then we could assess parameters and see what her obs are."

Conclusion: The early warning score would prompt almost all GPs to assess this patient further. This seems appropriate given the multiple risk factors, missing data and language barrier.

For peer review only - http://bmjopen.bmj.com/site/about/guidelines.xhtml

VIGNETTE 2: Mr Jamieson

Mr Jamieson (African-Caribbean ethnicity) calls you urgently; he is 57 and lives with his wife. He has been unwell for 9 days, but only started feeling really bad yesterday. He has absolutely no energy and finds it hard to breathe, but is wondering if that's due to his asthma (for which he normally takes a Ventolin inhaler). He isn't out of breath at rest but was very short of breath brushing his teeth this evening, whereas this morning he could do that without a problem. He doesn't sound out of breath on the phone to you, and is able to complete sentences; an attempt at making a video connection fails. He says his peak flow rate is 425 (predicted for age 510; last asthma check 450). He forced himself to get up this morning, and has spent all day in a chair, finding it a struggle even to get to the toilet. He has a BP machine because he's on medication (amlodipine) for high blood pressure. Using his machine, his blood pressure today is 120/70 and pulse 105 (recent clinic values were 148/90 and 78). He's taken his temperature with a mouth thermometer he found in the cupboard but it only reads 35 even though he's sweated so much his wife had to change the sheets. When asked about feeling hot with shivering and chills, he says yes he's had all those, quite badly on one occasion. His muscles don't ache. He has sickle cell trait, but was told this would never cause him any problems. His BMI is 33, and a recent check-up showed an eGFR of 80.

Scoring in round 1 (51 responses):

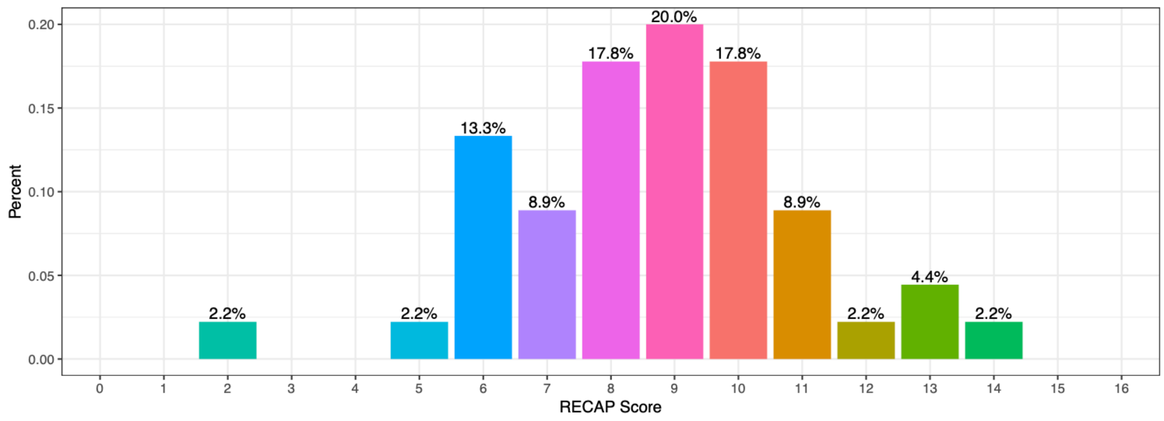

Scoring in round 2 (37 responses):

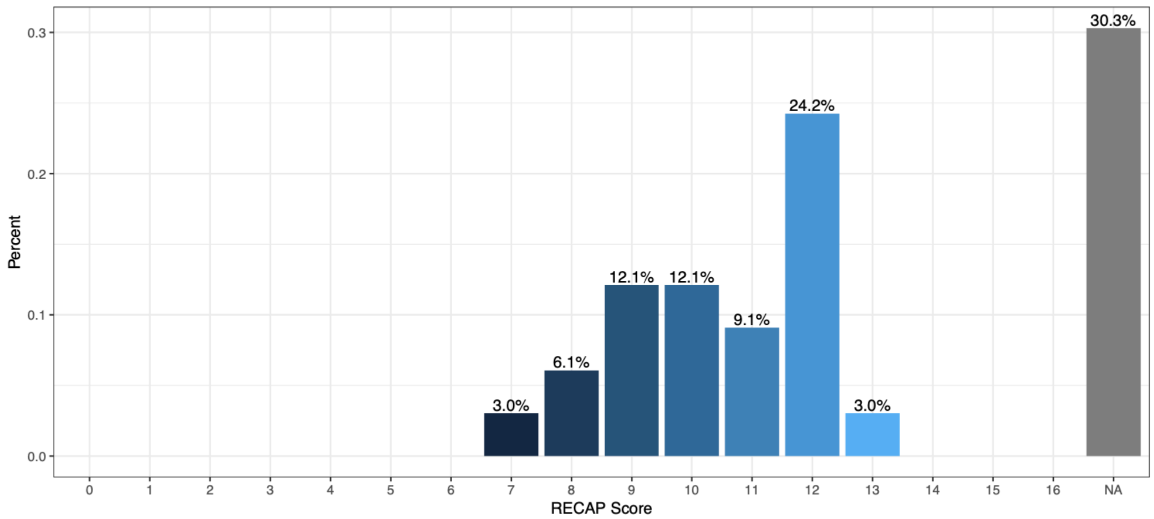

(the N/A column is because the patient has been referred urgently without completing the RECAP score)

For peer review only - http://bmjopen.bmj.com/site/about/guidelines.xhtml

In sum, 82% of first sample and 100% of second sample would refer this patient urgently.

Responses to individual items showed that the main discrepancies were

- Tiredness: Noticeably more tired doing usual activities (10%) versus struggling to get out of bed (90%)
- Shielded list yes 77% no 23%
- Duration of temp not applicable 42%, 8 or more days 45%
- Other risk factors for poor outcome 0-2 37%, 3 or more 63%
- Whether to refer urgently without completing RECAP or continue with RECAP
- Level of clinical concern: high 66% extremely high 31%
- Did RECAP capture your level of concern? Yes 77%, no 23% r1 and 90%, 10% r2.

Qualitative comments

Some wanted to ask him more questions about his asthma.

"Rapid deterioration in symptoms over 12 hours - high number of risks. Is at that 9-10 day tipping point for COVID - I would be admitting urgently."

"composite assessment - day 9, acute deterioration SOB, underlying asthma, risk factors. degree of SOB and deterioration is overriding concern"

"I think the profound fatigue is very concerning - I don't know that we are certain whether this is always a sign of silent hypoxia or whether it may just be due to immune response to SARS COV-2. I think I would want to check oxygen saturations, and most likely admit."

"Day 9, SOB brushing his teeth! Likely heading towards ARDS. Wouldn't want to waste time with Hub clinic with this one but would call him an ambulance."

"Although there is an argument (maybe) for see urgently in a hub - if his O2 sats and work of breathing are normal, what is hospital going to do? Xray? Rate of deterioration and duration of illness I think are the critical factors here that tip towards hospital assessment."

[The last comment above illustrates, I think, a GP who is less aware of the possibility of ARDS than the penultimate commenter above – ie RECAP score is budging this GP towards taking more aggressive action than they would otherwise have done – this is good!]

Conclusion: In every case in round 2 (where we calculated the score automatically), this high-risk patient would be referred urgently. The lower scores on round 1 may have been arithmetical or interpretation errors. Even in round 2, 31% of responders only had "moderate" or "high" clinical concern but would have been prompted to refer urgently.

VIGENTTE 3: Mrs Finlay

> *Mrs Finlay (White Irish ethnicity) is 88 and in a care home. She has multimorbidity (ischaemic heart disease, osteoarthritis, chronic pancreatitis and hypothyroidism) and on multiple medications, but her quality of life is good and she is normally mentally sharp. A recent conversation about 'ceiling of care' is documented in her medical record: she would like to be referred to hospital if appropriate. Her carers are concerned because after three days of a low-grade fever (37.5 on tympanic thermometer) and worsening breathlessness (which, since*

For peer review only - http://bmjopen.bmj.com/site/about/guidelines.xhtml

this morning, is present even when lying in bed), she has become delirious. Her blood pressure is 110/65 and pulse 120.

Scoring in round 1 (51 responses)

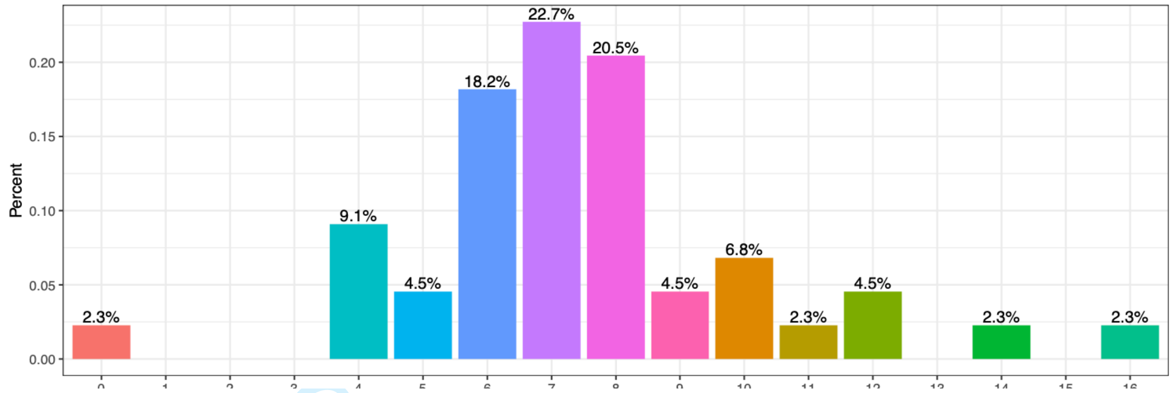

Responses to individual items showed that the main discrepancies were

- Clinical concern: moderate 7%, high 27%, extremely high 66%.
- Did RECAP reflect clinical concern? Yes 64% No 36%
- What would you do? See in hot hub 16%, refer urgently 82%

(Not included in round 2).
Qualitative comments:

"In practice, I would want a more detailed discussion with the carers about whether referral to hospital was "appropriate". Sounds like it should be, but hard to capture the nuance in a vignette. Unlike the one before or the one after, she is a hospital referral/999 as no point going to a community service if she's suitable for admission."

"Actually what I would do if at all possible is eyeball her (by video or in person) to establish if she is really delirious and very SOB. However, if I have to choose from only the 3 options above, I would have to admit. (Note: I am not sure whether you are using 'virtual ward' to include video consultation - this is not how we use it locally)."

"Given the delirium, and ceiling of care wishes, she needs an urgent work up."

"Delirium very difficult to score Also lots of causes of this in elderly eg UTI"

"Trajectory is important here, as is the new delirium and breathlessness at rest. Admit."

"RECAP itself didn't [capture my concern] (only 6, however does have 3 in one area)- however she has a red flag symptom - interestingly because of temperature/ no description of duration-/muscle aches etc, (really a lack of information as May happen in care home residents), the score is lower, but the presence of the red flag (confusion and new sobar) means that the score is less important - so overall the tool Accurately captured my concern."
[this DID capture the concern because RECAP says score 3 in one item → refer urgently]

Conclusion: This was the vignette with the most variation, due to two things: a) some GPs didn't see delirium as a red flag on the front page; b) some felt the delirium wasn't an indication to refer urgently (eg may have been a UTI). But more than 80% of GPs would refer given this story and score.

For peer review only

VIGNETTE 4: Mr Hughes

Mr Hughes is 72 (white British ethnicity) and lives alone. He has been unwell with Covid symptoms for 11 days. He was seen last week in the hot hub clinic because of breathlessness, and supplied with a home oximeter. Today, he feels more breathless than yesterday but says he can still walk around the living room. He's been measuring his saturations daily and the last three days have been 97%, 96%, 96%, but he feels much more tired today than he did yesterday. The oximeter measures his pulse at 98. Using a video app, you measure his respiratory rate at 24. You ask him to take 40 steps around his flat and re-measure his saturation levels. He does this, and says the level is still 96%, but a minute later it has fallen to 94%. His temperature with a mouth thermometer is 37.6 and he hasn't had shivers or chills. He had prostate cancer 10 years ago and was treated for depression last year after his wife died. His BMI is 28.

Scoring in round 1 (51 responses)

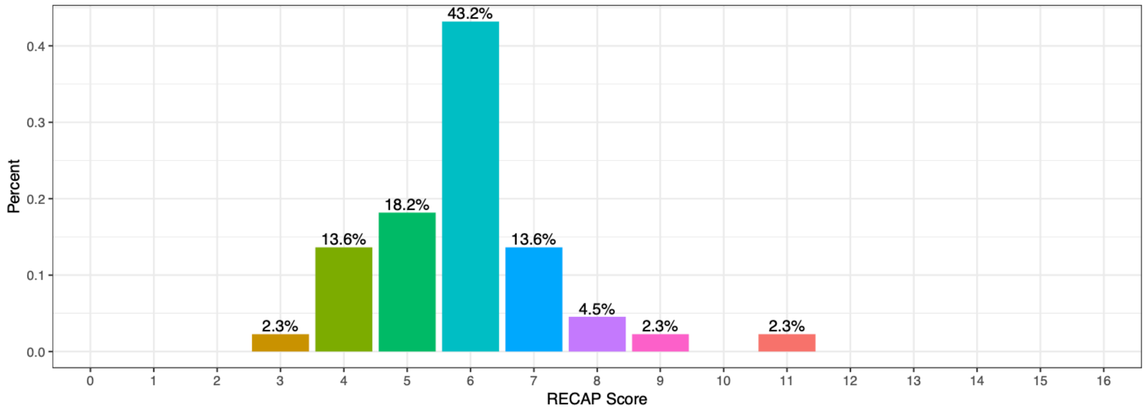

(no round 2 with this case)

In sum, 22% of this sample would refer this patient urgently; all but one responder would review in hot hub or at home.

Responses to individual items showed that the main discrepancies were

- Duration of temp: not applicable 42%, 8 or more days 45%
- Risk factors for poor outcome: 0-2 36%, 3 or more 64%
- Clinical concern: low 7% moderate 52% high 36% extremely high 5
- Did RECAP reflect clinical concern? Yes 80% No 20%
- What would you do? Reassure and advise 16%, See in hot hub 50%, refer urgently 32%

In sum, there was quite a bit of variation in what GPs subjectively felt was needed, but in all but one case, the patient would have had a hot hub review or hospital referral if score was followed.

Qualitative comments:

"Moderate risk - think the desaturations on walking are main red flag here"

"The post exertional hypoxia is a concern, and the isolation but there are psychosocial factors that might contribute to his malaise. He is not so ill that he needs hospital and a prompt Hub appointment could suffice."

"Deteriorating despite home management, desaturating, and vulnerable living alone. I'm not sure here what the hot hub would add to his assessment. I think he needs ED review."

For peer review only - http://bmjopen.bmj.com/site/about/guidelines.xhtml

“He already has a pulse oximeter. He will need daily calls but is probably safe to be managed remotely today.”

Conclusion: The spread here was because some GPs did not interpret exertional desaturation as a serious ‘red flag’ sign. The RECAP score pushed those GPs to reviewing the patient urgently or sending him to hospital. This is what the score is designed to do.

VIGNETTE 5: Mr Liu

Mr Liu is a 37-year old postgraduate student from Taiwan who lives in a shared house of multiple occupation. He’s worried because he has been unwell for 10 days and has a cough and a high fever (measured at 39 with a mouth thermometer). He’s also finding it hard to catch his breath – something that’s been going on for three or four days. He doesn’t think it’s worse today than yesterday. He doesn’t know his housemates well, but he knows two of them have been unwell. He is not registered with your practice so you have no previous records on him, but he tells you he might have been diagnosed with diabetes back in Taiwan, he’s not sure. His father had it and he is a bit overweight, though he doesn’t know his actual height and weight. He’s been lent an oximeter by one of his housemates and taken a reading – his pulse is 115 and saturation 95%. On a video call, you think he looks pale and anxious, and his respiratory rate is 22.

Scoring in round 1 (51 responses)

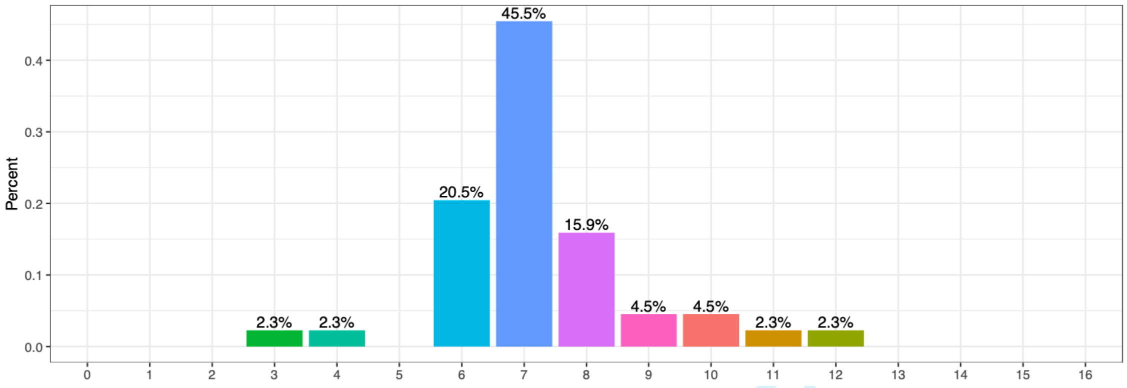

Scoring in round 2 (37 responses)

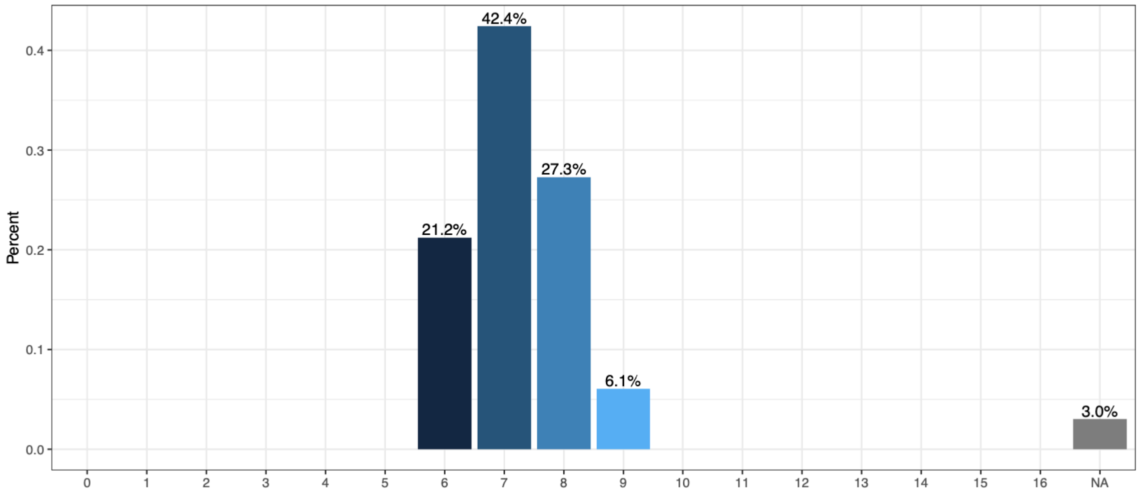

For peer review only - http://bmjopen.bmj.com/site/about/guidelines.xhtml

- Level of clinical concern r1 low 2%, moderate 52%, high 39%, extremely high 7%; r2 moderate 3%, high 66%, extremely high 31%.
- What would you do? R1 reassure and advise 5%, see in hot hub 65%, refer urgently 27%; R2 Reassure and advise 18%, See in hot hub 48%, refer urgently 33%
- Did score capture level of clinical concern r1 yes 80% no 20%; r2 90%, 10%.

In sum, 75% of r1 and 80% of r2 would have referred this man urgently.

Qualitative comments

“I think it came up higher than I would have expected.”

“score suggests consider urgent referral - well I’ve considered and am happy for him to be seen in hub. score would be higher still if he has DM and BMI >35 - which Ive not scored - but why he needs to be seen rather than have phone advice.”

“I think there’s a degree of uncertainty around this case. We don’t know the patient and there is some uncertainty regarding his PMH, but the safest thing is to assume he does have diabetes. It can be quite hard to differentiate between anxiety and an unwell patient over video especially, and paleness and tachycardia makes me concerned regarding shock. I would be admitting him.”

“During vignette, I was wondering would I really want to see this patient in a hot hub, to be knower, with Sats of 95 in a fit and well young man; that is worrying enough to me So yes I think overall though not so barn door, I feel direct referral to hospital is appropriate for him mainly given his sats/ duration etc”

Conclusion: This vague case had a number of potentially worrying features and inevitably they were interpreted slightly differently by the responders, but in every case the patient would have either been referred straight to hospital or seen in a hot hub. Once again, the score seems to be doing its job of prompting the GP to act on a sign or symptom they may not be aware is associated with poor prognosis.

ADDITIONAL COMMENTS

Complaints about layout and not seeing score in front of them.

Complaints about time it took.

--

TG, PT
1.6.20

For peer review only - http://bmjopen.bmj.com/site/about/guidelines.xhtml

For peer review only

Appendix A4: Full version of the RECAP-V0 score

REMOTE COVID-19 ASSESSMENT FOR PRIMARY CARE

This is a draft early warning score for use in a remote consultation. It is designed to support assessment of, and communication about, COVID-19 patients at the primary-secondary care interface. It is not intended to replace clinical judgement. It has been produced by professional consensus but has not yet been clinically validated.

If the RECAP score is high, it will not tell you whether to refer for active management (e.g. hospital or hot hub), or arrange palliative care (other factors will of course influence this decision).

RED ALERT CRITERIA: If patients have any of the following, consider 999

Adapted from national primary care covid-19 recommendations

Shock or peripheral shutdown
- New confusion (including delirium)
- Reduced level of consciousness
- Extremities – cold and clammy to touch
- Pallor – skin is mottled, ashen, blue or very pale
- Reduced urine output – little or no urine in last 24 hours

Severe breathlessness
- Rapid, significant deterioration in breathing in last hour
- New breathlessness at rest
- Newly unable to complete sentences
- Sudden onset of breathlessness

Other red flags which may be non-covid-19 related e.g.
- Severe central chest pain
- Collapse

RECAP-V0 SCORE FOR PATIENTS WHO DO NOT HAVE RED ALERT SYMPTOMS OR SIGNS

		Score 0	Score 1	Score 2	Score 3 => refer urgently	Score
1	Heart rate (per minute) *(if heart rate not available, score 1)*	51-90	41-50 or 91-110 or missing data	111-130	≤ 40 OR > 130, if unexplained	

Use medically approved device if available, or patient's own. Lower threshold for tachycardia by 10 bpm if beta-blocker or other heart-slowing drug taken in past 24h, but use standard thresholds for bradycardia. Adjust score if known to have physiological bradycardia (e.g. athlete).

		Score 0	Score 1	Score 2	Score 3 => refer urgently	Score
2a	Shortness of breath	Not breathless at all	Breathless on moderate exertion e.g. walking room to room	Breathless on mild exertion e.g. getting out of a chair	Severe breathing difficulty; can't complete sentences at rest	Highest of 2a or 2b
2b	or Respiratory rate (per minute)	12-20	21-24	9-11 or 25-29	8 or less, or 30 or more	

Score any breathlessness that patient or carer is concerned about. Take account of pre-existing conditions such as COPD. Assess respiratory rate by video, ask patient to place hand on chest. An anxious patient may be hyperventilating.

		Score 0	Score 1	Score 2	Score 3 => refer urgently	Score
3	Trajectory of breathlessness	Same or better than yesterday	Breathless, worse than yesterday	-	Significant deterioration in last hour	

Pay careful attention to a history of recent worsening of breathlessness, especially if this is what concerns patient or carer.

		Score 0	Score 1	Score 2	Score 3 => refer urgently	Score
4a	Oxygen saturation at rest	96% or above	95% (don't do 40-step test unsupervised)	94% (don't do 40-step test unsupervised)	93% or below (don't do 40-step test)	Highest of 4a, 4b & 4c
4b	or Saturation after 40 steps	Fall of 0-1%	-	Fall of 2%	Fall of 3% or more	
4c	or Profound tiredness or fatigue	None or mild	Noticeably more tired doing usual activities	Struggling to get out of bed	Unable to speak because of tiredness	

Patient should have warm hands and place oximeter device correctly. Lower thresholds if patient has chronic lung disease with known hypoxia (typically by 6%, but will vary – use usual baseline readings to adjust if known). Do exertion test only if clinician in attendance or if saturation is 96% or higher at rest (lower threshold for chronic lung disease). Saturation levels may fall for 1 min after stopping exercise. Most patients with covid-19 feel some fatigue, but profound fatigue may be a feature of 'silent hypoxia'. Take account of patient's baseline level of fatigue.

5a	Temperature	≤ 38 ºC	38.1-39 ºC	> 39 ºC<br>or < 35 ºC	-	Highest of 5a or 5b
5b	or Feeling feverish with shivers	None	Feverish or chills	Uncontrollable shivering	-	

A tympanic thermometer is preferred. Use highest recorded level in last 24 hours. A low reading may reflect user error.
If the patient has no reliable thermometer (or in addition to the temperature reading), explore for a description consistent with rigors.

6	Time from first symptom (days)	7 or fewer	8 or more	-		

Number of days since onset of first symptom

7	Muscle aches	None or mild	Moderate	Severe		

8	Cognitive decline	No	Less mentally alert than usual	New and worsening confusion	Reduced level of consciousness	

9	On COVID-19 shielded list (or has been inadvertently left off it)?	No	Yes	-	-	

Includes: • organ transplant • current chemotherapy or immunotherapy • severe lung condition such as cystic fibrosis • sickle cell anaemia • high dose steroids or other immunosuppressants • blood or bone marrow cancer • lung cancer on radiotherapy • splenectomy

10	Other risk factors for poor outcome in COVID-19?	0-2	3 or more	-	-	

For example: *• Age > 65 • BMI > 35 • male • non-White ethnicity • diabetes • hypertension • coronary heart disease • chronic kidney disease • vulnerable and isolated*

TOTAL	

Clinical concern component (be guided by clinical concern whatever the RECAP score; do not add this into the score but use it to over-ride the score if appropriate):

After assessing the patient, what is your level of clinical concern (regardless of RECAP score)?	Low	Moderate	High	Extremely high

RECAP score	Meaning	Recommended action
7 or more total or 3 on any item or extremely high level of clinical concern	HIGH RISK	Consider urgent referral
4-6 or more total or high level of clinical concern	MODERATE RISK	See in hot hub or virtual ward, or arrange home visit
0-3 total	LOW RISK	Advice and monitor at home

For peer review only

BMJ Open

What items should be included in an early warning score for remote assessment of suspected Covid-19? Qualitative and Delphi study

Journal:	*BMJ Open*
Manuscript ID	bmjopen-2020-042626.R2
Article Type:	Original research
Date Submitted by the Author:	03-Nov-2020
Complete List of Authors:	Greenhalgh, Trisha; University of Oxford, Nuffield Department of Primary Care Health Sciences<br>Thompson, Paul; University of Oxford, Department of Experimental Psychology<br>Weiringa, Sietse; University of Oxford, Nuffield Department of Primary Care Health Sciences<br>Neves, Ana Luisa; Imperial College London,<br>Husain, Laiba; University of Oxford, Nuffield Department of Primary Care Health Sciences<br>Dunlop, Merlin; Ardens Ltd<br>Rushforth, Alexander; University of Oxford, Nuffield Department of Primary Care Health Sciences<br>Nunan, David; University of Oxford, Nuffield Department of Primary Care Health Sciences<br>de Lusignan, Simon; Oxford University, Nuffield Department of Primary Care Health Sciences<br>Delaney, Brendan; Imperial College London, Surgery and Cancer
**Primary Subject Heading**:	Health services research
Secondary Subject Heading:	Infectious diseases, General practice / Family practice, Health informatics
Keywords:	INFECTIOUS DISEASES, Health informatics < BIOTECHNOLOGY & BIOINFORMATICS, VIROLOGY, COVID-19

SCHOLARONE™
Manuscripts

I, the Submitting Author has the right to grant and does grant on behalf of all authors of the Work (as defined in the below author licence), an exclusive licence and/or a non-exclusive licence for contributions from authors who are: i) UK Crown employees; ii) where BMJ has agreed a CC-BY licence shall apply, and/or iii) in accordance with the terms applicable for US Federal Government officers or employees acting as part of their official duties; on a worldwide, perpetual, irrevocable, royalty-free basis to BMJ Publishing Group Ltd ("BMJ") its licensees and where the relevant Journal is co-owned by BMJ to the co-owners of the Journal, to publish the Work in this journal and any other BMJ products and to exploit all rights, as set out in our licence.

The Submitting Author accepts and understands that any supply made under these terms is made by BMJ to the Submitting Author unless you are acting as an employee on behalf of your employer or a postgraduate student of an affiliated institution which is paying any applicable article publishing charge ("APC") for Open Access articles. Where the Submitting Author wishes to make the Work available on an Open Access basis (and intends to pay the relevant APC), the terms of reuse of such Open Access shall be governed by a Creative Commons licence – details of these licences and which Creative Commons licence will apply to this Work are set out in our licence referred to above.

Other than as permitted in any relevant BMJ Author's Self Archiving Policies, I confirm this Work has not been accepted for publication elsewhere, is not being considered for publication elsewhere and does not duplicate material already published. I confirm all authors consent to publication of this Work and authorise the granting of this licence.

For peer review only

Original research paper resubmitted to BMJ Open November 2020

What items should be included in an early warning score for remote assessment of suspected Covid-19? Qualitative and Delphi study

---

Trisha Greenhalgh (Professor)[1]

Paul Thompson (Researcher) [2]

Sietse Weiringa (Researcher)[1]

Ana Luisa Neves (Researcher)[3]

Laiba Husain (Researcher)[1]

Merlin Dunlop (GP)[4]

Alex Rushforth (Researcher)[1]

David Nunan (Researcher)[1]

Simon de Lusignan (Professor)[1]

Brendan Delaney (Professor)[3]

For peer review only - http://bmjopen.bmj.com/site/about/guidelines.xhtml

[1] Nuffield Department of Primary Care Health Sciences, University of Oxford, Oxford OX2 6GG, UK

[2] Department of Experimental Psychology, University of Oxford, Oxford OX2 6GG, UK

[3] Institute of Global Health Innovation, Imperial College London, London

[4] Ardens-Q Ltd

Corresponding author: Professor Trisha Greenhalgh, Nuffield Department of Primary Care Health Sciences, University of Oxford, Oxford OX2 6GG, UK - phone: +44 (0)1865 289294, email: trish.greenhalgh@phc.ox.ac.uk.

Keywords: Early warning score, RECAP (Remote Covid Assessment in Primary Care) score, Covid-19, Medical record systems, computerized.

Word count: 3956

For peer review only

ABSTRACT

Background

To develop items for an early warning score (RECAP: REmote Covid-19 Assessment in Primary Care) for patients with suspected Covid-19 who need escalation to next level of care.

Methods

The study was based in UK primary health care. The mixed-methods design included rapid review, Delphi panel, interviews, focus groups and software development. Participants were 112 primary care clinicians and 50 patients recovered from Covid-19, recruited through social media, patient groups and snowballing. Using rapid literature review, we identified signs and symptoms which are commoner in severe Covid-19. Building a preliminary set of items from these, we ran four rounds of an online Delphi panel with 72 clinicians, the last incorporating fictional vignettes, collating data on R software. We refined the items iteratively in response to quantitative and qualitative feedback. Items in the penultimate round were checked against narrative interviews with 50 Covid-19 patients. We required, for each item, at least 80% clinician agreement on relevance, wording and cut-off values, and that the item addressed issues and concerns raised by patients. In focus groups, 40 clinicians suggested further refinements and discussed workability of the instrument in relation to local resources and care pathways. This informed design of an electronic template for primary care systems.

Results

The pre-validation RECAP-V0 comprises a red flag alert box and 10 assessment items: pulse, shortness of breath or respiratory rate, trajectory of breathlessness, pulse oximeter reading (with brief exercise test if appropriate) or symptoms suggestive of hypoxia, temperature or fever symptoms, duration of symptoms, muscle aches, new confusion, shielded list, and known risk factors for poor outcome. It is not yet known how sensitive or specific it is.

For peer review only - http://bmjopen.bmj.com/site/about/guidelines.xhtml

Conclusions

Items on RECAP-V0 align strongly with published evidence, clinical judgement and patient experience. The validation phase of this study is ongoing.

Study registration

Clinicaltrials.gov (NCT04435041).

Article summary

Strengths and limitations of the study

- First systematic study to develop items for a Covid-19 early warning score for primary care
- Captures clinician and patient experience of the deteriorating patient with Covid-19
- Combines extensive qualitative research and quantitative consensus methodology
- Items have strong face validity but formal validation of the score is still ongoing
- RECAP is a severity prediction score, not a diagnostic score

For peer review only

Introduction

Some patients with Covid-19 experience deterioration (usually at around day 8).[1 2] There is therefore a need for research to develop accurate early warning scores – clinical prediction models designed to identify patients who need escalation to next level of care.[3] Such scores need to be both highly specific (detecting all patients who need onward referral) and fairly sensitive (excluding all or most patients who do not). A recent systematic review of prediction models for Covid-19 concluded that "proposed models are poorly reported, at high risk of bias, and their reported performance is probably optimistic" (page 2).[4] That review identified no evidence-based prediction models for primary care settings, nor did a citation-track of the article (which identified over 300 subsequent papers).

Assessment of a patient with suspected acute Covid-19 in primary care is fraught with uncertainty, since its clinical course differs from other pneumonias[5] and because most patients will be assessed remotely (i.e. by phone or video).[6] The UK Royal College of General Practitioners has cautiously endorsed the use of NEWS2 (National Early Warning Score 2) alongside clinical judgement for the assessment of patients with suspected acute Covid-19.[7] NEWS2 is calculated from the patient's temperature, pulse rate, respiratory rate, systolic blood pressure, pulse oximetry reading and new confusion.[8] The National Institute for Health and Care Excellence (NICE) rapid guideline on management of Covid-19 pneumonia in the community makes the guarded statement that the NEWS2 score "may be useful" in assessing deterioration but that the patient should not be brought in for a face-to-face assessment solely to calculate a NEWS2 score (paragraph 3.7).[9] But NEWS2 was developed for a different purpose (see Discussion), and requires data that may be difficult to obtain. A recent preprint suggest that it correlates poorly with severity of Covid-19.[10]

We sought to develop and validate a primary care early warning score for Covid-19 based on data that can be reliably collected during a remote consultation. This paper describes the development of items for a version 0 of RECAP which can be formally validated. It does not cover the actual validation of the instrument.

For peer review only - http://bmjopen.bmj.com/site/about/guidelines.xhtml

Method

Study design and governance

The study was part of the Remote By Default research programme, funded by UK Research and Innovation Covid-19 Emergency Research Fund. It consisted of five phases (Figure 1): rapid review, a four-round Delphi panel of primary care clinicians, semi-structured interviews and focus groups with patients, focus groups with primary care clinicians, and electronic template development. This in-depth qualitative design was chosen because of the novelty of the condition, the high degree of clinical uncertainty surrounding its acute management, and the added complexity of the need for remote assessment (which required judgements to be made without having fully examined the patient). For all these reasons, a detailed qualitative phase was considered essential before developing the score using actual outcome data and then undertaking a validation exercise. The study was overseen by an independent advisory group with a lay chair and a separate patient advisory group. Ethics approvals are included at the end of the paper.

FIGURE 1

Rapid review

Detailed methods have been published elsewhere [5]. Briefly, fortnightly keyword searches were conducted from March to June 2020 of PubMed for English-language systematic reviews and LitCovid and MedRxiv for reprints. Methodological quality of reviews was assessed using AMSTAR-2.[11] Primary studies within included reviews were cross-checked for duplication before extracting data on each symptom or sign in both mild and severe Covid-19. The synthesis was continually updated over the course of the study period from the most up-to-date, highest-quality systematic reviews. To construct the items for the draft RECAP score, we selected symptoms and signs that were significantly commoner in severe than mild disease.

For peer review only - http://bmjopen.bmj.com/site/about/guidelines.xhtml

Delphi panel

Delphi is a structured approach to working towards consensus.[12] Steps include defining a problem, selecting a panel of experts (both academics and clinicians), supplying evidence along with key uncertainties, collecting quantitative (numerical scores) and qualitative (free text) data on a set of statements, feeding scores and comments back to panel members, and repeating until residual disagreement cannot be resolved. Advantages of this method include practicality (it can be done online, asynchronously, without specialist tools), anonymity (participants know the average group score but not individuals' scores) and iteration (feedback prompts outliers to either defend their response to the group or change it).[13 14]

Following established methodology,[12] we used a social media announcement (TG's Twitter account) and snowballing from people thus recruited to obtain 72 volunteers (68 general practitioners, three nurse practitioners and one paramedic), all of whom were actively involved in assessing acutely ill patients with suspected Covid-19. Ten of these were clinical academics. The 'draft 0.1' RECAP items developed from rapid review was entered onto a questionnaire using Survey Monkey (homeworking during lockdown prevented us from accessing our usual professional survey software).

We sent brief instructions to participants, including a summary of what was known about prognostic factors in Covid-19. We gave them five days to complete the survey, scoring each item for relevance (should it be included?) and wording (how would they improve it?), and add free text comments. We sent two prompts to non-responders. A researcher (PT) imported the raw data into R and collated quantitative responses using simple descriptive statistics (percentage selecting, for example, yes as currently worded, yes but change wording, not sure, probably not and definitely not), and tabulated qualitative responses, which TG subsequently arranged under broad clinical categories and themes. We amended the draft RECAP items before circulating a summary of data, notes on changes and reasons for them, and the next iteration of the items. This was repeated for a second and third round.

For peer review only - http://bmjopen.bmj.com/site/about/guidelines.xhtml

The fourth round included only three residual items on which we had not yet achieved our goal of 80% consensus, plus a set of five fictional vignettes which incorporated various scenarios (such as missing data, untrustworthy technology, complex social circumstances, and comorbidities) that participants had proposed in their free text responses. They were asked to assign points to individual items and then calculate a simulated RECAP score for each of these five vignettes, and also indicate their level of clinical concern. This fourth round was repeated following a report from several participants that they had been unsure how to calculate the total (so had guessed); in the repeat round, we adjusted the software to add up the items automatically.

Patient interviews

UK-based survivors of Covid-19 were recruited using three methods: a social media call (again, TG's Twitter account), snowballing from those who responded (to access those not on social media), and an email sent from a support group for people with prolonged Covid-19 symptoms (LongCovidSOS).

AR conducted an initial round of 20 audio recorded interviews by telephone or MS Teams, taking verbal consent. Patients were asked to narrate their various contacts with healthcare services, including how symptoms were assessed by call handlers or clinicians. Relevant sections of interviews were transcribed and read independently by AR and TG. Early in the study, data were extracted into a word document and arranged into categories to correspond to the items in RECAP-V0, and any additional categories not included in those items. Later in the study, these data were combined with comments from 30 additional survivors of acute Covid-19 extracted from a larger dataset of interviews and focus groups. The data were formally coded in NVIVO software and checked against the final RECAP-V0 items to confirm that all relevant issues raised by patients had been captured.

Clinician focus groups

Focus groups are a qualitative method designed to capture both individual perspectives and group dynamics (such as empathy, humour and conflict).[15 16] There is an established

For peer review only - http://bmjopen.bmj.com/site/about/guidelines.xhtml

methodology for conducting them by video-conference.[17] A new sample of 40 primary care clinicians (28 general practitioners, 11 nurse practitioners, 1 paramedic) who regularly assess suspected Covid-19 patients was recruited by a social media invitation enhanced by snowballing. Focus groups were held by Zoom and lasted 90 minutes. Prior to the group, we circulated ground rules (e.g. about confidentiality) and invited participants to read and score the five vignettes to familiarise themselves with RECAP-V0. Each focus group was facilitated by two academic general practitioners (TG, ALN or SW) guided by a list of prompt questions (reproduced in Additional File 1 on bmj.com). Briefly, we sought their views on RECAP-V0 (including a mock-up of the electronic template), and how they felt it would align with local clinical presentations, care pathways, electronic templates, resources and workflows. We asked what challenges they anticipated with its implementation.

Focus groups were video-recorded with consent. They were not fully transcribed but the clinical researchers all listened independently and transcribed selected sections. This free text was organised thematically on an Excel spreadsheet using the Framework method.[18] Insights from focus groups were fed iteratively into the software design phase described below.

Electronic template design and development

MD and BD matched individual RECAP-0 items with existing SNOMED-CT codes where possible. A template was constructed using drop-down menus, and inserted as a supplement to the Ardens Covid-19 assessment template built for EMIS Web (Egton Medical Information Systems), and in the NW London Integrated Care COVID templates. A further round of review of these codes was conducted with input from NW London CCGs (S-J Knight) and Dr Simon Gordon.

For peer review only - http://bmjopen.bmj.com/site/about/guidelines.xhtml

Results

Rapid review

Five factors appeared to predict poor outcome in Covid-19: persistent fever, shortness of breath, low oxygen saturation, muscle aches, and certain comorbidities.[5] Patients with severe Covid-19 may develop shock (manifest, for example, as deteriorating conscious level, hypotension, and reduced urine output). Based on these findings, we constructed a preliminary list of 13 items. Later in the study, additional questions emerged which prompted new rapid reviews: assessment of breathlessness,[19 20] how to measure exertional desaturation accurately and safely in patients with suspected Covid-19,[21] and the reliability of smartphone oximeters[22] and smartphone blood pressure measuring apps.[23]

Delphi panel

The Delphi panel was conducted in April and May 2020. Round 1 had a sample of 69 clinicians; three more joined for rounds 2 to 4. Of these, 62, 53, 56 and 51 completed the surveys respectively – response rates of 90%, 74%, 78% and 71%. Progression towards quantitative consensus across the four rounds is shown in Table 1.

TABLE 1

In sum, at least 80% agreement on relevance, wording and value sets for severity was eventually achieved for eight items: pulse, temperature, symptoms of fever (for use particularly if the patient does not have a reliable thermometer), oxygen saturation level, tiredness (which, if severe, may indicate hypoxia and hence potentially substitute for an oximeter reading), muscle aches, and known risk factors (comorbidities). Additional items with moderate agreement included demographic risk factors (77% agreement), oxygen saturation level after a 40-step exercise test (75%), trajectory of breathlessness (73%) and duration of temperature (63%).

For peer review only

For peer review only - http://bmjopen.bmj.com/site/about/guidelines.xhtml

Our qualitative dataset from the Delphi panel included over 200 pages of comments. Key themes are summarised and illustrated in Additional File 2 on bmj.com. These, along with rapid review, allowed us to characterise the clinical features of the deteriorating Covid-19 patient in primary care (Box 1)

BOX 1

In addition to prompting new rapid reviews, the Delphi qualitative data shaped the development of the items in several ways. Particular forms of words (for example, to question patients about the severity and rate of deterioration of breathlessness) enabled us to refine our items and value sets. Participants alerted us to existing guidance and protocols used either nationally or locally (there was strong consensus that any new instrument should complement rather than replace these). Comments about missing or untrustworthy data when a patient was being assessed at home via telephone or video link prompted us to develop default value sets or alternative questions to compensate for such deficiencies. The free text comments included rich data, based on real clinical experiences, from which we were able to construct the vignettes used in discussions. Numerous comments on the practicalities of applying the potential instrument prompted us to set up focus groups to explore these operational challenges further.

The five fictional vignettes and the results of the simulated scoring exercise on these are reproduced in Additional File 3 on bmj.com. In sum, whilst there was considerable variation in the number of points given (for example, in whether clinicians judged an ill-defined set of symptoms as “moderately” or “severely” tired), in all five vignettes the simulated RECAP-V0 score as calculated appeared to prompt an appropriate and cautious response. For example, in a case of an elderly South Asian patient who spoke no English and with no equipment at home for the family to take measurements, all but one participant were prompted by the RECAP-V0 simulated result to assess the patient in a face-to-face encounter. In a case of an African Caribbean man with profound tiredness and rapidly worsening breathlessness in the second week of his illness, all participants were prompted to arrange urgent transfer to hospital. Free text comments in several vignettes indicated

For peer review only - http://bmjopen.bmj.com/site/about/guidelines.xhtml

that some respondents' level of clinical concern had not been especially high and that they were surprised that the RECAP-V0 simulated score was so high.

Patient interviews

Of the 20 patient interviews in our original sample, 15 included detailed descriptions of deteriorating symptoms in the acute phase of Covid-19. These included worsening breathlessness, inability to speak in sentences, profound fatigue without feeling short of breath, high temperature, and symptoms interpreted as anxiety but which may have been acute tachycardia. Patients also described being reassured by remote assessments using video examination and home monitoring equipment.

> *"… when I was speaking to them they were listening to me breathing and watching my chest and things. And they were seeing my breathing through the video link, wanting to see a clear vision of my chest area and counting my respiratory rate. And looking back when I spoke to them I was breathless. […] I've already got a pulse oximeter and a blood pressure machine. […] The pulse ox[imeter] was really good. What I had to do is when they video called I had to put it on my finger and wave it at the screen and they were able to monitor it."* *[patient NM1]*

Some patients described long waits to get through to NHS 111 (the English telephone triage service), being asked "tick box" questions by call handlers or clinicians, and feeling dismissed on the basis of such questions even when they were concerned about the extent and pace of their own (or a relative's) deterioration. For example:

> *"You can't make a diagnosis on the phone because you don't get to see the physical symptoms. They [clinicians] never saw the [patient's] rash, I described it but they didn't actually get to see it. They couldn't see she was sweating all around her hairline and her face was super pale. The overall picture they couldn't see – they just had written down some numbers." [relative of patient, HG1]*

For peer review only - http://bmjopen.bmj.com/site/about/guidelines.xhtml

The “exercise” being referred to in the quote below is probably the Roth score,[24] which is likely neither sensitive nor specific in the assessment of Covid-19 patients.[20]

> *“There was an exercise where I need to count to something a certain number of times. They did a small exercise. But now they know you can have severe hypoxia without clear shortness of breath when talking. That was the assessment. They said ‘I know it’s rough, soldier through. You’re 34 years old. There’s no point in escalating this.’” [patient SN1]*

These patient data helped us refine the wording of the questions on the RECAP-V0 items and also the clinical description in Box 1. In particular, the patient data emphasised the importance of developing the prediction instrument as an adjunct to expert clinical judgement and not as a substitute for it. Additional qualitative data from 30 further Covid-19 survivors confirmed these themes and identified one additional relevant finding: that mismatches between the clinician’s and patient’s assessment of severity occurred, and were sometimes attributed by the clinician to anxiety:

> *“He [call handler who was being advised by nurse] said you seem to be able to talk in complete sentences. I was concerned because I thought I couldn’t. I said I’m trying my best but I am struggling to talk, you might not hear it but it’s definitely been happening this evening. […]. And the nurse asked the call handler to ask me if I suffer from anxiety.”*

Focus groups

In focus groups, clinicians described Covid-19 as a disease with “surprises”. They were especially concerned with patients whose initial course was unremarkable but who subsequently deteriorated rapidly. They wanted an early warning score not primarily to identify “red flag” patients (whose need for urgent hospital referral they felt was usually obvious), but for assessing moderately sick patients and tracking their progress over time. They considered the RECAP items to have high face validity and to reflect their level of clinical concern on the vignettes. Some felt that RECAP would be less useful for assessing an

For peer review only - http://bmjopen.bmj.com/site/about/guidelines.xhtml

unwell patient who did not have Covid-19. All three groups proposed the same additional item (patient less alert than usual or new confusion), and there was also agreement on a minor change to one item (changing "duration of temperature" to "duration of symptoms"). Participants made suggestions for layout and ordering of items on the electronic template, emphasising simplicity, ease of use and "human factors".

Developing the RECAP items

Data from the rapid reviews, Delphi panel, patient interviews and clinician focus groups contributed to development and refinement of the items for RECAP-V0 (Figure 2) which will go forward for further development (to create a final set of items and scoring weights) and validation. RECAP-V0, which cannot yet be called a 'score', consists of 10 items, three of which include alternative options designed to enable assessment even in the absence of technologies (video camera, thermometer, oximeter). Further explanatory text is given in Additional File 4 on bmj.com.

FIGURE 2

Electronic template design and development

To make an electronic version of RECAP, we selected pre-coordinated terms that were uniformly present in EMIS and SystmOne EHR systems, so that the same terms could be used and exported for linkage nationwide. The template included a prompt to capture the patient's verbal consent (supported by a url for the information sheet) for data linkage (as the relevant SNOMED code bound to our unique NHS Clinical Research Network Portfolio Number), as well as data previous entered and saved as part of the standard Covid-19 assessment. Layout and ordering of items was adapted in response to focus group comments and insights. It proved impossible to identify suitable codes for a value set on severity of myalgia, so the final template only supports presence/absence of myalgia.

For peer review only - http://bmjopen.bmj.com/site/about/guidelines.xhtml

Discussion

Statement of principal findings

This mixed-method study has produced five key findings. First, we have developed, through consensus, a rich description of clinical deterioration in the primary care patient with Covid-19 (Box 1). Second, we have achieved very high agreement among 72 front-line primary care clinicians and academics for the inclusion of a particular wording on the following variables for inclusion in an early warning score: pulse rate (88% agreement), temperature (89%), fever symptoms (91%), respiratory rate (84%), shortness of breath (79%), pulse oximeter reading (80%), post-exercise fall in pulse oximeter reading (75%), tiredness (88%), muscle aches (82%), shielded list (86%), and other risk factors for poor outcome (77%), along with affirmation of these by a second sample of 40 clinicians, who added duration of symptoms and new confusion. Third, we have obtained a high level of agreement on the numerical or descriptive value sets for different signs and symptoms in each item (details in Table 1). Fourth, we have confirmed that the items reflect the concerns of patients. Finally, we have surfaced, and begun to address, some of the human factors and operational challenges of implementing RECAP-V0 in different primary care services and settings across the UK.

Strengths and limitations of the study

This is the first study systematically to capture the clinical experience and wisdom of front-line primary care practitioners, as well as the experience of patients, in relation to the assessment of the deteriorating Covid-19 patient who has not yet been referred to hospital. Early Chinese studies on Covid-19 included only hospital patients.[1 25] Other community-based studies identified symptoms (notably loss of smell) associated with Covid-19 but which lack prognostic significance.[26]

Our study was undertaken at speed, in the midst of the first wave of the pandemic, and was influenced by the practicalities of the UK National Health Service under unprecedented stress. Despite these pressures, we believe we have achieved a sufficiently large and diverse

For peer review only - http://bmjopen.bmj.com/site/about/guidelines.xhtml

sample of front-line practitioners and patients to be confident that the findings reflect current best practice and patient priorities.

The main limitation is that we have not yet validated the instrument (see 'Next steps' below). Another limitation is that one item relies partly on UK-specific data (specifically the 'shielded' category). This item is, however, readily adaptable to reflect categories of vulnerability or risk used in other countries. Because the validation phase is not yet complete, we do not yet have weightings assigned to different items and selection of cut-off levels.

Comparison with previous literature

To date, most early warning scores have been developed for use in hospital inpatients using routinely collected vital sign data.[27] In UK hospitals, the NEWS2 score has become a common language of sickness with positive implications for patient safety (especially in relation to sepsis).[28] NEWS2 is recommended by NICE guidelines both in general[29] and as a component of the critical care of Covid-19 patients,[30] though it has been heavily criticised even in hospital settings.[27 31-34] NEWS2 has been used in pre-hospital settings by ambulance crews[31] and in early detection of suspected sepsis in primary care[35]. However, it has not been formally validated in general practice,[36] so its sensitivity and specificity in that context are unknown. Its positive predictive value is low even in hospital and ambulance settings, and is likely to be even worse in primary care because serious illness is rare.[37] A rise in NEWS2 appears to be a relatively late indicator of deterioration, typically triggering only in the last 12 hours before transfer to critical care.[27] For all these reasons, NEWS2 might cause harm from both under- and over-referral in a primary care setting, though there is preliminary evidence that it may have some value for Covid-19 in care home residents, who tend to be sicker.[38]

The key differences between NEWS2 and RECAP-V0 are as follows. Whereas NEWS2 was designed to be calculated from observations taken in hospital by trained staff and is based solely on signs, RECAP is designed to be completed in primary care as part of a clinician-patient conversation along with a (limited) remote physical examination, and is based on

For peer review only - http://bmjopen.bmj.com/site/about/guidelines.xhtml

both symptoms and signs. With the exception of pulse rate (which defaults to a value of 1 if no reading is possible), all items requiring a physical examination include an alternative item based solely on symptoms. This will allow the clinician to populate the score even when the assessment is being done remotely and the patient is unable or unwilling to use equipment, thereby reducing the danger of missing data.[27]

Whilst we set out to develop the RECAP score as a disease-specific instrument, a reviewer of a previous draft of this paper suggested that it may prove useful in both Covid-19 and non-Covid-19 acute deterioration since (he hypothesised) most symptoms of acute deterioration are not disease-specific. He drew our attention to a new study from Uganda which identified 12 “high-risk chief complaints” in a pre-hospital population which were associated with increased acute mortality in the subsequent days.[39] Interestingly, one of these was “difficulty speaking” which had not previously been included as a red flag symptom or prognostic marker but which was prominent in our qualitative data.

Two recent publications describe the development and validation of an in-hospital severity score for suspected Covid-19.[40 41] The International Severe Acute Respiratory and emerging Infections Consortium Coronavirus Clinical Characterisation Consortium (ISARIC-4C) study included over 35,000 patients in the derivation dataset and over 22,000 in the validation dataset. The final 4C Mortality Score included eight variables, six of which (respiratory rate, conscious level, peripheral oxygen saturation, comorbidities, gender and age) overlap with items in RECAP-V0. The other two (urea and C-reactive protein) require a blood test. Unlike the 4C Mortality Score, RECAP-V0 includes several items based on (or substitutable with) the patient’s *subjective* symptoms including shortness of breath and muscle aches, both of which have been shown to correlate strongly with disease severity.[5] RECAP-V0 also includes heart rate, as well as items based on time course (persisting fever on day 8 and trajectory of breathlessness), which reflect the clinical judgement of our Delphi panel of clinicians. We do not yet know whether any or all of these additional items improves the predictive value of the RECAP score.

For peer review only - http://bmjopen.bmj.com/site/about/guidelines.xhtml

Next steps

The transparent reporting of a multivariable prediction model for individual prognosis or diagnosis (TRIPOD) statement[42] states that development of a prognostic model (of which an early warning score is one example) requires two phases: instrument development and instrument validation. The study reported here has ensured that the first component of instrument development has captured three important dimensions: the existing evidence base from the research literature; the experience and intuition of front-line primary care clinicians; and the experience of patients. The second component of instrument development is to collect and analyse data on these important dimensions of clinical observation.

In the next phase of this study (ongoing), we are completing development and validating the RECAP score using data linkage between general practice electronic records and national datasets (local data from North West London's iCare and nationally via the RCGP Research and Surveillance Centre) using the primary outcome of hospital admission and secondary outcomes of intensive care unit admission and death. Recruitment into that phase of the study has begun. Further details are obtainable from the RECAP study website.[43]

Because of the novelty of the disease and the urgency of the research question, we decided to place our interim findings in the public domain so as to allow other teams to test and improve the RECAP items in parallel with our own continuing research (rather than, as is more commonly the case, seeking to ensure that our own validation study is published first). We welcome offers of collaboration from established research teams.

Until the findings of the next phase of the study are published, the validity of this instrument is unknown. Even if RECAP proves sensitive and specific for identifying the need for urgent escalation of care, it should be noted that this instrument is a *severity prediction tool*, not a diagnostic tool. It does not include items which are highly specific for diagnosing Covid-19 but are not predictive of its severity (e.g. loss of smell), and it includes many items (such as standard red flag indicators) which are not specific for Covid-19.

For peer review only - http://bmjopen.bmj.com/site/about/guidelines.xhtml

Figure 1: Study flowchart

See separate file

Figure 2: Summary of RECAP-V0 items

See separate file

For peer review only

For peer review only - http://bmjopen.bmj.com/site/about/guidelines.xhtml

Table 1: Results of Delphi panel

Focus of item	Percentage agreeing with item as worded			
	Round 1<br>(n = 62)	Round 2<br>(n = 53)	Round 3<br>(n = 56)	Round 4<br>(n = 51)
Pulse rate (wording of item)<br>Cut-off values for each category	73%<br>71%	70%<br>77%	**88%**<br>**84%**	
Temperature (wording)<br>Cut-off values	58%<br>55%	75%<br>81%	**89%**<br>**89%**	
Duration of temperature* (wording)<br>Cut-off values	58%			**63%**<br>**76%**
Symptoms of fever (e.g. chills, shivers)<br>Cut-off values	29%<br>63%	75%<br>87%	**91%**<br>**95%**	
Respiratory rate (wording)<br>Cut-off values	61%<br>76%	85%<br>85%	**84%**<br>**91%**	
Shortness of breath (wording)<br>Cut-off values	39%<br>44%	75%<br>79%	**79%**<br>**93%**	
Trajectory of breathlessness<br>Cut-off values				**73%**<br>**67%**
Oxygen saturation level<br>Cut-off values	65%<br>65%	79%<br>85%	**80%**<br>**82%**	
Oxygen saturation level after exercise<br>Cut-off values	-	68%<br>85%	**75%**<br>**91%**	
Clinical suspicion of hypoxia<br>Cut-off values	47%<br>55%			
Tiredness (wording)<br>Cut-off values	42%<br>73%	83%<br>89%	**88%**<br>**84%**	
Muscle pains or aches<br>Cut-off values	66%<br>47%	-<br>-	64%<br>**79%**	**82%**
Risk factors (comorbidities)<br>Cut-off values	53%	57%	**86%**<br>**82%**	
Risk factors (demographic e.g. age, ethnicity)			**77%**<br>**86%**	
Indicators of shock (including conscious level, new confusion**, low or no urine output, cold and clammy, mottled skin)	61%	64%	Moved to 'red flag' box to align with national guidance	-
Other red flag symptoms e.g. central chest pain	73%	34%		
Clinical concern	74%	77%	**86%**	

* 'Duration of temperature' was changed to 'duration of symptoms' after focus group discussion, with 8 days seen as the cut-off for clinical concern

** New confusion was subsequently proposed as a separate RECAP item by focus group participants, who did not agree that it should be an automatic 'red flag' sign

For peer review only

For peer review only - http://bmjopen.bmj.com/site/about/guidelines.xhtml

Box 1: The clinical course of the deteriorating Covid-19 patient in primary care

Synthesised from our qualitative data, supplemented from published sources

Covid-19 may present in primary care as a viral upper respiratory tract infection (e.g. sore throat), lower respiratory tract infection (e.g. cough, fever and mild dyspnoea), influenza-like illness (with fever, chills, headache and myalgia), or gastro-intestinal illness (with abdominal pains, nausea and diarrhoea).[2 6] Most patients have a relatively mild, self-limiting illness, but an unknown proportion (perhaps 10%) deteriorate, usually in week 2. Certain symptoms common in week 1, such as cough, mild fatigue and anosmia, do not appear to have prognostic significance.[5 44]

It is important to date-stamp the onset of first symptoms.[6] Severe dyspnoea, especially at rest, may indicate progression of lung involvement. The trajectory of dyspnoea is important, as acute respiratory distress syndrome occasionally follows quickly from the onset of breathlessness.[45] Formal scores for assessing dyspnoea severity appear to have a significant false negative rate and should not be used.[20] A careful history, noting what the patient is able to do and what they cannot do today that they could do yesterday, is likely to be more important.[20] A patient's or carer's concern about the severity of breathlessness may be significant and should not be dismissed as "anxiety".

Pulse oximeter readings are extremely useful in assessing unwell patients with Covid-19, so long as the device is reliable (smartphone apps are inherently inaccurate and should not be used[22]) and the patient or a relative is capable and confident to use it. The finger must be warm. Whilst a low oximeter reading is concerning, a normal one should not necessarily reassure, as young fit patients in particular can compensate well in the early stages of deterioration.

So-called silent hypoxia, defined as the development of respiratory failure without the subjective perception of dyspnoea, is a recently-described feature of severe Covid-19 and appears to have a poor prognosis.[44 46-49] Anecdotal accounts suggest that in some patients, silent hypoxia may manifest as profound tiredness, but we could not find published research on this association. New confusion (especially in older patients with comorbidity) was considered by clinicians in our sample to be a poor prognostic sign in Covid-19, but at the time of writing, evidence for this is limited.[50]

Covid-19 lung damage tends to be manifest as a perfusion defect (i.e. difficulty transferring oxygen across the alveolar membrane) rather than a ventilatory defect (difficulty getting air to the alveoli, as in asthma).[45] This may explain why Covid-19 can behave similarly to pneumocystis pneumonia in producing a fall in pulse oximetry reading on exertion (or in the minutes following exertion).[21 51] Because of this, patients with suspected Covid-19 should not be subject to exercise testing unless there is a clinician present if their resting pulse oximetry reading is abnormal (below 96%).

An unwell patient may or may not have Covid-19. An overall assessment is needed using questions relating to (for example) hydration status, dizziness, falls, central chest pain, fall in blood pressure (if the patient has equipment at home), change in mental status (including lethargy, new confusion, difficulty in rousing), central cyanosis (e.g. blue lips), and severe reduction in urine output. For this reason, a standard 'red flag' checklist should be quickly reviewed in all unwell patients.

There are some well-established risk factors for developing Covid-19 and worse outcome (e.g. age, non-white ethnicity, high body mass index and comorbidities including cardiovascular disease, hypertension and active cancer).[52-54] The extent to which these risk factors should be applied to 'load the score' of a patient who appears to have a mild form of the disease is not yet known, especially since shielded patients are the ones for whom a hospital or clinic visit carries most risk.

For peer review only - http://bmjopen.bmj.com/site/about/guidelines.xhtml

Declarations

Ethics approvals and consent to participate

Ethical approval for the patient interviews was obtained from East Midlands – Leicester Central Research Ethics Committee (IRAS Project ID: 283196; REC ref 20/EM0128) on 4th May 2020. Ethical approval for the validation study (including holding the focus groups and building and installing the software on primary care record systems) was obtained from Greater Manchester Research Ethics Committee on 20th May 2020 (IRAS Project ID: 283024; REC ref 20/NW/0266) and subsequent amendments. In accordance with our ethics approvals, all patient participants gave written informed consent and all clinician focus group participants gave verbal consent which was recorded on the video. We sought advice from the chair of our local NHS Research Ethics Committee who considered that the online Delphi panel did not require formal ethical approval; consent from these participants was obtained by email.

Contributorship statement

TG conceptualised the study and oversaw all work packages described in the paper. She led or contributed to the various rapid reviews, recruited participants, led data analysis and summarising, and wrote the paper. PT designed and led the operational and quantitative aspects of the Delphi study and the quantitative anlyses. SW and ALN recruited participants, co-facilitated the focus groups and participated in data analysis and writing up. LH provided assistance with all aspects of the research. MD designed the templates for putting RECAP items on general practice electronic records and participated in focus groups. AR interviewed patient participants and extracted relevant data for this study. DN led rapid reviews on predictive value of signs and symptoms. SdeL and BD contributed to data interpretation and analysis, especially in relation to coding and linkage of electronic data, and design of the validation study. All authors checked and approved the final manuscript.

For peer review only - http://bmjopen.bmj.com/site/about/guidelines.xhtml

Consent for publication

Not applicable.

Availability of data and materials

All relevant data are included in the appendices.

Competing interests

MD's company makes templates for general practice record systems. All other authors declare no competing interests.

Funding statement

The RECAP study was funded primarily from a UK Research and Innovation grant 'Remote By Default' under the Covid-19 Emergency Fund. Additional funding for some staff salaries came from National Institute for Health Research (BRC-1215-20008) and Wellcome Trust (WT104830MA). Development of the coding templates was part funded by the Community Jameel Imperial College COVID-19 Excellence Fund.

Acknowledgements

We thank the patients and clinicians who participated in the study at a particularly busy and stressful time. James Ray gave general advice and suggested the RECAP acronym. Emma Ladds and Sietse Wieringa undertook additional interviews and focus groups and provided data extractions of relevant material from this wider dataset. Emma and Ella Mi assisted with the SNOMED coding.

Data sharing

Additional data, including an extensive summary of qualitative findings, is supplied as appendices. Researchers seeking further raw data should contact the corresponding author.

For peer review only - http://bmjopen.bmj.com/site/about/guidelines.xhtml

Patient and public involvement statement

Several of the research team are clinicians who developed Covid-19 and thus bring lived experience of the condition. The study was overseen by an independent advisory group with a lay chair and a separate patient advisory group consisting of people from diverse social and ethnic backgrounds who have had Covid-19; this group had input to the study design. The experience of patients was also captured systematically through patient interviews as an integral component of the research, and this led to amendments to the RECAP items. Some patient participants were recruited by snowballing from other patient participants and some from a patient support group. Results will be disseminated to all patient participants by emailing or posting a lay summary.

For peer review only - http://bmjopen.bmj.com/site/about/guidelines.xhtml

Additional material

Filename	Additional File 1 Focus group prompts
File format	pdf
Title of data	Focus group prompt questions
Description of data	Focus group prompt questions

Filename	Additional File 2 Qualitative comments
File format	pdf
Title of data	Qualitative comments during Delphi process
Description of data	Examples of qualitative comments made in Delphi exercise

Filename	Additional File 3 Summary of vignette study
File format	pdf
Title of data	Summary of vignette study
Description of data	Methods and findings from vignette sub-study of Delphi process

Filename	Additional File 4 RECAP-V0 full revised
File format	pdf
Title of data	Full version of RECAP-V0 items
Description of data	RECAP-V0 items with full detail of how to complete each item

For peer review only

References

1. Wu Z, McGoogan JM. Characteristics of and important lessons from the coronavirus disease 2019 (COVID-19) outbreak in China: summary of a report of 72 314 cases from the Chinese Center for Disease Control and Prevention. *JAMA* 2020;323:1239-42.
2. Cohen P, Blau J. Coronavirus disease 2019 (COVID-19): Outpatient management in adults. UpToDate: Wolters Kluwer 2020 (Last updated 28th May). Accessed 31st May 2020 at https://www.uptodate.com/contents/coronavirus-disease-2019-covid-19-outpatient-management-in-adults.
3. Sperrin M, Grant SW, Peek N. Prediction models for diagnosis and prognosis in Covid-19: British Medical Journal Publishing Group, 2020.
4. Wynants L, Van Calster B, Bonten MM, et al. Prediction models for diagnosis and prognosis of covid-19 infection: systematic review and critical appraisal. *bmj* 2020;369
5. Nunan D, Brassey J, Mahtani K, et al. COVID-19 Signs and Symptoms Tracker. Oxford: Centre for Evidence-Based Medicine 2020. Accessed 10th June 2020 at https://www.cebm.net/covid-19/covid-19-signs-and-symptoms-tracker/.
6. Greenhalgh T, Koh GCH, Car J. Covid-19: a remote assessment in primary care. *Bmj* 2020;368:m1182. doi: 10.1136/bmj.m1182 [published Online First: 2020/03/28]
7. Royal College of General Practitioners. Using the National Early Warning Score (NEWS2) in Primary Care. London: RCGP 2020. Accessed 8th April 2020 at https://elearning.rcgp.org.uk/mod/page/view.php?id=10568.
8. Royal College of Physicians (UK). National Early Warning Score (NEWS) 2. London: RCP 2017. Accessed 5th April 2020 at https://www.rcplondon.ac.uk/projects/outputs/national-early-warning-score-news-2.
9. National Institute for Health and Clincial Excellence. COVID-19 rapid guideline: managing suspected or confirmed pneumonia in adults in the community. London: NICE 2020. Accessed 30th October 2020 at https://www.nice.org.uk/guidance/ng165.
10. Carr E, Bendayan R, Bean D, et al. Supplementing the National Early Warning Score (NEWS2) for anticipating early deterioration among patients with COVID-19 infection. *medRxiv* 2020
11. Shea BJ, Reeves BC, Wells G, et al. AMSTAR 2: a critical appraisal tool for systematic reviews that include randomised or non-randomised studies of healthcare interventions, or both. *bmj* 2017;358:j4008.
12. Boulkedid R, Abdoul H, Loustau M, et al. Using and reporting the Delphi method for selecting healthcare quality indicators: a systematic review. *PloS one* 2011;6(6)
13. Khodyakov D, Chen C. Response Changes in Delphi Processes: Why Is It Important to Provide High-Quality Feedback to Delphi Participants? *Journal of Clinical Epidemiology* 2020
14. Belton I, MacDonald A, Wright G, et al. Improving the practical application of the Delphi method in group-based judgment: A six-step prescription for a well-founded and defensible process. *Technological Forecasting and Social Change* 2019;147:72-82.
15. Kitzinger J. The methodology of focus groups: the importance of interaction between research participants. *Sociology of health & illness* 1994;16(1):103-21.
16. Wilkinson S. Focus group methodology: a review. *International journal of social research methodology* 1998;1(3):181-203.
17. Abrams KM, Gaiser TJ. Online focus groups. *The SAGE handbook of online research methods* 2017:435-50.
18. Ritchie J, Spencer L. Qualitative data analysis for applied policy research. Analyzing qualitative data: Routledge 2002:187-208.
19. Greenhalgh T, Kotze K, Van Der Westhuizen H-M. Are there any evidence-based ways of assessing dyspnoea (breathlessness) by telephone or video? : Oxford COVID-19 Evidence Service Rapid Review. 30th March 2020. Accessed 1.4.20 at https://www.cebm.net/covid-19/are-there-any-evidence-based-ways-of-assessing-dyspnoea-breathlessness-by-telephone-or-video/.
20. Greenhalgh T. Should the Roth score be used in the remote assessment of breathlessness? Oxford: Oxford COVID-19 Evidence Service. Accessed 30th May 2020 at https://www.cebm.net/covid-19/roth-score-not-recommended-to-assess-breathlessness-over-the-phone/ 2020.
21. Greenhalgh T, Javid B, Knight M, et al. What is the efficacy and safety of rapid exercise tests for exertional desaturation in covid-19? Oxford: Oxford COVID-19 Evidence Service. Accessed 30th May 2020 at

For peer review only - http://bmjopen.bmj.com/site/about/guidelines.xhtml

https://www.cebm.net/covid-19/what-is-the-efficacy-and-safety-of-rapid-exercise-tests-for-exertional-desaturation-in-covid-19/. 2020.
22. Tarassenko L, Greenhalgh T. Should smartphone apps be used as oximeters? Oxford: Oxford COVID-19 Evidence Service. Accessed 29th October 2020 at https://www.cebm.net/covid-19/question-should-smartphone-apps-be-used-as-oximeters-answer-no// 2020.
23. Tarassenko L, McManus R, Greenhalgh T. Is the 'Lifelight' app adequately validated for blood pressure measurement? Oxford: Oxford COVID-19 Evidence Service. Accessed 30th May 2020 at https://www.cebm.net/covid-19/question-is-the-lifelight-app-adequately-validated-for-blood-pressure-measurement-answer-no/. 2020.
24. Chorin E, Padegimas A, Havakuk O, et al. Assessment of Respiratory Distress by the Roth Score. *Clinical cardiology* 2016;39(11):636-39.
25. Guan W-j, Ni Z-y, Hu Y, et al. Clinical characteristics of coronavirus disease 2019 in China. *New England Journal of Medicine* 2020
26. Menni C, Valdes AM, Freidin MB, et al. Real-time tracking of self-reported symptoms to predict potential COVID-19. *Nature medicine* 2020:1-4.
27. Gerry S, Bonnici T, Birks J, et al. Early warning scores for detecting deterioration in adult hospital patients: systematic review and critical appraisal of methodology. *bmj* 2020;369:m1501.
28. Inada-Kim M. NEWS 2: An opportunity to standardise the management of deterioration and sepsis *BMJ (blog)* 2018;8th February. Accessed 4th April 2020 at http://blogs.bmj.com/bmj/2018/02/08/news-2-an-opportunity-to-standardise-the-management-of-deterioration-and-sepsis/
29. National Institute for Health and Clincial Excellence. National Early Warning Score systems that alert to deteriorating adult patients in hospital (Medtech innovation briefing 205). LOndon: NICE 2020.
30. National Institute for Health and Clincial Excellence. COVID-19 rapid guideline: critical care in adults. Accessed 30th October 2020 at https://www.nice.org.uk/guidance/ng159/resources/covid19-rapid-guideline-critical-care-pdf-66141848681413. London: NICE 2020.
31. Patel R, Nugawela MD, Edwards HB, et al. Can early warning scores identify deteriorating patients in pre-hospital settings? A systematic review. *Resuscitation* 2018;132:101-11.
32. Grant S, Crimmons K. Limitations of track and trigger systems and the National Early Warning Score. Part 2: sensitivity versus specificity. *Br J Nurs* 2018;27(12):705-10. doi: 10.12968/bjon.2018.27.12.705 [published Online First: 2018/06/29]
33. Grant S. Limitations of track and trigger systems and the National Early Warning Score. Part 3: cultural and behavioural factors. *Br J Nurs* 2019;28(4):234-41. doi: 10.12968/bjon.2019.28.4.234 [published Online First: 2019/02/28]
34. Grant S. Limitations of track and trigger systems and the National Early Warning Score. Part 1: areas of contention. *Br J Nurs* 2018;27(11):624-31. doi: 10.12968/bjon.2018.27.11.624 [published Online First: 2018/06/13]
35. Pullyblank A, Tavaré A, Little H, et al. Implementation of the National Early Warning Score in patients with suspicion of sepsis: evaluation of a system-wide quality improvement project. *British Journal of General Practice* 2020;70(695):e381-e88.
36. Roland DT, Rowland A, Cotterill S, et al. Fine balance between pragmatism and rigidness in system approaches to acute care. *Bmj* 2019;367:l6226.
37. Scott LJ, Redmond NM, Garrett J, et al. Distributions of the National Early Warning Score (NEWS) across a healthcare system following a large-scale roll-out. *Emerg Med J* 2019;36(5):287-92.
38. Stow D, Barker RO, Matthews FE, et al. National Early Warning Scores (NEWS/NEWS2) and COVID-19 deaths in care homes: a longitudinal ecological study. *medRxiv* 2020
39. Rice B, Leanza J, Mowafi H, et al. Defining high-risk emergency chief complaints: data-driven triage for low-and middle-income countries. *Academic Emergency Medicine* 2020
40. Knight SR, Ho A, Pius R, et al. Risk stratification of patients admitted to hospital with covid-19 using the ISARIC WHO Clinical Characterisation Protocol: development and validation of the 4C Mortality Score. *bmj* 2020;370
41. Gupta RK, Harrison EM, Ho A, et al. Development and validation of the 4C Deterioration model for adults hospitalised with COVID-19. *medRxiv* 2020
42. Collins GS, Reitsma JB, Altman DG, et al. Transparent reporting of a multivariable prediction model for individual prognosis or diagnosis (TRIPOD): the TRIPOD statement. *British Journal of Surgery* 2015;102(3):148-58.
43. Delaney B. RECAP project. London: Imperial College London. Accessed 3rd October 2020 at https://imperialbrc.nihr.ac.uk/research/covid-19/covid-19-ongoing-studies/recap/ 2020.

For peer review only - http://bmjopen.bmj.com/site/about/guidelines.xhtml

44. Chen T, Wu D, Chen H, et al. Clinical characteristics of 113 deceased patients with coronavirus disease 2019: retrospective study. *Bmj* 2020;368
45. Gattinoni L, Chiumello D, Caironi P, et al. COVID-19 pneumonia: different respiratory treatments for different phenotypes? *Intensive Care Medicine* 2020:1.
46. Ottestad W, Seim M, Mæhlen JO. COVID-19 with silent hypoxemia. *Tidsskrift for Den norske legeforening* 2020
47. Wilkerson RG, Adler JD, Shah NG, et al. Silent hypoxia: A harbinger of clinical deterioration in patients with COVID-19. *The American Journal of Emergency Medicine* 2020
48. Chauhan V, Galwankar SC, Yellapu V, et al. State of the globe: The trials and tribulations of the COVID-19 pandemic: Separated but together, telemedicine revolution, frontline struggle against "Silent Hypoxia," the relentless search for novel therapeutics and vaccines, and the daunting prospect of "COVIFLU". *Journal of Global Infectious Diseases* 2020;12(2):39.
49. Xie J, Tong Z, Guan X, et al. Critical care crisis and some recommendations during the COVID-19 epidemic in China. *Intensive care medicine* 2020:1-4.
50. Niazkar HR, Zibaee B, Nasimi A, et al. The neurological manifestations of COVID-19: a review article. *Neurological Sciences* 2020:1.
51. Smith D, Wyatt J, McLuckie A, et al. Severe exercise hypoxaemia with normal or near normal X-rays: a feature of Pneumocystis carinii infection. *The Lancet* 1988;332(8619):1049-51.
52. Zheng Z, Peng F, Xu B, et al. Risk factors of critical & mortal COVID-19 cases: A systematic literature review and meta-analysis. *Journal of Infection* 2020
53. Public Health England. Disparities in the risk and outcomes of COVID-19. . London: PHE Publications. Gateway number: GW-1311. Accessed 8th June 2020 at https://assets.publishing.service.gov.uk/government/uploads/system/uploads/attachment_data/file/890258/disparities_review.pdf 2020:354-360.
54. de Lusignan S, Dorward J, Correa A, et al. Risk factors for SARS-CoV-2 among patients in the Oxford Royal College of General Practitioners Research and Surveillance Centre primary care network: a cross-sectional study. *The Lancet Infectious Diseases* 2020

For peer review only - http://bmjopen.bmj.com/site/about/guidelines.xhtml

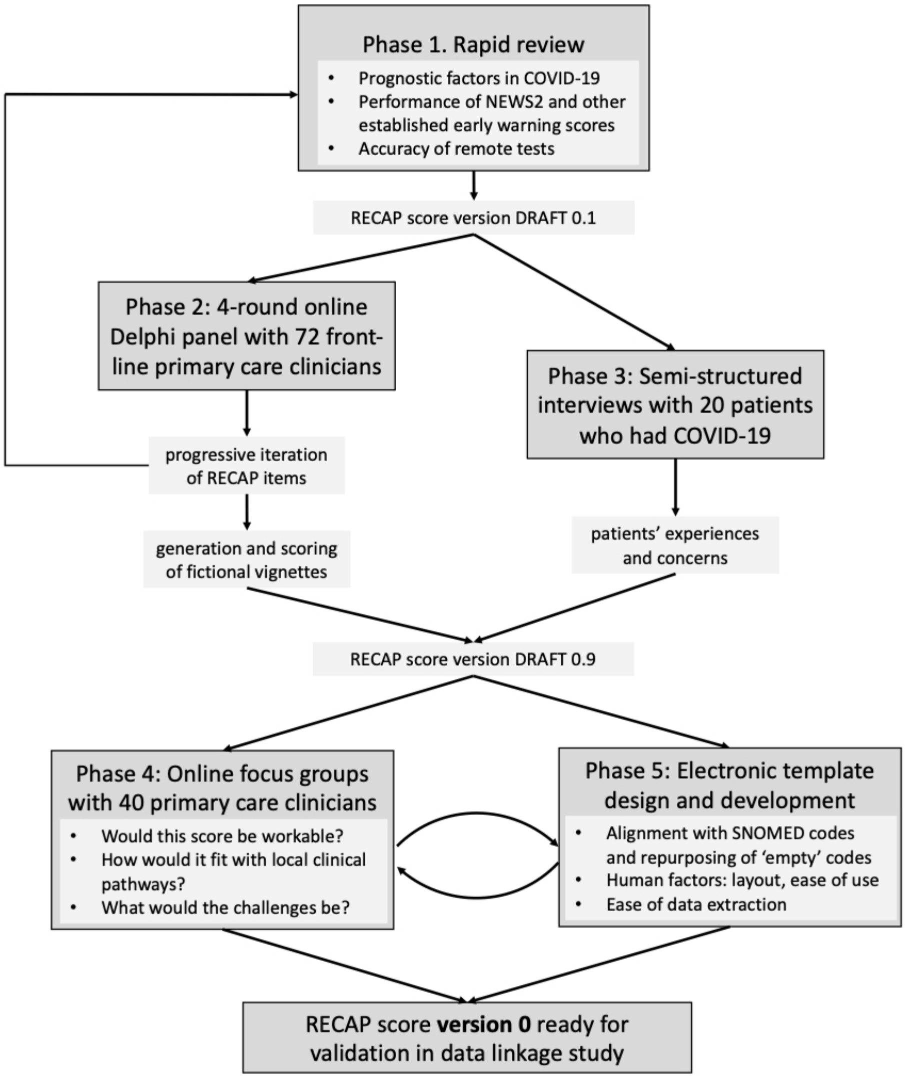

Figure 1: Study flowchart

Figure 2: Summary of RECAP-V0 items

RED ALERT CRITERIA: If patients have any of the following, consider 999

Adapted from national primary care covid-19 recommendations

Shock or peripheral shutdown

- Reduced level of consciousness
- Extremities – cold and clammy to touch
- Pallor – skin is mottled, ashen, blue or very pale
- Reduced urine output – little or no urine in last 24 hours

Severe breathlessness

- Rapid, significant deterioration in breathing in last hour
- New breathlessness at rest
- Newly unable to complete sentences
- Sudden onset of breathlessness

Other red flags which may be non-covid-19 related e.g.

- Severe central chest pain
- Collapse

RECAP-V0 ITEMS FOR PATIENTS WHO DO NOT HAVE RED ALERT SYMPTOMS OR SIGNS

		0 points	1 point	2 points	3 points => refer urgently	Calculate
1	Heart rate (per minute)<br>*(if heart rate not available, score 1)*	51-90	41-50 or 91-110 or missing data	111-130	≤ 40 OR > 130, if unexplained	
2a	Shortness of breath	Not breathless at all	Breathless on moderate exertion e.g. walking room to room	Breathless on mild exertion e.g. getting out of a chair	Severe breathing difficulty; can't complete sentences at rest	Highest of 2a or 2b
2b	or Respiratory rate (per minute)	12-20	21-24	9-11 or 25-29	8 or less, or 30 or more	
3	Trajectory of breathlessness	Same or better than yesterday	Breathless, worse than yesterday	-	Significant deterioration in last hour	
4a	Oxygen saturation at rest	96% or above	95% (don't do 40-step test unsupervised)	94% (don't do 40-step test unsupervised)	93% or below (don't do 40-step test)	Highest of 4a, 4b & 4c
4b	or Saturation after 40 steps	Fall of 0-1%	-	Fall of 2%	Fall of 3% or more	
4c	or Profound tiredness or fatigue	None or mild	Noticeably more tired doing usual activities	Struggling to get out of bed	Unable to speak because of tiredness	
5a	Temperature	≤ 38 °C	38.1-39 °C	> 39 °C or < 35 °C	-	Highest of 5a or 5b
5b	or Feeling feverish with shivers	None	Feverish or chills	Uncontrollable shivering	-	
6	Time from first symptom (days)	7 or fewer	8 or more	-		
7	Muscle aches	None or mild	Moderate	Severe		
8	Cognitive decline	No	Less mentally alert than usual	New and worsening confusion	Reduced level of consciousness	
9	On COVID-19 shielded list (or has been inadvertently left off it)?	No	Yes	-	-	
10	Other risk factors for poor outcome? e.g. age, ethnicity	0-2	3 or more	-	-	
TOTAL (simulated score)						

CLINICIANS' INTERPRETATION OF RECAP-V0 SIMULATED SCORE		
Number of points	Provisional interpretation	Provisional recommendation
7 or more total or 3 on any item or extremely high level of clinical concern	HIGH RISK	Consider urgent referral
4-6 or more total or high level of clinical concern	MODERATE RISK	See in hot hub or virtual ward, or arrange home visit
0-3 total	LOW RISK	Advice and monitor at home

Scores and calculation suggested by Delphi panel and used for simulation.
See A4 on bmj.com for full wording of items including cautions for specific groups.

For peer review only - http://bmjopen.bmj.com/site/about/guidelines.xhtml

Additional File 1: Focus group prompt questions

1. How has your **experience managing COVID-19** been? **How do you make decisions about who to refer on**?
2. Do you **use any clinical decision score**? If so, which one?
3. What would a warning score **need to do for you**?
4. **Which patients does it need to select** for you?
5. How was your overall **experience using RECAP**?
6. Which were the main **positive aspects of RECAP**?
7. Did you have **any difficulties using RECAP**? Which?
    a. In particular, how does clinical uncertainty (i.e. difficulties assessing objective measures, such as HR) impact the application of RECAP?
    b. How do remote communication barriers (i.e. language, no video, poor phone line) impact the application of RECAP?
    c. Was time a problem? (i.e. Is it short enough to be useful in clinical practice?)
8. **How do you think these problems/difficulties could be eliminated or overcome**? Can you anticipate any possible solutions?
9. How do your **local and structural arrangements** influence your ability to apply RECAP?
10. Do you feel you need any additional information or training on how to use RECAP?
11. Is there anything else you want to add or feel we should have spoken about?
12. Demonstration of RECAP template using screen share.

***

Other possible questions to be discussed:

Are there any other ways to achieve #3?

Does RECAP seem capable to select those who would, or not benefit from oxygen therapy or heparin?

For peer review only

Additional File 2: Qualitative comments during Delphi process

Pulse rate

"We have noted that about 50% of our patients have BP monitors at home, which has been very helpful in our video consultations. In addition, we send out sats bus out to those patients we are concerned about, so have been able to include the PR [pulse rate] even in patients without." [round 1]

"I think it [pulse rate] is really helpful in identifying sick young fit people who are otherwise compensating quite well and may have normal resting sats and a slightly high RR [respiratory rate] and significant fatigue and look ok. Less helpful in older adults unless very high. I have no idea of its predictive value for outcomes though." [round 1]

"We have seen a lot of tachycardia in patients with milder symptoms so I think 2 rather than 3 is an appropriate score" [round 2]

"A bradycardia below 40 is an emergency whether or not a patient is on a B-blocker so the advice to adjust by 10 should not apply. Just because a patient uses a B blocker doesn't mean they can tolerate a low cardiac output when septic/unwell." [round 3]

"They [pulse rate cut-offs] don't align with London guidance"

Temperature and symptoms of fever (chills, shivers)

"In my (pre-Covid) experience, patients are excellent at recognising rigors as being different from a normal high temperature." [round 1]

"Temp 37.8 by NHS 111 is I think more about meeting clinical criteria for isolation, rather than because of any clinical utility. I think your cut offs are sensible." [round 2]

"'use peak temperature before paracetamol' implies a level of control that the clinician won't have. Why not 'highest recorded temperature in the last 24 hours' or similar?" [round 3]

"I would just score 1 for a temperature, rather than the height of the temperature" [round 3]

"If you mean 'rigors' then the orange box should have the word 'rigors' in it. Shivers and chills aren't rigors (I've had both). 'Feverish or chills with uncontrollable shivering' perhaps is closer to the implied meaning." [round 3]

"I suspect that chills is a greater reflection of malaise and temperature than the height of pyrexia and therefore reflects greater illness (but I'm guessing!)." [round 3]

Respiratory rate

"We have found RR [respiratory rate] as a very helpful tool with our video consultations and are using it to determine which patients require home visit/sats bus/hub attendance, with the cut off of more than 24 and less than 9. We are requiring RR for all referrals to our hot hub." [round 1]

"Below 9 to score two- no way! That's a 999 emergency. I have MRCEM and was an intensivist for several years. A low resp rate scares me a lot more than a high one. A low RR tells you the patient is peri-arrest. A high RR is worrying but tells you that the patient is - for the moment - responding appropriately to their respiratory or metabolic problem. A low RR tells you that they are no longer able to do so and are about to die without

For peer review only

intervention (some exceptions, of course, e.g. BZD plus alcohol OD when people often maintain a scarily low RR without apparent significant compromise - but not relevant here)." [round 2]

"I would amend to 2 = 25-29 and score 3 if greater than or equal to 30, to align with CURB and other scores. A RR of 30 is pretty rare and severe." [round 2]

"Almost certainly a good sign but worried about its feasibility" [round 3]

Breathlessness and silent hypoxia

"We have seen many patients with silent hypoxia. These patients have tended to be extremely fatigued. Would you consider including a normal RR but with extreme fatigue as a score 3?" [round 1]

"In practice we have noticed that patients are not articulating their breathlessness well - asking them about how their symptoms (both breathlessness and fatigue) are affecting their usual activities has been key - struggling to get out of bed, for example, is often a very significant change, for others it's not being able to manage the hoovering, for all it's changes to their usual activities and routine" [round 1]

"Would be good to have something to try and capture the silent hypoxics - such as feeling more fatigued and exhausted on exertion? Which I have had patients describe? Or a feeling that breathing is laboured but not breathless. Like at altitude." [round 1]

"Persistent, progressive SOB is a red flag" [round 1]

"Listening to friends (sadly several) who have suffered with COVID and even been to ICU, lot of them were that tired that they could not even speak." [round 2]

"Maybe 2 and 3 are too close together? How about 0: as it is 1: new breathless on moderate exertion eg up stairs 2: Breathless on mild exertion eg walking across room. 3: unable to complete sentences or severe difficultly breathing" [round 2]

"Struggling to get out of bed might not be that unusual for some people!" [round 2]

Illness trajectory

"The patients I've been worried about tend to be pyrexial >38 in week 2" [round 1]

"From the various narratives on the illness I wonder about days since symptoms began - it seems that patients in the first 7 days of symptoms may be less likely to deteriorate, whilst those whose symptoms go on after this probably need assessment. Careful assessment of confusion feels important - for my phone calls I am spending time talking to patients to assess for any level of confusion. This for me is a 'sign of hypoxia' - but that is not flagged clearly enough in the above for my liking." [round 1]

"[Tiredness] varies so much on stage of illness. Those with mild symptoms all c/o feeling very tired in the first 5 days, often not able to get out of bed for a day or two. But can manage their ADL [activities of daily living]. Worsening fatigue around day 7-10, having initially been feeling better, is a much worse prognostic feature in my limited experience." [round 1]

Pulse oximetry and exertional desaturation

For peer review only

"I think the walk test is really important." [round 1]

"Validated scoring system already in place I ask patients to walk 40 steps and then check by video with hand in their chest easier to see and count and sees how they are on exertion in comparison to rest ." [round 1]

"Until we know more I'm not sure that we can leave patients with pre-existing normal lungs at home with a score of 93%- this may be the best early warning that we can find." [round 2]

"Always a problem what to do with chronic lung disease. 6% [correction for chronic lung disease] is quite steep (since obviously depends on degree of chronic hypoxia) but I don't have any more rational suggestion." [round 2]

"NB: pan-London respiratory guidance decided not to specify a number for desaturations in latest draft. I had to miss that meeting so not sure about the reasoning, but I think it was something along the lines of needing to use clinical judgement. Personally, I think 'clinical judgement' needs some guidance as this is a new disease entity, so strongly support having specific numbers on this to guide clinicians." [round 2]

Muscle aches

"This is interesting. I just have not spoken to or seen many people who complain of severe muscle pains at all. This might be because I am seeing people (in the community as a GP) who have mild to moderate disease, and those with really severe disease with severe myalgia bypass their GPs and go straight to hospital. If hospital colleagues are saying it does seem to predict severity then leave it in." [round 4]

"muscle pain has been very common in our unwell patients" [round 4]

Red flag signs

"Central cyanosis needs 999." [round 1; 30 similar comments]

Comorbidities and demographic risk factors

"3 for 61yo male with BMI of 32 is not equal to active cancer, immunosuppressed and high fever in week 2?" [round 2]

"If the other questions don't raise concerns then i don't want to send thousands of obese diabetic hypertensives with mild disease for assessment - I think they need to be given specific advice that they are at risk of becoming more unwell quickly - so please call back if any concerns" [round 2]

"I think the cut offs are perfectly rational, but the comorbidities are complicated and hypertension in particular." [round 2]

"All patients on shielded list and having 3 risk factors will score 4 meeting the criteria for F2F assessment even if they have no concerning clinical features. This means that any non-white male patient over 65 and on the shielding list needs a F2F assessment before we even start with the clinical assessment, which is not appropriate. So I think the scoring levels need to be reconsidered and reduced for this or 6b. My inclination would be to reduce this to a 1 if on list and leave 6b as it is." [round 3]

"I would have the top score as '4 or more'. A fit 68yr old Asian male with no other illnesses, or a white male with either well controlled hypertension or diabetes scoring 2 feels a little high." [round 3]

For peer review only

Other comments

"I don't trust China data" [round 1]

"I think you have covered the important things. The key here is to assess physiology. I think history is less important. The patients who become very unwell have a large systemic inflammatory response or severe pneumonia/pneumonitis so we need to focus on identify when people have SIRS or significant respiratory compromise." [round 1]

"I am very concerned about the application of clinical scores to patients that don't capture the individuality of patient circumstances - particularly if they are to be used to inform important decisions such as admission to hospital or deciding that a patient is in need of end of life care. There is always a danger that scores will be used to reduce and resolve these hugely complex issues - and are very clinician dependent." [round 1]

"I am not clear on the aim of the scoring system, and whether it acts as 'protocol' rather than a decision augmenting tool.. I hope you don't mind my honesty as I would like to help produce a simple, user-friendly diagnostic and prognostic tool.. but I think it needs to augment existing high level clinical skills rather than be another protocol to fill, creating over-reliance on this tool and cognitive distraction in the field. " [round 2]

"a really important part of our assessment in primary care is social circumstance and / or ability to mobilise community services. Lack of either of these can lead to admission too - while they may not be clinical "red flags" they may be worth including somehow as they contribute to the rationale for the decision to admit to hospital (and capture a little about the person being admitted)" [round 2]

"An observation. I realise that it is an unavoidable consequence of remote assessment but if you have a device to measure sats or pulse rate, you have 'more chance' of having a higher overall score." [round 3]

"There are too many questions if all included, so it is too easy to get a high score - need to reduce the number - could aim for 8 questions as in NEWS2? Would it be worth considering a table top exercise before sending out for initial testing? (eg give some scenarios & decide if admit or not & then do score to see if confirms decision or not?)." [round 3]

For peer review only

Additional File 3: Summary of vignette study

We used five vignettes, deliberately presented as ‘grey cases’ with considerable clinical, technical and social uncertainty (missing data, vague symptoms, potentially irrelevant material). In the first round of vignettes, we asked participants to score each item on the RECAP instrument, calculate the RECAP score and then say what they would recommend for the patient and whether the score reflected their clinical concern.

The 51 responses to this round showed surprising variation (e.g. the RECAP score varied from 2 to 16 in one vignette). However, free text comments suggested that the respondents were unsure how to calculate the score from the individual items (uncertainty which to add with ‘and’ and which to use as alternates with ‘or’, and whether to count the clinical concern within the RECAP score or separately from it).

We therefore ran a second round of vignettes (using three of the five), and using an automated scoring system. This produced 37 responses and a more consistent response, though still a fairly wide spread. Free text responses suggested that respondents found the vignettes time-consuming and somewhat confusing.

Details analysis is below.

VIGNETTE 1: Mrs Rahman

> *Mrs Rahman (South Asian ethnicity) is 78; she lives with her extended family who are concerned about her. She has had a dry cough and has felt hot for 6 days. Her appetite is poor (she has apparently lost her sense of taste). She spends most of her time in bed and shows no signs of getting better. When you try speak to her, you have no common language but her daughter-in-law says she is not short of breath, just very tired. She says her mother-in-law gets up to go to the toilet, and comes downstairs once a day to watch a TV programme. She says she doesn’t look blue or pale. She’s been hot and sweaty but has not had uncontrollable shivering, but she is aching quite badly in her back and down her legs, and is very worried. Mrs Rahman has well-controlled type 2 diabetes (on metformin). Her BMI is 27 and a recent BP was 130/85. She had stage 2 breast cancer 20 years ago but no recurrence. The family do not have a thermometer, blood pressure machine, oximeter or other equipment, and nobody has access to a video application.*

Scoring in round 1 (51 responses):

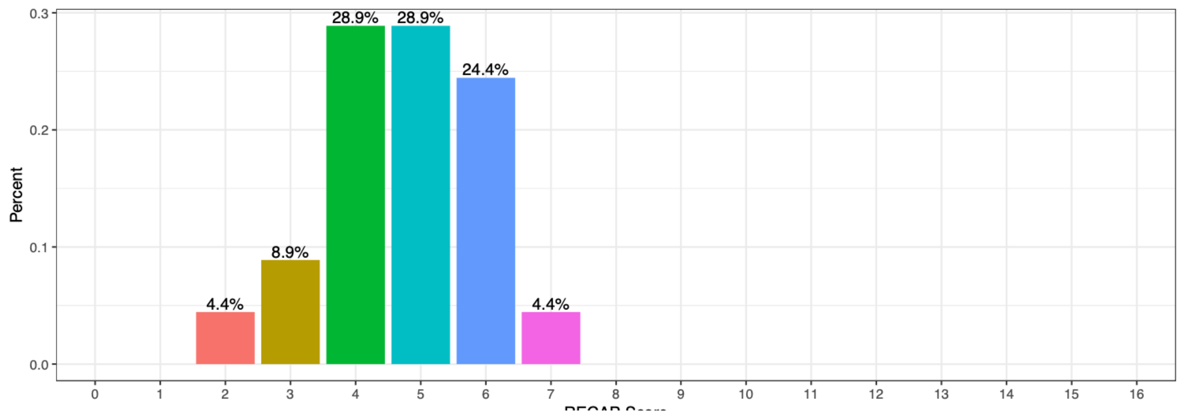

For peer review only - http://bmjopen.bmj.com/site/about/guidelines.xhtml

Scoring in round 2 (37 responses)

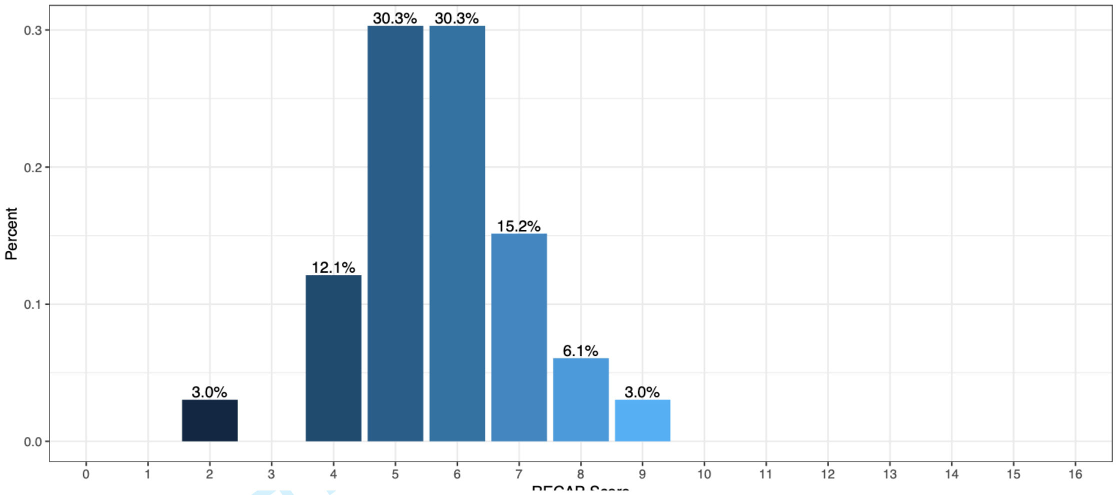

In sum, 82% of the first sample and 72% of the second sample would see in hot hub. 13% of first sample and 3% of second sample would reassure. Interestingly, 4% of the first sample and 24% of second sample would refer to hospital.

Responses to individual items showed that the main ones accounting for variation were

- Tiredness: Noticeably more tired doing usual activities (70%) versus struggling to get out of bed (30%)
- Fever: None (10%), feverish or chills (87%), feverish or chills with uncontrolled shivering (3%)
- Duration of temperature: not applicable (9%), fewer than 7 days (91%)
- Muscle aches: none (3%), moderate (61%), severe (36%)
- Is patient on shielded list? Yes 6%, no 94%.
- Risk factors for poor outcome? No 9%, yes 91%.
- Clinical concern: low 3%, moderate 70%, high 27%.
- Did RECAP reflect clinical concern? Yes 90% No 10%
- What would you do? Reassure 3%, see in hot hub 91%, refer urgently 6%

Qualitative comments:

Many said can't remember what the score is.

"It effectively has a bunch of 'not assessed' elements though, so the risk is that they cannot contribute to the score because missing, but missing does not equal 'not present' if there is a communication barrier (language but also no video or a poor phone line, or an inarticulate person who struggles to explain what they feel etc). I think it delivers the 'right' outcome though in the sense of face to face community review."

"Not clear how to score if no HR available Q4b) states feverish with shivers but then the score says "feverish or chills" scores 1 point... Q6b) not clear if I would have counted controlled hypertension as one factor, and also if I would use the BP cut off for diabetes in giving a point for diastolic >80... Scored 6 and then gave additional points for moderate worry = 8, which would result in urgent referral but I think I would have seen this patient in a hot site rather than admitting."

"Seeing her in the Red COVID Assessment Unit would feel right. Then we could assess parameters and see what her obs are."

Conclusion: The early warning score would prompt almost all GPs to assess this patient further. This seems appropriate given the multiple risk factors, missing data and language barrier.

For peer review only - http://bmjopen.bmj.com/site/about/guidelines.xhtml

VIGNETTE 2: Mr Jamieson

Mr Jamieson (African-Caribbean ethnicity) calls you urgently; he is 57 and lives with his wife. He has been unwell for 9 days, but only started feeling really bad yesterday. He has absolutely no energy and finds it hard to breathe, but is wondering if that's due to his asthma (for which he normally takes a Ventolin inhaler). He isn't out of breath at rest but was very short of breath brushing his teeth this evening, whereas this morning he could do that without a problem. He doesn't sound out of breath on the phone to you, and is able to complete sentences; an attempt at making a video connection fails. He says his peak flow rate is 425 (predicted for age 510; last asthma check 450). He forced himself to get up this morning, and has spent all day in a chair, finding it a struggle even to get to the toilet. He has a BP machine because he's on medication (amlodipine) for high blood pressure. Using his machine, his blood pressure today is 120/70 and pulse 105 (recent clinic values were 148/90 and 78). He's taken his temperature with a mouth thermometer he found in the cupboard but it only reads 35 even though he's sweated so much his wife had to change the sheets. When asked about feeling hot with shivering and chills, he says yes he's had all those, quite badly on one occasion. His muscles don't ache. He has sickle cell trait, but was told this would never cause him any problems. His BMI is 33, and a recent check-up showed an eGFR of 80.

Scoring in round 1 (51 responses):

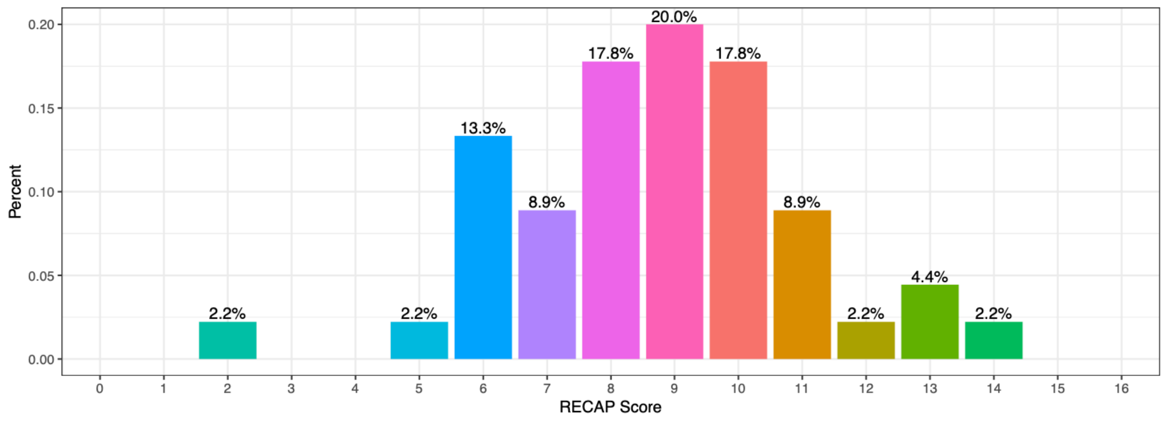

Scoring in round 2 (37 responses):

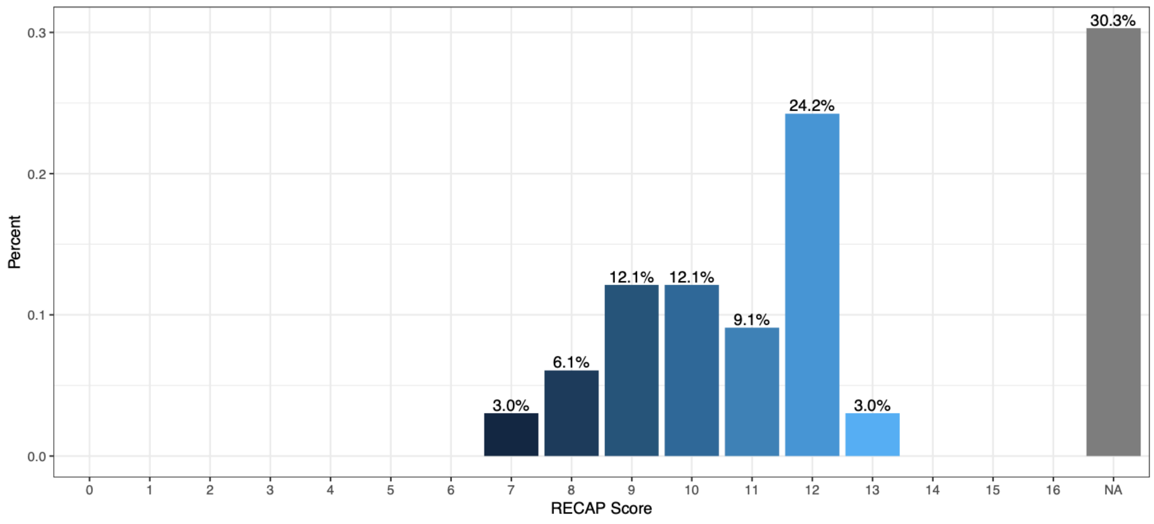

(the N/A column is because the patient has been referred urgently without completing the RECAP score)

For peer review only - http://bmjopen.bmj.com/site/about/guidelines.xhtml

In sum, 82% of first sample and 100% of second sample would refer this patient urgently.

Responses to individual items showed that the main discrepancies were

- Tiredness: Noticeably more tired doing usual activities (10%) versus struggling to get out of bed (90%)
- Shielded list yes 77% no 23%
- Duration of temp not applicable 42%, 8 or more days 45%
- Other risk factors for poor outcome 0-2 37%, 3 or more 63%
- Whether to refer urgently without completing RECAP or continue with RECAP
- Level of clinical concern: high 66% extremely high 31%
- Did RECAP capture your level of concern? Yes 77%, no 23% r1 and 90%, 10% r2.

Qualitative comments

Some wanted to ask him more questions about his asthma.

"Rapid deterioration in symptoms over 12 hours - high number of risks. Is at that 9-10 day tipping point for COVID - I would be admitting urgently."

"composite assessment - day 9, acute deterioration SOB, underlying asthma, risk factors. degree of SOB and deterioration is overriding concern"

"I think the profound fatigue is very concerning - I don't know that we are certain whether this is always a sign of silent hypoxia or whether it may just be due to immune response to SARS COV-2. I think I would want to check oxygen saturations, and most likely admit."

"Day 9, SOB brushing his teeth! Likely heading towards ARDS. Wouldn't want to waste time with Hub clinic with this one but would call him an ambulance."

"Although there is an argument (maybe) for see urgently in a hub - if his O2 sats and work of breathing are normal, what is hospital going to do? Xray? Rate of deterioration and duration of illness I think are the critical factors here that tip towards hospital assessment."

[The last comment above illustrates, I think, a GP who is less aware of the possibility of ARDS than the penultimate commenter above – ie RECAP score is budging this GP towards taking more aggressive action than they would otherwise have done – this is good!]

Conclusion: In every case in round 2 (where we calculated the score automatically), this high-risk patient would be referred urgently. The lower scores on round 1 may have been arithmetical or interpretation errors. Even in round 2, 31% of responders only had "moderate" or "high" clinical concern but would have been prompted to refer urgently.

VIGENTTE 3: Mrs Finlay

> *Mrs Finlay (White Irish ethnicity) is 88 and in a care home. She has multimorbidity (ischaemic heart disease, osteoarthritis, chronic pancreatitis and hypothyroidism) and on multiple medications, but her quality of life is good and she is normally mentally sharp. A recent conversation about 'ceiling of care' is documented in her medical record: she would like to be referred to hospital if appropriate. Her carers are concerned because after three days of a low-grade fever (37.5 on tympanic thermometer) and worsening breathlessness (which, since*

For peer review only - http://bmjopen.bmj.com/site/about/guidelines.xhtml

this morning, is present even when lying in bed), she has become delirious. Her blood pressure is 110/65 and pulse 120.

Scoring in round 1 (51 responses)

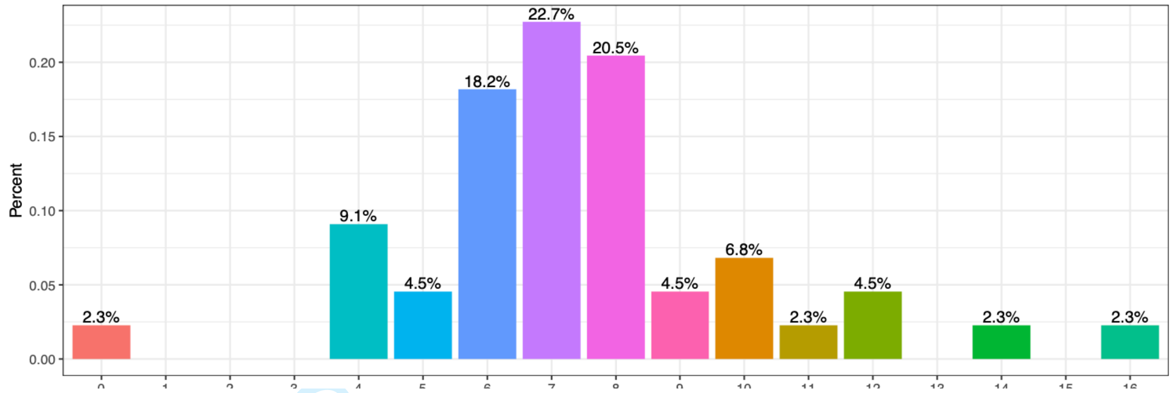

Responses to individual items showed that the main discrepancies were

- Clinical concern: moderate 7%, high 27%, extremely high 66%.
- Did RECAP reflect clinical concern? Yes 64% No 36%
- What would you do? See in hot hub 16%, refer urgently 82%

(Not included in round 2).
Qualitative comments:

"In practice, I would want a more detailed discussion with the carers about whether referral to hospital was "appropriate". Sounds like it should be, but hard to capture the nuance in a vignette. Unlike the one before or the one after, she is a hospital referral/999 as no point going to a community service if she's suitable for admission."

"Actually what I would do if at all possible is eyeball her (by video or in person) to establish if she is really delirious and very SOB. However, if I have to choose from only the 3 options above, I would have to admit. (Note: I am not sure whether you are using 'virtual ward' to include video consultation - this is not how we use it locally)."

"Given the delirium, and ceiling of care wishes, she needs an urgent work up."

"Delirium very difficult to score Also lots of causes of this in elderly eg UTI"

"Trajectory is important here, as is the new delirium and breathlessness at rest. Admit."

"RECAP itself didn't [capture my concern] (only 6, however does have 3 in one area)- however she has a red flag symptom - interestingly because of temperature/ no description of duration-/muscle aches etc, (really a lack of information as May happen in care home residents), the score is lower, but the presence of the red flag (confusion and new sobar) means that the score is less important - so overall the tool Accurately captured my concern."
[this DID capture the concern because RECAP says score 3 in one item → refer urgently]

Conclusion: This was the vignette with the most variation, due to two things: a) some GPs didn't see delirium as a red flag on the front page; b) some felt the delirium wasn't an indication to refer urgently (eg may have been a UTI). But more than 80% of GPs would refer given this story and score.

For peer review only - http://bmjopen.bmj.com/site/about/guidelines.xhtml

VIGNETTE 4: Mr Hughes

Mr Hughes is 72 (white British ethnicity) and lives alone. He has been unwell with Covid symptoms for 11 days. He was seen last week in the hot hub clinic because of breathlessness, and supplied with a home oximeter. Today, he feels more breathless than yesterday but says he can still walk around the living room. He's been measuring his saturations daily and the last three days have been 97%, 96%, 96%, but he feels much more tired today than he did yesterday. The oximeter measures his pulse at 98. Using a video app, you measure his respiratory rate at 24. You ask him to take 40 steps around his flat and re-measure his saturation levels. He does this, and says the level is still 96%, but a minute later it has fallen to 94%. His temperature with a mouth thermometer is 37.6 and he hasn't had shivers or chills. He had prostate cancer 10 years ago and was treated for depression last year after his wife died. His BMI is 28.

Scoring in round 1 (51 responses)

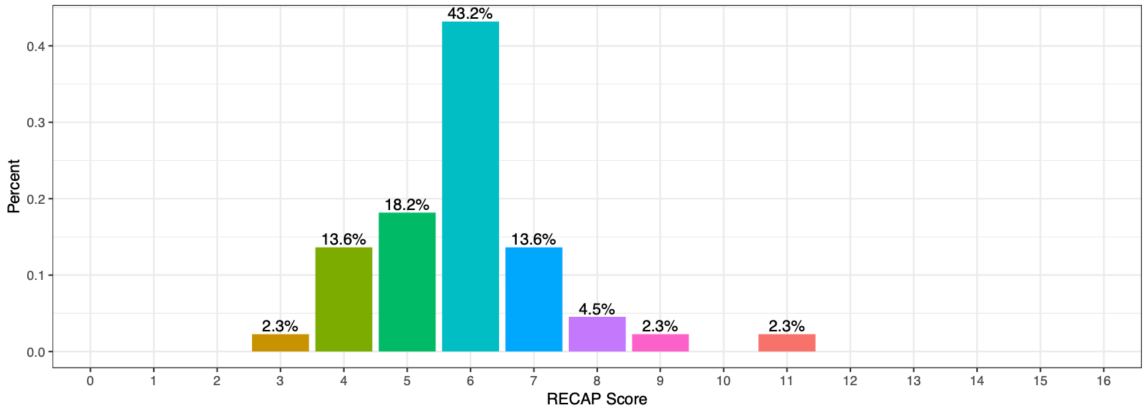

(no round 2 with this case)

In sum, 22% of this sample would refer this patient urgently; all but one responder would review in hot hub or at home.

Responses to individual items showed that the main discrepancies were
- Duration of temp: not applicable 42%, 8 or more days 45%
- Risk factors for poor outcome: 0-2 36%, 3 or more 64%
- Clinical concern: low 7% moderate 52% high 36% extremely high 5
- Did RECAP reflect clinical concern? Yes 80% No 20%
- What would you do? Reassure and advise 16%, See in hot hub 50%, refer urgently 32%

In sum, there was quite a bit of variation in what GPs subjectively felt was needed, but in all but one case, the patient would have had a hot hub review or hospital referral if score was followed.

Qualitative comments:

"Moderate risk - think the desaturations on walking are main red flag here"

"The post exertional hypoxia is a concern, and the isolation but there are psychosocial factors that might contribute to his malaise. He is not so ill that he needs hospital and a prompt Hub appointment could suffice."

"Deteriorating despite home management, desaturating, and vulnerable living alone. I'm not sure here what the hot hub would add to his assessment. I think he needs ED review."

For peer review only - http://bmjopen.bmj.com/site/about/guidelines.xhtml

"He already has a pulse oximeter. He will need daily calls but is probably safe to be managed remotely today."

Conclusion: The spread here was because some GPs did not interpret exertional desaturation as a serious 'red flag' sign. The RECAP score pushed those GPs to reviewing the patient urgently or sending him to hospital. This is what the score is designed to do.

VIGNETTE 5: Mr Liu

Mr Liu is a 37-year old postgraduate student from Taiwan who lives in a shared house of multiple occupation. He's worried because he has been unwell for 10 days and has a cough and a high fever (measured at 39 with a mouth thermometer). He's also finding it hard to catch his breath – something that's been going on for three or four days. He doesn't think it's worse today than yesterday. He doesn't know his housemates well, but he knows two of them have been unwell. He is not registered with your practice so you have no previous records on him, but he tells you he might have been diagnosed with diabetes back in Taiwan, he's not sure. His father had it and he is a bit overweight, though he doesn't know his actual height and weight. He's been lent an oximeter by one of his housemates and taken a reading – his pulse is 115 and saturation 95%. On a video call, you think he looks pale and anxious, and his respiratory rate is 22.

Scoring in round 1 (51 responses)

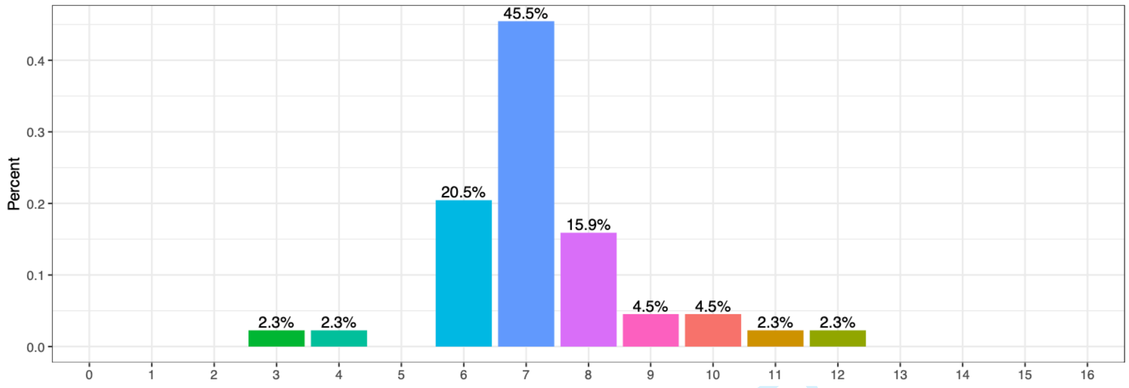

Scoring in round 2 (37 responses)

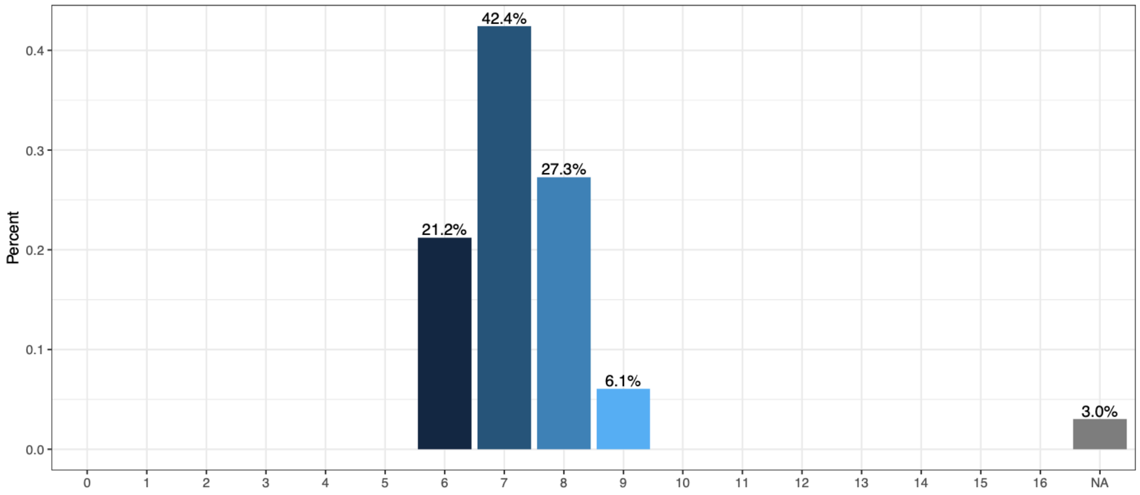

- Level of clinical concern r1 low 2%, moderate 52%, high 39%, extremely high 7%; r2 moderate 3%, high 66%, extremely high 31%.
- What would you do? R1 reassure and advise 5%, see in hot hub 65%, refer urgently 27%; R2 Reassure and advise 18%, See in hot hub 48%, refer urgently 33%
- Did score capture level of clinical concern r1 yes 80% no 20%; r2 90%, 10%.

In sum, 75% of r1 and 80% of r2 would have referred this man urgently.

Qualitative comments

"I think it came up higher than I would have expected."

"score suggests consider urgent referral - well I've considered and am happy for him to be seen in hub. score would be higher still if he has DM and BMI >35 - which Ive not scored - but why he needs to be seen rather than have phone advice."

"I think there's a degree of uncertainty around this case. We don't know the patient and there is some uncertainty regarding his PMH, but the safest thing is to assume he does have diabetes. It can be quite hard to differentiate between anxiety and an unwell patient over video especially, and paleness and tachycardia makes me concerned regarding shock. I would be admitting him."

"During vignette, I was wondering would I really want to see this patient in a hot hub, to be knower, with Sats of 95 in a fit and well young man; that is worrying enough to me So yes I think overall though not so barn door, I feel direct referral to hospital is appropriate for him mainly given his sats/ duration etc"

Conclusion: This vague case had a number of potentially worrying features and inevitably they were interpreted slightly differently by the responders, but in every case the patient would have either been referred straight to hospital or seen in a hot hub. Once again, the score seems to be doing its job of prompting the GP to act on a sign or symptom they may not be aware is associated with poor prognosis.

ADDITIONAL COMMENTS

Complaints about layout and not seeing score in front of them.

Complaints about time it took.

--

TG, PT
1.6.20

For peer review only - http://bmjopen.bmj.com/site/about/guidelines.xhtml

Appendix A4: Full version of the RECAP-V0 score

REMOTE COVID-19 ASSESSMENT FOR PRIMARY CARE

This is the simulated early warning score used in the Delphi exercise in the qualitative (item development) phase of the RECAP study. The score is designed to support assessment of, and communication about, COVID-19 patients at the primary-secondary care interface. It is not intended to replace clinical judgement. It has been produced by professional consensus but has not yet been tested against clinical outcomes or formally validated.

If the RECAP score is high, it will not tell you whether to refer for active management (e.g. hospital or hot hub), or arrange palliative care (other factors will of course influence this decision).

RED ALERT CRITERIA: If patients have any of the following, consider 999

Adapted from national primary care covid-19 recommendations

Shock or peripheral shutdown
- New confusion (including delirium)
- Reduced level of consciousness
- Extremities – cold and clammy to touch
- Pallor – skin is mottled, ashen, blue or very pale
- Reduced urine output – little or no urine in last 24 hours

Severe breathlessness
- Rapid, significant deterioration in breathing in last hour
- New breathlessness at rest
- Newly unable to complete sentences
- Sudden onset of breathlessness

Other red flags which may be non-covid-19 related e.g.
- Severe central chest pain
- Collapse

RECAP-V0 SCORE FOR PATIENTS WHO DO NOT HAVE RED ALERT SYMPTOMS OR SIGNS

		Score 0	Score 1	Score 2	Score 3 => refer urgently	Score
1	Heart rate (per minute) *(if heart rate not available, score 1)*	51-90	41-50 or 91-110 or missing data	111-130	≤ 40 OR > 130, if unexplained	

Use medically approved device if available, or patient's own. Lower threshold for tachycardia by 10 bpm if beta-blocker or other heart-slowing drug taken in past 24h, but use standard thresholds for bradycardia. Adjust score if known to have physiological bradycardia (e.g. athlete).

2a	Shortness of breath	Not breathless at all	Breathless on moderate exertion e.g. walking room to room	Breathless on mild exertion e.g. getting out of a chair	Severe breathing difficulty; can't complete sentences at rest	Highest of 2a or 2b
2b	or Respiratory rate (per minute)	12-20	21-24	9-11 or 25-29	8 or less, or 30 or more	

Score any breathlessness that patient or carer is concerned about. Take account of pre-existing conditions such as COPD. Assess respiratory rate by video, ask patient to place hand on chest. An anxious patient may be hyperventilating.

3	Trajectory of breathlessness	Same or better than yesterday	Breathless, worse than yesterday	-	Significant deterioration in last hour	

Pay careful attention to a history of recent worsening of breathlessness, especially if this is what concerns patient or carer.

4a	Oxygen saturation at rest	96% or above	95% (don't do 40-step test unsupervised)	94% (don't do 40-step test unsupervised)	93% or below (don't do 40-step test)	Highest of 4a, 4b & 4c
4b	or Saturation after 40 steps	Fall of 0-1%	-	Fall of 2%	Fall of 3% or more	
4c	or Profound tiredness or fatigue	None or mild	Noticeably more tired doing usual activities	Struggling to get out of bed	Unable to speak because of tiredness	

Patient should have warm hands and place oximeter device correctly. Lower thresholds if patient has chronic lung disease with known hypoxia (typically by 6%, but will vary – use usual baseline readings to adjust if known). Do exertion test only if clinician in attendance or if saturation is 96% or higher at rest (lower threshold for chronic lung disease). Saturation levels may fall for 1 min after stopping exercise. Most patients

with covid-19 feel some fatigue, but profound fatigue may be a feature of 'silent hypoxia'. Take account of patient's baseline level of fatigue.

5a	Temperature	≤ 38 ºC	38.1-39 ºC	> 39 ºC or < 35 ºC	-	Highest of 5a or 5b
5b	or Feeling feverish with shivers	None	Feverish or chills	Uncontrollable shivering	-	

A tympanic thermometer is preferred. Use highest recorded level in last 24 hours. A low reading may reflect user error.
If the patient has no reliable thermometer (or in addition to the temperature reading), explore for a description consistent with rigors.

6	Time from first symptom (days)	7 or fewer	8 or more	-		

Number of days since onset of first symptom

7	Muscle aches	None or mild	Moderate	Severe		

8	Cognitive decline	No	Less mentally alert than usual	New and worsening confusion	Reduced level of consciousness	

9	On COVID-19 shielded list (or has been inadvertently left off it)?	No	Yes	-	-	

Includes: • organ transplant • current chemotherapy or immunotherapy • severe lung condition such as cystic fibrosis • sickle cell anaemia • high dose steroids or other immunosuppressants • blood or bone marrow cancer • lung cancer on radiotherapy • splenectomy

10	Other risk factors for poor outcome in COVID-19?	0-2	3 or more	-	-	

For example: *• Age > 65 • BMI > 35 • male • non-White ethnicity • diabetes • hypertension • coronary heart disease • chronic kidney disease • vulnerable and isolated*

TOTAL	

Clinical concern component (be guided by clinical concern whatever the RECAP score; do not add this into the score but use it to over-ride the score if appropriate):

After assessing the patient, what is your level of clinical concern (regardless of RECAP score)?	Low	Moderate	High	Extremely high

RECAP score	Meaning	Recommended action
7 or more total or 3 on any item or extremely high level of clinical concern	HIGH RISK	Consider urgent referral
4-6 or more total or high level of clinical concern	MODERATE RISK	See in hot hub or virtual ward, or arrange home visit
0-3 total	LOW RISK	Advice and monitor at home

For peer review only
