# Supplementary material for: What items should be included in an early warning score for remote assessment of suspected COVID-19? qualitative and Delphi study
Source: BMJ Open. 2020 Nov 12;10(11):e042626. doi: 10.1136/bmjopen-2020-042626 (PMC7662139; doi:10.1136/bmjopen-2020-042626)
Supplement: Reviewer comments [file bmjopen-2020-042626.reviewer_comments.pdf]

PEER REVIEW HISTORY

BMJ Open publishes all reviews undertaken for accepted manuscripts. Reviewers are asked to complete a checklist review form (**http://bmjopen.bmj.com/site/about/resources/checklist.pdf**) and are provided with free text boxes to elaborate on their assessment. These free text comments are reproduced below.

ARTICLE DETAILS

**TITLE (PROVISIONAL)**	What items should be included in an early warning score for remote assessment of suspected Covid-19? Qualitative and Delphi study
**AUTHORS**	Greenhalgh, Trisha; Thompson, Paul; Weiringa, Sietse; Neves, Ana Luisa; Husain, Laiba; Dunlop, Merlin; Rushforth, Alexander; Nunan, David; de Lusignan, Simon; Delaney, Brendan

VERSION 1 – REVIEW

**REVIEWER**	Matthew Sperrin<br>University of Manchester, UK
**REVIEW RETURNED**	06-Aug-2020

**GENERAL COMMENTS**	This paper describes the construction of an early warning score for severe covid-19 for use in covid-19. The construction uses a mixed methods framework, and essentially develops, by consensus, an expert system, with input from both clinicians and patients. I was somewhat surprised that there was no clinical/outcome data used to support the development of the early warning score. I do agree that there are elements of developing such a score that are best assessed through focus groups, surveys etc, and indeed in the 'usual' clinical prediction modelling pipeline, these aspects do not receive the attention they deserve.<br><br>However, I cannot see, given the patient outcome data we have available to develop and validate a model, why we would instead rely exclusively on anecdote and clinical opinion, however systematically and robustly this has been collected. The paper needs to better justify this: why the more 'standard' pipeline, involving patient outcome data, was not followed here. There are, no doubt, good reasons, but these need to be made explicit.

**REVIEWER**	John Kellett<br>Hospital of South West Jutland<br>Denmark
**REVIEW RETURNED**	Founder and major shareholder of Tapa Healthcare DAC

**GENERAL COMMENTS**	This paper presents a most interesting concept, which I strongly support. It describes the development of a score to detect deterioration of patients with COVID 19. I appreciate that currently all research must appear to be relevant to COVID 19, but are any of the “red flags” and other items of the proposed score unique to COVID 19? Is COVID 19 different from any other illness? Their assertion that “Assessment of a patient with suspected COVID-19 in primary care is fraught with uncertainty, since it is a new disease whose clinical course does not mirror other pneumonias” seems a little dubious. Do we know that is true for sure?

	There has been a recent study in Uganda in this area that authors might find of interest and would support their proposal [ref]. All the variables identified have been long been recognized as associated with a poor outcome, but in different eras have been associated with different conditions. Recently some of them have been attributed to bacterial sepsis, and now COVID 19. The reality is the variables identified signal possible serious life-threatening illness that needs urgent attention. What the proposed study might determine, and which would be an important contribution to clinical medicine longer after this pandemic is over, is if changes in these variables may occur in advance of catastrophic vital sign changes and, hence, lead to earlier and more effective interventions.<br><br>The score is designed for use on patients with suspected COVID 19. The authors should make it clear if this is not supposed to be used as a diagnostic test. It is an assessment of clinical severity, which might be applied to COVID 19 or any(?) condition. However, the comment in line 24 page 34 of the appendix suggest others do not think this.<br><br>What does "suspected COVID 19" mean? A patient with a positive test, or waiting for a positive test, or just someone who someone thinks might have COVID 19? This is important because it is not clear what the response to the score should be, especially for moderate risk patients. It would be wiser to look on this score as a guide to what to do with ANY patient, and not just those suspected to have COVID 19. Most medical illness starts with the patient having nonspecific feelings of being unwell. The interval between these subjective nonspecific symptoms and the development of specific symptoms and objective signs may be seconds in acute cardiac disease, minutes in meningococcal sepsis, and hours or even days in other conditions. Surely the only thing unique about COVID 19 is that in around 15% of patients a mild illness turns into a serious illness after about 5 days. Therefore, a COVID 19 patient with mild to moderate symptoms might not be in danger for several days, whereas another condition might deteriorate far more rapidly. Therefore, the authors should consider in their Discussion exactly on what kind of patient the score should be performed on (my strong bias is any sick patient) and how often the score should be repeated, and in particular what to do with moderate risk patients (e.g. moderate risk patient repeat in 3 hours?). I am particularly concerned about these patients because they might be sick from something else. These issues should also be considered as part of the validation process.<br><br>Methodology: I am not expert on the techniques used, but they seem entirely reasonable to me. The study purpose was to develop clinical prediction models designed to identify COVID 19 patients who need escalation to next level of care. Was this made clear to all the participants, or did some think they were trying to develop a diagnostic model? (vide supra).<br><br>Minor issue<br><br>"…highly sensitive (detecting all patients who need onward referral) and fairly specific<br>(excluding all or most patients who do not)."<br><br>Have the authors gotten this the wrong way around? A specific test

	that is positive rules in a condition (SPPIN) and a sensitive test that is negative rules out a condition (SNNOUT).<br><br>Reference<br><br>Rice B, Leanza J, Mowafi H, Kamara NT, Mulogo EM, Bisanzo M, Nikam K, Kizza H, Newberry JA, Strehlow M, Global Emergency Care Investigator Group, Kohn M. Defining High-risk Emergency Chief Complaints: Data-driven Triage for Low- and Middle-income Countries. Acad Emerg Med. 2020 May 16. doi: 10.1111/acem.14013.

**REVIEWER**	Abayomi Salawu<br>Hull University Teaching Hospital NHS Trust<br>Hull York Medical School<br>United Kingdom
**REVIEW RETURNED**	17-Sep-2020

**GENERAL COMMENTS**	Typographical errors<br>Page 6, line 58: Advantages method. There is a preposition and a pronoun missing<br>Page 13 line 40: Solider

**REVIEWER**	Lim Wan Tin<br>Singhealth<br>Singapore
**REVIEW RETURNED**	28-Sep-2020

**GENERAL COMMENTS**	The patient interview group is a small population size. As almost 50% of the score is derived from patient described symptoms, the small population sample in the patient group is a potential limiting factor, leading to higher variability.and undercoverage bias. In this case by recruiting patient through social media, there is chance of voluntary response bias. Overall, the study methodology was robust. But the limitation of the study can be better discussed.

VERSION 1 – AUTHOR RESPONSE

Reviewer: 1
Reviewer Name: Matthew Sperrin
Institution and Country: University of Manchester, UK
Please state any competing interests or state ‘None declared’: None declared.

Please leave your comments for the authors below
This paper describes the construction of an early warning score for severe covid-19 for use in covid-19. The construction uses a mixed methods framework, and essentially develops, by consensus, an expert system, with input from both clinicians and patients. I was somewhat surprised that there was no clinical/outcome data used to support the development of the early warning score. I do agree that there are elements of developing such a score that are best assessed through focus groups, surveys etc, and indeed in the 'usual' clinical prediction modelling pipeline, these aspects do not receive the attention they deserve.

However, I cannot see, given the patient outcome data we have available to develop and validate a model, why we would instead rely exclusively on anecdote and clinical opinion, however

systematically and robustly this has been collected. The paper needs to better justify this: why the more 'standard' pipeline, involving patient outcome data, was not followed here. There are, no doubt, good reasons, but these need to be made explicit.

RESPONSE: We agree we needed to better justify the methodology. This has now been done (see pages 6). We have added the following sentence:

This in-depth qualitative design was chosen because of the novelty of the condition, the high degree of clinical uncertainty surrounding its acute management, and the added complexity of the need for remote assessment (which required judgements to be made without having fully examined the patient). For all these reasons, a detailed qualitative phase was considered essential before progressing to a standard validation exercise.

RESPONSE: It is important to note that COVID-19 is a new clinical condition of which clinicians were rapidly gaining experience. Unlike, for example, sepsis, for which there is clear consensus AND collection of the relevant clinical inputs, we had simply no idea what clinical data should be collected in order to follow the usual prediction modelling pipeline. This paper fills that gap. There is now, however, some limited published quantitative outcome data from hospitals around the world (though none from primary care). We capture some of that early data using rapid reviews. We've done some additional searches to cover the 13-week period that this paper was under review, and not found anything new to challenge the items in the score [action: David]. We did find the ISARIC-4C study of a hospital-based prediction score that was recently published in BMJ, in which 6 of the 8 items align with the RECAP items and the two non-aligned items were blood tests.

Incidentally, we question whether outcome data (which we're of course collecting in the quantitative phase currently ongoing) would have helped in this phase. The development phase of the study was focused primarily on input data. What symptoms were experienced by patients? What questions were asked by their clinicians – and what questions were not asked? How did GPs make decisions? What caused them concern? What were the touch points of the patient journey? How was the assessment of the patient constrained or shaped by the use of phone or video instead of face to face? These are open-ended questions best addressed through qualitative methods.

We fully agree that a quantitative phase with systematic collection of outcome data on thousands of patients is absolutely necessary. That is phase 2, and is now ongoing – see https://imperialbrc.nihr.ac.uk/research/covid-19/covid-19-ongoing-studies/recap/. But the detailed and meticulous qualitative phase to make sure we identified the right input variables to measure is, we believe, novel and original and we believe it needs to be published separately as it was a distinct phase in the study.

Reviewer: 2
Reviewer Name: John Kellett
Institution and Country: Hospital of South West Jutland, Denmark
Please state any competing interests or state 'None declared': Founder and major shareholder of Tapa Healthcare DAC

Please leave your comments for the authors below
This paper presents a most interesting concept, which I strongly support. It describes the development of a score to detect deterioration of patients with COVID 19. I appreciate that currently all research must appear to be relevant to COVID 19, but are any of the "red flags" and other items of the proposed score unique to COVID 19? Is COVID 19 different from any other illness? Their assertion that "Assessment of a patient with suspected COVID-19 in primary care is fraught with uncertainty, since it is a new disease whose clinical course does not mirror other pneumonias" seems

a little dubious. Do we know that is true for sure?

RESPONSE: Agree, important point. It's pretty incontrovertible of course that Covid-19 is a new disease whose clinical course does not mirror other pneumonias (see Box 1 in the paper for a discussion of novel symptoms/signs such as silent hypoxia). We've softened our claim to "differs from" rather than "does not mirror". But the reviewer raises a more specific question, which is whether the potential red flags we identified are unique to Covid-19 (a question we'll be able to answer confidently at the end of phase 2). There is a really important sub-question here, namely: what if the patient with suspected Covid-19 doesn't actually have Covid-19? So we could end up in a situation where the RECAP score is accurate if the patient has Covid-19 but not accurate if they're having (say) a bad asthma attack or going down with sepsis. However – and we acknowledge that at this stage we don't actually know – the red flags that came up in our qualitative work are almost identical to the red flags already being used for any deteriorating patient in primary care. The only one we added was "too breathless to speak" (and interestingly, "difficulty speaking" was one of the 12 high-risk chief complaints identified in the most interesting Ugandan study suggested by this reviewer). After phase 2 we'll be able to say whether this single item is likely to add value (if it doesn't, we'll remove it). We've also identified several new items which are not red flags but which appear important predictors in Covid-19 (e.g. myalgia). It may be that even though these items were designed around Covid-19, they will also prove more sensitive and specific in this context than the items in NEWS2 (which was never designed to assess the deteriorating patient in primary care).

There has been a recent study in Uganda in this area that authors might find of interest and would support their proposal [ref]. All the variables identified have been long been recognized as associated with a poor outcome, but in different eras have been associated with different conditions. Recently some of them have been attributed to bacterial sepsis, and now COVID 19. The reality is the variables identified signal possible serious life-threatening illness that needs urgent attention. What the proposed study might determine, and which would be an important contribution to clinical medicine longer after this pandemic is over, is if changes in these variables may occur in advance of catastrophic vital sign changes and, hence, lead to earlier and more effective interventions.

RESPONSE: Yes exactly! NEWS2 has been described as a "pre-mortuary score", too blunt an instrument to detect early deterioration. We are very much hoping that RECAP will allow GPs, Advanced Nurse Practitioners and paramedics to pick patients at a more salvageable stage. As we all know, the disastrous mortality rate in UK at the beginning of this pandemic was due partly to inability to discriminate between those who need urgent escalation of care from those who don't until it was too late.

The Ugandan study is great, thank you, and we will study it carefully as we embark on phase 2 of this research. We've added it as a reference and included in the Discussion.

The score is designed for use on patients with suspected COVID 19. The authors should make it clear if this is not supposed to be used as a diagnostic test. It is an assessment of clinical severity, which might be applied to COVID 19 or any(?) condition. However, the comment in line 24 page 34 of the appendix suggest others do not think this.

RESPONSE: Good point, and we agree this could cause confusion. We've made this clear in the very last paragraph of the paper on page 17, and also added it to the bullet point summary.

What does "suspected COVID 19" mean? A patient with a positive test, or waiting for a positive test, or just someone who someone thinks might have COVID 19? This is important because it is not clear what the response to the score should be, especially for moderate risk patients. It would be wiser to look on this score as a guide to what to do with ANY patient, and not just those suspected to have

COVID 19. Most medical illness starts with the patient having nonspecific feelings of being unwell. The interval between these subjective nonspecific symptoms and the development of specific symptoms and objective signs may be seconds in acute cardiac disease, minutes in meningococcal sepsis, and hours or even days in other conditions. Surely the only thing unique about COVID 19 is that in around 15% of patients a mild illness turns into a serious illness after about 5 days. Therefore, a COVID 19 patient with mild to moderate symptoms might not be in danger for several days, whereas another condition might deteriorate far more rapidly. Therefore, the authors should consider in their Discussion exactly on what kind of patient the score should be performed on (my strong bias is any sick patient) and how often the score should be repeated, and in particular what to do with moderate risk patients (e.g. moderate risk patient repeat in 3 hours?). I am particularly concerned about these patients because they might be sick from something else. These issues should also be considered as part of the validation process.

RESPONSE: We're very much on the same page as the reviewer here, but until we've done the validation study, we don't know! We think it will be generalisable but that's absolutely not how it was designed – the study was designed to produce a Covid-19 specific score. Phase 2 involves data linkage on 2800 patients whose Covid-19 status (clinically diagnosed, swab-diagnosed or antibody-diagnosed) will be part of the dataset. We'll therefore be able to test the hypothesis that the RECAP score, developed to support the care of suspected Covid-19, is actually better than NEWS2 in assessing any sick patient in primary care (especially if done remotely). There's probably another study to be done using RECAP in people with other conditions e.g. suspected sepsis.

Methodology: I am not expert on the techniques used, but they seem entirely reasonable to me. The study purpose was to develop clinical prediction models designed to identify COVID 19 patients who need escalation to next level of care. Was this made clear to all the participants, or did some think they were trying to develop a diagnostic model? (vide supra).

RESPONSE: Thanks, and yes it was made clear to the clinicians. In the focus groups there was a lot of discussion around NEWS2 and developing a score that might be more accurate. So clinical prediction was centre stage. Nobody uses NEWS2 for diagnosis (I hope!). But the reviewer is right to warn that we need to make it really clear that this isn't a diagnostic test. It's also the case that when I analysed the data on the qualitative vignettes, there were two or three (of 72) clinicians whose assessment of the cases was, frankly, clinically concerning. These may have been trainees as we did not require a pedigree to include them in the Delphi (we just asked for experience in seeing Covid-19 patients). I rather suspect that the person who misinterpreted RECAP as a diagnostic test was one of those individuals. Apart from those outliers, everyone on the panel 'got it'.

Minor issue

"…highly sensitive (detecting all patients who need onward referral) and fairly specific (excluding all or most patients who do not)."

Have the authors gotten this the wrong way around? A specific test that is positive rules in a condition (SPPIN) and a sensitive test that is negative rules out a condition (SNNOUT).

RESPONSE: Ah yes, thanks for spotting. Altered (page 5).

Reference

Rice B, Leanza J, Mowafi H, Kamara NT, Mulogo EM, Bisanzo M, Nikam K, Kizza H, Newberry JA, Strehlow M, Global Emergency Care Investigator Group, Kohn M. Defining High-risk Emergency Chief

Complaints: Data-driven Triage for Low- and Middle-income Countries. Acad Emerg Med. 2020 May 16. doi: 10.1111/acem.14013.

Reviewer: 3
Reviewer Name: Abayomi Salawu
Institution and Country: Hull University Teaching Hospital NHS Trust, UK
Please state any competing interests or state 'None declared': No competing Interest

Please leave your comments for the authors below
Typographical errors
Page 6, line 58: Advantages method. There is a preposition and a pronoun missing
Page 13 line 40: Solider

RESPONSE: Many thanks – corrected in revised version (page 7 and 12)

Reviewer: 4
Reviewer Name: Lim Wan Tin
Institution and Country: Singhealth, Singapore
Please state any competing interests or state 'None declared': none declared

Please leave your comments for the authors below
The patient interview group is a small population size. As almost 50% of the score is derived from patient described symptoms, the small population sample in the patient group is a potential limiting factor, leading to higher variability and undercoverage bias. In this case by recruiting patient through social media, there is chance of voluntary response bias. Overall, the study methodology was robust. But the limitation of the study can be better discussed.

RESPONSE: We agree that when we submitted the paper we had only a small patient sample. However since submitting, we've continued to undertake interviews with patients who survived Covid-19. We've carefully been through our dataset and identified a further 30 patients who spoke in detail about their early experiences of acute care when deteriorating. We've added these numbers into the dataset reported in the paper, making a total of 50. The findings did not change any of the items but identified an additional theme: that where there was a mismatch between patient and clinician assessment of severity, it seemed that the clinician tended to conclude that the patient was anxious rather than revisit their own assessment. We've included this additional finding in the results section and in the summary in Box 1.

VERSION 2 – REVIEW

**REVIEWER**	Matthew Sperrin<br>University of Manchester, UK
**REVIEW RETURNED**	08-Oct-2020

**GENERAL COMMENTS**	Thanks for the response to my previous comment. I do agree that the stated aim in the abstract 'to develop items for an early warning score' is appropriate to do using the methods described in the paper. Indeed, everything in the fourth paragraph of the response to my comment (starting 'Incidentally,...') I fully agree with as requiring qualitative assessment. I also fully agree with the authors that a paper that addresses these important aims is well worth publishing as a standalone paper.

	My problem remains that what the conclusions of the paper go beyond this - such as by developing an actual score (e.g. Figure 2). Specifics:<br><br>1.<br>Figure 2 presents an actual score. This is beyond the stated aim of identifying items for the score. Once the items are identified, their relative importance and indeed cut-offs (if one really insists on using cutoffs) should certainly include the use of outcome data and quantitative methods.<br>2.<br>The text that has been added on page 6 'a detailed qualitative phase was considered essential before progressing to a standard validation exercise,' misses out the crucial phase of actually developing a score using outcome data. This concerns me, that it sounds like the next stage in the process is to move directly to validating the score - even though it has not been developed with reference to data.<br>3.<br>In the Discussion's 'five key findings' it is the third that I fundamentally disagree should be addressed as the authors have done here - this actually requires outcome data. The other four findings I am absolutely fine with and fully support.<br>4.<br>In the response to my comment there is an 'action: David'. Therefore please confirm that this has indeed been done.

**REVIEWER**	John Kellett<br>Hospital of South West Jutland<br><br>I am a founder and major shareholder of Tapa Healthcare DAC a start-up medical software company
**REVIEW RETURNED**	07-Oct-2020

**GENERAL COMMENTS**	This is an interesting concept that needs validation.

VERSION 2 – AUTHOR RESPONSE

Reviewer: 2
Reviewer Name: John Kellett
Institution and Country: Hospital of South West Jutland
Please state any competing interests or state ‘None declared’: I am a founder and major shareholder of Tapa Healthcare DAC a start-up medical software company
Comments to the Author
This is an interesting concept that needs validation
no response needed

Reviewer: 1
Reviewer Name: Matthew Sperrin
Institution and Country: University of Manchester, UK
Please state any competing interests or state ‘None declared’: None declared
Comments to the Author

Thanks for the response to my previous comment. I do agree that the stated aim in the abstract 'to develop items for an early warning score' is appropriate to do using the methods described in the paper. Indeed, everything in the fourth paragraph of the response to my comment (starting 'Incidentally,...') I fully agree with as requiring qualitative assessment. I also fully agree with the authors that a paper that addresses these important aims is well worth publishing as a standalone paper.
My problem remains that what the conclusions of the paper go beyond this - such as by developing an actual score (e.g. Figure 2). Specifics:
1. Figure 2 presents an actual score. This is beyond the stated aim of identifying items for the score. Once the items are identified, their relative importance and indeed cut-offs (if one really insists on using cutoffs) should certainly include the use of outcome data and quantitative methods.
On reflection we agree, and have gone through the entire paper to remove any suggestion that this is a “score”. Mostly we’ve changed it to “items”, but we’ve used the term “simulated score” in a couple of places to reflect that we did actually discuss the numerical values in the vignette exercise, though all participants were aware that this was not a definitive score. We’ve renamed figure

2. The text that has been added on page 6 'a detailed qualitative phase was considered essential before progressing to a standard validation exercise,' misses out the crucial phase of actually developing a score using outcome data. This concerns me, that it sounds like the next stage in the process is to move directly to validating the score - even though it has not been developed with reference to data.
we agree, this was an error and we’ve corrected it, see revised text p 6

3. In the Discussion's 'five key findings' it is the third that I fundamentally disagree should be addressed as the authors have done here - this actually requires outcome data. The other four findings I am absolutely fine with and fully support.
we agree, see revised text "a detailed qualitative phase was considered essential before developing the score using actual outcome data and then undertaking a validation exercise"

4. In the response to my comment there is an 'action: David'. Therefore please confirm that this has indeed been done.
yes David did action and we’ve checked again - nothing significant to update

VERSION 3 – REVIEW

**REVIEWER**	Matthew Sperrin<br>University of Manchester, UK
**REVIEW RETURNED**	03-Nov-2020

**GENERAL COMMENTS**	Thanks for responding to my second round of comments: I am happy with the responses.
